# Pyronaridine-Artesunate versus Chloroquine in Patients with Acute *Plasmodium vivax* Malaria: A Randomized, Double-Blind, Non-Inferiority Trial

**DOI:** 10.1371/journal.pone.0014501

**Published:** 2011-01-18

**Authors:** Yi Poravuth, Duong Socheat, Ronnatrai Rueangweerayut, Chirapong Uthaisin, Aung Pyae Phyo, Neena Valecha, B. H. Krishnamoorthy Rao, Emiliana Tjitra, Asep Purnama, Isabelle Borghini-Fuhrer, Stephan Duparc, Chang-Sik Shin, Lawrence Fleckenstein

**Affiliations:** 1 National Malaria Center, Phnom Penh, Cambodia; 2 Department of Internal Medicine, Mae Sot General Hospital, Mae Sot, Thailand; 3 Department of Internal Medicine, Mae Ramat Hospital, Mae Ramat, Thailand; 4 Shoklo Malaria Research Unit, Mae Sot, Thailand; 5 National Institute of Malaria Research, Delhi, India; 6 Wenlock District Hospital, Kasturba Medical College, Mangalore, India; 7 National Institute of Health Research and Development, Ministry of Health, Jakarta, Indonesia; 8 Internal Medicine Department, TC Hillers General Hospital, Maumere, Indonesia; 9 Medicines for Malaria Venture, Geneva, Switzerland; 10 Shin Poong Pharmaceutical Company, Seoul, Republic of Korea; 11 College of Pharmacy, University of Iowa, Iowa City, Iowa, United States of America; Menzies School of Health Research, Australia

## Abstract

**Background:**

New antimalarials are needed for *P. vivax* and *P. falciparum* malaria. This study compared the efficacy and safety of pyronaridine-artesunate with that of chloroquine for the treatment of uncomplicated *P. vivax* malaria.

**Methods and Findings:**

This phase III randomized, double-blind, non-inferiority trial included five centers across Cambodia, Thailand, India, and Indonesia. In a double-dummy design, patients (aged >3–≤60 years) with microscopically confirmed *P. vivax* mono-infection were randomized (1∶1) to receive pyronaridine-artesunate (target dose 7.2∶2.4 mg/kg to 13.8∶4.6 mg/kg) or chloroquine (standard dose) once daily for three days. Each treatment group included 228 randomized patients. Outcomes for the primary endpoint, Day-14 cure rate in the per-protocol population, were 99.5%, (217/218; 95%CI 97.5, 100) with pyronaridine-artesunate and 100% (209/209; 95%CI 98.3, 100) with chloroquine. Pyronaridine was non-inferior to chloroquine: treatment difference −0.5% (95%CI −2.6, 1.4), i.e., the lower limit of the 2-sided 95%CI for the treatment difference was greater than −10%. Pyronaridine-artesunate cure rates were non-inferior to chloroquine for Days 21, 28, 35 and 42. Parasite clearance time was shorter with pyronaridine-artesunate (median 23.0 h) versus chloroquine (32.0 h; p<0.0001), as was fever clearance time (median 15.9 h and 23.8 h, respectively; p = 0.0017). Kaplan-Meier estimates of post-baseline *P. falciparum* infection incidence until Day 42 were 2.5% with pyronaridine-artesunate, 6.1% with chloroquine (p = 0.048, log-rank test). Post-baseline *P. vivax* or *P. falciparum* infection incidence until Day 42 was 6.8% and 12.4%, respectively (p = 0.022, log rank test). There were no deaths. Adverse events occurred in 92/228 (40.4%) patients with pyronaridine-artesunate and 72/228 (31.6%) with chloroquine. Mild and transient increases in hepatic enzymes were observed for pyronaridine-artesunate.

**Conclusion:**

Pyronaridine-artesunate efficacy in acute uncomplicated *P. vivax* malaria was at least that of chloroquine. As pyronaridine-artesunate is also efficacious against *P. falciparum* malaria, this combination has potential utility as a global antimalarial drug.

**Trial registration:**

Clinicaltrials.gov NCT00440999

## Introduction

An estimated 2.85 billion people are at risk of *Plasmodium vivax* malaria, with up to 400 million clinical cases annually [1]–[4]. Recent reports also indicate that the severity of *P. vivax* malaria had been underappreciated; it can even be fatal [1], [2], [5]–[7]. Around 91% of those at risk of *P. vivax* malaria live in the tropical areas across South and South East Asia, the remainder mostly live in the Western Pacific and Eastern Mediterranean [3], [4]. The re-emergence of *P. vivax* in areas where it had previously been eradicated, such as Korea and China, is of major concern [8]–[10].

The blood schizontocide chloroquine and the tissue schizontocide primaquine have been the mainstay of *P. vivax* treatment in most areas of the world for the past 50 years. However, *P. vivax* susceptibility to chloroquine has declined in some regions, (e.g. Indonesia, Peru and Oceania) and new approaches are required urgently [11], [12].

The blood stage of *Plasmodium vivax* is highly sensitive to artemisinins [13], [14]. However, these agents cannot be used alone because their short half-life can lead to recrudescence, and potentially the development of drug resistance [15], [16]. Thus, in areas of chloroquine-resistant *P. vivax*, artemisinin-based combination therapy (ACT) is recommended by The World Health Organization [17]. ACT is also generally recommended by the WHO for the treatment of *P. falciparum* malaria [17]. In areas where *P. vivax* and *P. falciparum* are sympatric, ACT should have good efficacy (>95%) against both parasites, as differential diagnosis may not be feasible or reliable [12], [18], and mixed infections may also be present [12], [19].

Pyronaridine-artesunate is being developed as an ACT for use against *P. vivax* and *P. falciparum* malaria. Pyronaridine is especially interesting as a component of ACT because *in vitro* studies using recent isolates indicate potent activity against chloroquine-resistant *P. vivax* and *P. falciparum* [20]. Pyronaridine has been used in China for over 30 years, and clinical trials of pyronaridine monotherapy indicated efficacy against *P. vivax* similar to that of chloroquine [21], [22]. In recent Phase II/III clinical trials of fixed-dose pyronaridine-artesunate (3∶1 ratio) conducted in Africa and Asia, efficacy rates in *P. falciparum* malaria at Day 28 were >99%, and the combination was generally well tolerated [23], [24].

This is the first pyronaridine-artesunate Phase III study reported for the treatment of *P. vivax* malaria. The primary objective of this clinical study was to compare the efficacy and safety of the fixed combination of pyronaridine-artesunate with that of standard chloroquine therapy in adults and children with acute, uncomplicated *P. vivax* malaria.

## Methods

The protocol for this trial and supporting CONSORT checklist are available as supporting information; see Checklist S1 and Protocol S1.

### Ethics statement

This study was conducted according to the principals of Good Clinical Practice and the Declaration of Helsinki and complied with all relevant regulatory requirements. The trial protocol was approved by institutional review boards in Cambodia (Ministry of Health, National Ethics Committee for Health Research), Thailand (Faculty of Tropical Medicine, Mahidol University Ethics Committee and Ministry of Public Health, Ethical Review Committee for Research in Human Subjects), India (Institutional Ethics Committee, National Institute of Malaria Research, India Council of Medical Research), and Indonesia (Ministry of Health, National Institute of Health Research and Development). All patients or their guardians provided written informed consent and assent was required from children old enough to understand the study.

### Design and setting

This was a multi-center, randomized, double-blind, double-dummy, parallel-group comparative, non-inferiority trial conducted between March 2007 and March 2008 in local hospitals across five centers: Pailin, Cambodia; Mae Sot and Mae Ramat, Thailand; Mangalore, India; and Maumere (Island of Flores), Indonesia. There were no changes to the protocol after study commencement.

### Participants

Male or female subjects between three and 60 years of age with acute uncomplicated *P. vivax* mono-infection confirmed by positive microscopy of *P. vivax* with a parasite density ≥250 µL^−1^ of blood (including at least 50% asexual parasites) and fever or documented history of fever in the previous 24 h, and with a body weight between 20 and 90 kg (with no clinical evidence of severe malnutrition) were eligible for enrollment. Patients were excluded from the study if they had: any other condition requiring hospitalization; anemia (hemoglobin <8 g/dL); hepatic or renal impairment; presence or history of clinically significant disorders; known hypersensitivity to study drugs or excipients; known active hepatitis A IgM, hepatitis B surface antigen, hepatitis C antibody or seropositive for HIV antibody; used an antimalarial within the previous two weeks (urine test required); used an antibacterial with anti-malarial activity within the previous two weeks; received an investigational drug within the past four weeks; previously participated in the study. Women who were pregnant or lactating were also excluded. Agreement to use an acceptable method of contraception during the study was required from all women of child-bearing age.

### Interventions

Study drugs were pyronaridine-artesunate tablets 180∶60 mg, chloroquine tablets 155 mg, and matching placebos (all provided by Shin Poong Pharmaceutical Co., Ltd., Seoul, Korea). Study drugs were given orally with water once daily for three days (Days 0, 1, and 2) under direct observation of study staff. Vomiting of the first dose of study drug within 30 minutes of administration resulted in re-dosing. Vomiting of subsequent doses resulted in withdrawal from the study and treatment with rescue medication (as per local practice). For pyronaridine-artesunate, drug dose was based on body weight: 20–25 kg, 1 tablet; 26–44 kg, 2 tablets; 45–64 kg, 3 tablets; and 65–90 kg, 4 tablets, i.e. a pyronaridine-artesunate target dose of between 7.2∶2.4 mg/kg and 13.8∶4.6 mg/kg. The chloroquine dose for adults was 620 mg on Days 0 and 1 and 310 mg on Day 2. The chloroquine target dose for children was 10 mg/kg on Days 0 and 1 and 5 mg/kg on Day 2.

All subjects remained hospitalized for Days 0–3, returning for follow-up assessments on Days 7, 14, 21, 28, 35 and 42. A medical history was taken at screening and physical examination performed at screening, Days 3, 28 and 42. Vital signs were monitored at screening, on Days 1, 2, 3 and 7 and at other visits if indicated. Clinical signs and symptoms of malaria were assessed at screening, Days 1, 2 and 3 and at other visits if indicated. Body temperature was taken every 8 h over at least 72 h following the first study drug administration or temperature normalization for at least two readings 7–25 h apart, then at each visit. Venous blood was drawn for hematology at screening, Days 3, 7, 28, and 42, and for clinical laboratory tests at screening, Days 3, 7, and 28, and Day 42 if clinically indicated. Urinalysis was performed on the same schedule. Adverse events were monitored throughout the study. 12-lead electrocardiograms were performed at screening, Day 2 and at Days 7, 14 and 42 if clinically indicated.

Blood samples for testing glucose-6-phosphate dehydrogenase (G6PD) deficiency were collected on pre-printed filter paper. A semi-quantitative fluorescent spot test was used to distinguish between G6PD-deficient samples and those with normal or intermediate activity (G-6-PD, R&D Diagnostics Ltd, Holargos, Greece). Patients completing the study to Day 28 with normal G6PD activity were given a 14-day course of primaquine (15 mg/day for adults and 0.3 mg/kg/day for children) starting on Day 28 after all required assessments had been performed. G6PD-deficient patients completing the study to Day 28 were treated as per country policy.

Parasite density was determined by duplicate Giemsa-stained thick blood films examined independently by two microscopists; the mean of the two readings was recorded. In the case of discrepancy (i.e. >30%), a third microscopist reviewed the slides and the principal investigator made the final classification. Thick films were taken every 8 h until at least 72 h or until a negative smear was recorded at least 7–25 h apart, then at each visit, or if the patient returned unscheduled. Asexual parasite and gametocyte counts were recorded separately at screening; total parasite counts were recorded at all other assessments. Parasites were enumerated against 200 white blood cells (WBC) using 1,000× magnification. When the number of asexual parasites dropped below 10 per 200 WBC, counting was done against at least 500 WBC. A blood slide was considered negative if no asexual parasites were observed per 1,000 WBC. External quality control, was conducted by a central independent laboratory, blinded to treatment (Swiss Tropical Institute, Basel, Switzerland). All slides from patients with treatment failure were re-examined, plus the first 5 slides from each site and 5% of all subjects randomly. Results confirmed that procedures were being conducted as per the defined protocol. To determine *Plasmodium* species, duplicate thin blood smears were taken at screening, pre-dose on Day 1 and at visits from Day 7 whenever parasitological blood samples were taken. Patients experiencing treatment failure were administered rescue antimalarial therapy as per local practice. All procedures scheduled for Day 28, including preparation of thin and thick films were performed before rescue medication was administered.

### Objectives

The primary objective of this clinical study was to compare the efficacy and safety of the fixed combination of pyronaridine-artesunate (180∶60 mg) with that of standard chloroquine therapy in adults and children with acute, uncomplicated *P. vivax* malaria.

### Outcomes

Efficacy endpoints followed WHO 2002 guidelines for monitoring antimalarial drug efficacy, as were relevant at the time that the study was designed [25]. As there are no validated molecular tools, the primary efficacy endpoint was the cure rate on Day 14. Cure at any given evaluation was defined as aparasitemia, irrespective of body temperature, without previous treatment failure. Treatment failure was defined as either clinical deterioration caused by *P. vivax* illness requiring hospitalization in the presence of parasitemia; or presence of parasitemia and axillary temperature ≥37.5°C any time between Days 3 and 14; or presence of parasitemia on any day between Days 7 and 14, irrespective of clinical condition. Subjects presenting with non-*P. vivax* malaria after clearance of *P. vivax* parasites were withdrawn from the study and given treatment for the new infection. If non-*P. vivax* malaria occurred on or after Day 14, the subject was considered a success for Day 14.

Secondary efficacy measures included: Day 28 cure rate; parasite clearance time, defined as the time from first treatment dose to aparasitemia for two consecutive negative readings taken between 7–25 h apart; fever clearance time, defined as time from first treatment dose to apyrexia for two consecutive normal temperature readings taken between 7–25 h apart; the proportion of patients aparasitemic on Days 1, 2, and 3; and the proportion of patients apyretic on Days 1, 2, and 3. The cure rates on Days 21, 35 and 42 were included as exploratory endpoints.

Safety outcomes included: the frequency, nature and severity of adverse events; the frequency of serious adverse events; the frequency of adverse events considered by the investigator to be related to study medication; the reasons for study withdrawals; abnormal electrocardiograms; and abnormal hematology and clinical chemistry results. An adverse event was defined as any unfavorable and unintended sign, symptom, syndrome, or illness that developed or worsened during the period of observation in the clinical study. A serious adverse event was defined as one that resulted in: death or was life threatening; hospitalization or a prolongation of hospitalization; a congenital abnormality or birth defect; a persistent or significant disability or incapacity; or was judged by the investigator to be serious. Drug-related adverse events were determined by the investigator, based on the temporal relationship to drug therapy, any corroborating laboratory data, and improvement when drug was discontinued.

### Sample size

Assuming a 90% cure rate on Day 14 in both treatment groups and a non-inferiority limit of −10%, a sample size of 410 evaluable subjects randomized in a 1∶1 ratio (205 subjects in each treatment group) would provide 90% power to demonstrate non-inferiority of pyronaridine-artesunate compared to chloroquine, using a 2-sided 95% confidence interval (CI). Assuming a dropout rate of 10%, 456 subjects would need to be randomized to the study (228 subjects in each treatment group). Assuming a Day-14 cure rate of 95%, this sample size would provide >99% power to demonstrate non-inferiority of pyronaridine-artesunate compared to chloroquine.

### Randomization and blinding

A computer-generated randomization scheme was provided by the sponsor. Subjects were randomized 1∶1 within each study site in blocks of six to receive either pyronaridine-artesunate plus matching chloroquine placebo or oral chloroquine plus matching pyronaridine-artesunate placebo. Randomization numbers were assigned in ascending order to each subject according to the order recruited. The subject was allocated an individually numbered treatment pack, which contained sufficient tablets for 3 days' therapy plus an overage bottle containing tablets in case the subject vomited the first dose. Study drugs were administered on a double-blind, double-dummy basis. The investigator calculated the appropriate dose and study drug was administered by a different member of staff, designated by the investigator. All study investigators, laboratory technicians and patients were blind to treatment assignment. Active drugs and placebos were packaged similarly. Sealed opaque envelopes containing the study medication assignment for each subject were provided to the study site investigator for use in an emergency; no code breaks were required.

### Statistical methods

The intent-to-treat (ITT) population was defined as all randomized subjects who received any amount of study medication. The per-protocol (PP) population included all randomized patients who completed a full course of study medication, had a known primary efficacy endpoint at Day 14 and did not violate the protocol in a way that would compromise efficacy evaluation, i.e. did not use prohibited concomitant medication; have a concomitant medical condition or infection that may have affected treatment outcome; or have any major protocol deviation.

For this non-inferiority study, the primary efficacy endpoint of Day-14 cure rate was assessed in the PP population. Non-inferiority was demonstrated if the lower limit of the 2-sided 95% confidence interval (CI) for the difference in Day-14 cure rates was greater than –10%. The CI was calculated according to the Newcombe–Wilson method without continuity correction. Furthermore, exact (Pearson–Clopper) 95% CIs were calculated for the Day-14 cure rate in each of the two treatment groups. A similar analysis was repeated for the proportion of subjects with cure on Days 21, 28, 35 and 42.

Parasite clearance time and fever clearance time were summarized using Kaplan-Meier estimates (log-rank test). Subjects without confirmed parasite clearance time or fever clearance time within 72 h after the first dose of study drug were censored at that time point.

Post-hoc analyses of time to *P. falciparum* infection and for time to reappearance of any parasite (*P. vivax* or *P. falciparum*) were also conducted using Kaplan-Meier estimates. All statistical evaluations were performed using SAS®, Version 9.1.3 in a UNIX environment.

## Results

### Participant flow

**Figure 1:**
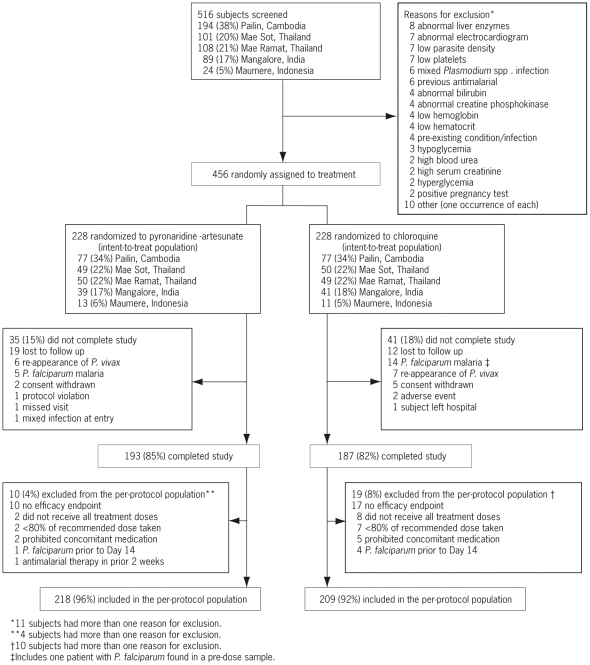
Trial flow and subject disposition.

Patient disposition is shown in Figure 1. The randomized population included 456 patients, 228 in each treatment group. All randomized patients received at least one dose of study drug and were included in the ITT population. Most patients (83.3%) completed the study. A similar number of patients withdrew prematurely from the study in both groups. However, in the chloroquine group 14/228 (6.1%) patients withdrew because of *P. falciparum* infection compared with 5/228 (2.2%) in the pyronaridine-artesunate group. More patients were excluded from the PP population in the chloroquine group, 19/228 (8.3%), than the pyronaridine-artesunate group, 10/228 (4.4%), mainly because of missing efficacy data and insufficient drug therapy.

Patient baseline demographic and clinical characteristics are shown in Table 1. All subjects were Asian/Oriental and mostly male (73.7%). Mean age was 26.7 years. Clinical characteristics were similar between the two treatment groups. A similar proportion of patients in each treatment group received study drug dose as planned; 225/228 (98.7%) in the pyronaridine-artesunate group and 214/228 (93.9%) in the chloroquine group. G6PD deficiency was detected in 16/228 (7.0%) of patients in each treatment group. Primaquine was administered to 185/228 (87.3%) patients in the pyronaridine-artesunate group and 181/228 (85.4%) in the chloroquine group starting on Day 28 of the study.

**Table 1:** Baseline demographic and clinical data of the randomized (intent-to-treat) population.

Characteristic	Pyronaridine-artesunate (N = 228)	Chloroquine (N = 228)
Male sex, n (%)	172 (75.4)	164 (71.9)
Mean age, years (SD) [range]	27.0 (11.2) [7 to 60]	26.4 (10.9) [7 to 58]
Number aged ≤12 years, n (%)	14 (6.1)	13 (5.7)
Number aged >12 years, n (%)	214 (93.9)	215 (94.3)
Mean weight, kg (SD) [range]	49.2 (9.9) [20.0 to 68.4]	49.4 (10.2) [20.0 to 80.0]
*P. vivax* asexual forms, n (%)	228 (100)	228 (100)
Geometric mean asexual parasitemia, µL^−1^ [range]	6914.8 [466 to 92500]	6145.8 [366 to 77035]
*P. vivax* gametocytes, n (%)	192 (84.2)	181 (79.4)
Geometric mean gametocyte parasitemia, µL^−1^ [range]	1106.2 [0 to 41277]	915.3 [0 to 29765]
Temperature, °C (SD) [range]	38.0 (1.0) [35.9 to 40.6]	38.0 (0.9) [36.0 to 40.5]
Patients with fever, n (%)	108 (47.4)	107 (46.9)
Malaria in prior 12 months, n (%)		
None	129 (56.6)	123 (53.9)
One	42 (18.4)	58 (25.4)
Two	31 (13.6)	22 (9.6)
More than two	26 (11.4)	25 (11.0)
*P. vivax* infection in prior 12 months, n (%)		
None	166 (72.8)	175 (76.8)
One	26 (11.4)	25 (11.0)
Two	25 (11.0)	15 (6.6)
More than two	11 (4.8)	13 (5.7)

All patients were of Asian/Oriental ethnicity.

### Outcomes and estimation

**Figure 2:**
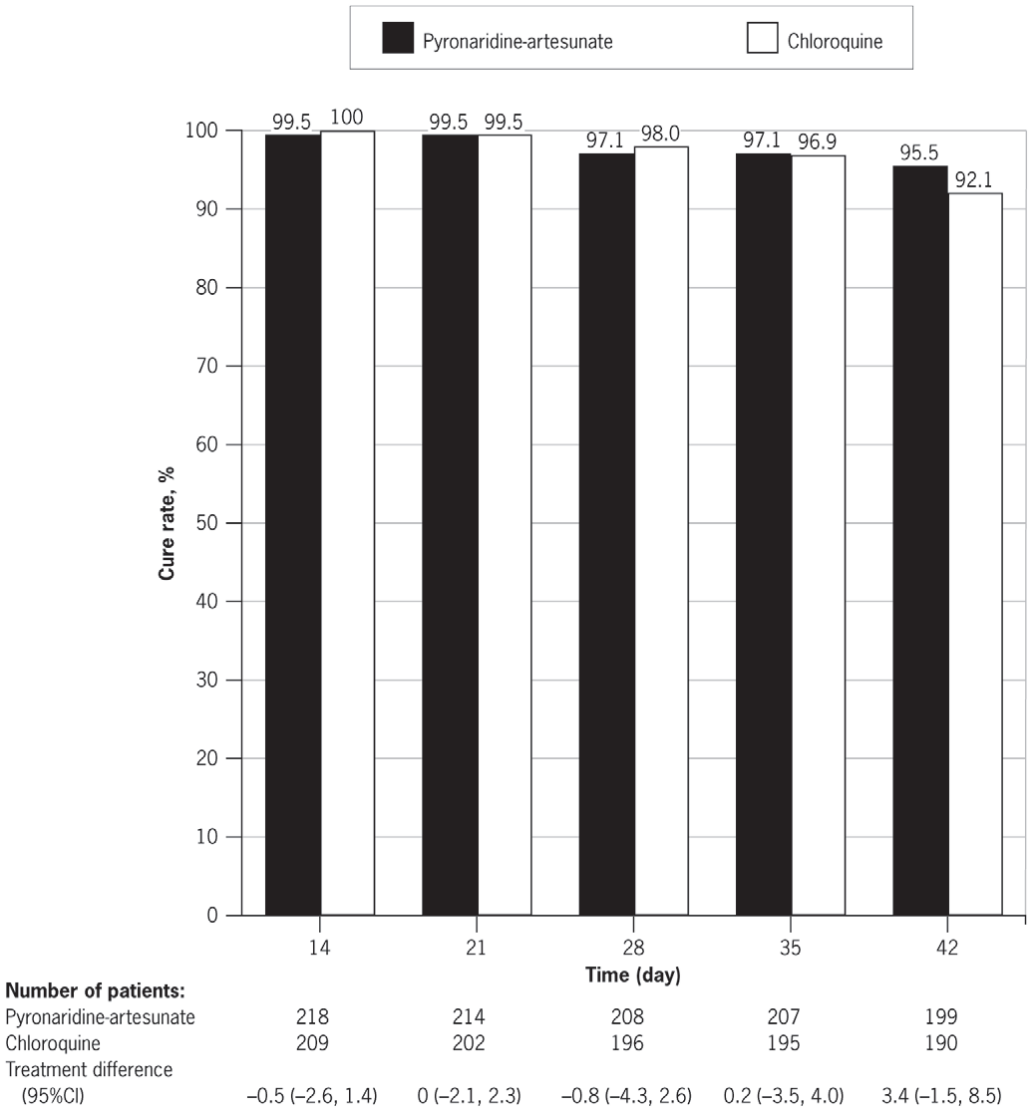
Cure rate for *P. vivax* malaria on Days 14, 21, 28, 35, and 42 in the per-protocol population. Non-inferiority of pyronaridine-artesunate to chloroquine was concluded for all assessments.

Outcomes for the primary endpoint, cure rate at Day 14 in the PP population, were 99.5%, (217/218; 95%CI 97.5, 100) in the pyronaridine-artesunate group and 100% (209/209; 95%CI 98.3, 100) in the chloroquine group. Pyronaridine was non-inferior to chloroquine for the primary endpoint: treatment difference −0.5% (95%CI −2.6, 1.4). All 27 children ≤12 years were cured at Day 14. Day-14 cure rates were 100% in all study centers, except for the one failure in the pyronaridine-artesunate group, which occurred in a patient from Maumere, Indonesia (90.9% cure rate [10/11]; 95%CI 58.7, 99.8). Results for Day-14 cure rate in the ITT population were supportive of the primary analysis; 95.2%; (217/228; 95%CI 91.5, 97.6) with pyronaridine-artesunate and 93.0% (212/228; 95%CI 88.9, 95.9) with chloroquine, treatment difference 2.2% (95%CI −2.3, 6.8). At all efficacy assessments from Day 14 until Day 42, pyronaridine-artesunate was non-inferior to chloroquine, with cure rates >95% (Figure 2).

**Figure 3:**
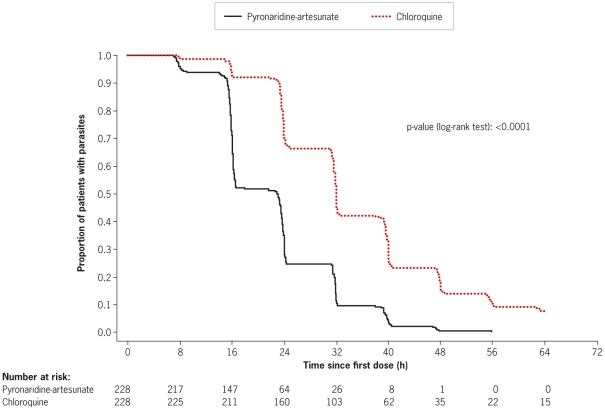
Kaplan-Meier estimates of *P. vivax* parasite clearance time (h) (intent-to-treat population).

Using Kaplan–Meier analysis of the ITT population, time to parasite clearance was shorter in the pyronaridine-artesunate group compared with the chloroquine group (p<0.0001; log-rank test; Figure 3). Median time to parasite clearance was 23.0 h with pyronaridine-artesunate and 32.0 h with chloroquine (Table 2). Time to parasite clearance was faster with pyronaridine-artesunate at all individual centers (Table 2). Using Kaplan–Meier estimates, by 24 h after the first dose of study drug, more patients treated with pyronaridine-artesunate achieved parasite clearance than with chloroquine: 71.9% (95%CI 66.1, 77.8) and 29.8% (95%CI 23.9, 35.8), respectively. By 48 h post first-dose, 99.6% (95%CI 98.7, 100.0) of patients receiving pyronaridine-artesunate were aparasitic compared with 84.6% (95%CI 80.0, 89.3) receiving chloroquine. By 72 h, in the pyronaridine-artesunate group 100% (95%CI 100, 100) of patients were aparasitic compared with 93.4% (95%CI 90.2, 96.6) in the chloroquine group.

**Table 2:** Median parasite clearance time for *P. vivax* (intent-to-treat population).

Center	N	Pyronaridine-artesunate	N	Chloroquine	p value*
Pailin, Cambodia	77	16.4 (16.1, 23.5) [7.3 to 40.1]	77	32.0 (31.9, 39.4) [16.0 to 63.9]	<0.0001
Mae Sot, Thailand	49	22.6 (15.8, 23.3) [14.0 to 47.3]	50	34.8 (24.8, 39.5) [14.8 to 63.4]	<0.0001
Mae Ramat, Thailand	50	16.2 (15.8, 23.8) [7.7 to 40.3]	49	31.7 (31.3, 32.1) [15.8 to 63.4]	<0.0001
Mangalore, India	39	23.5 (15.9, 24.3) [7.0 to 47.8]	41	32.0 (24.0, 39.5) [7.5 to 56.5]	0.0002
Maumere, Indonesia	13	31.9 (24.0, 39.8) [23.6 to 55.9]	11	47.9 (32.0, 63.9) [23.9 to 63.9]	0.0119
All centers	228	23.0 (16.3, 23.5) [7.0 to 55.9]	228	32.0 (31.8, 32.2) [7.5 to 63.9]	<0.0001

Data are median time to parasite clearance (95%CI) [range]. Units are hours.

Ranges do not include censored times.

*Log-rank test.

**Figure 4:**
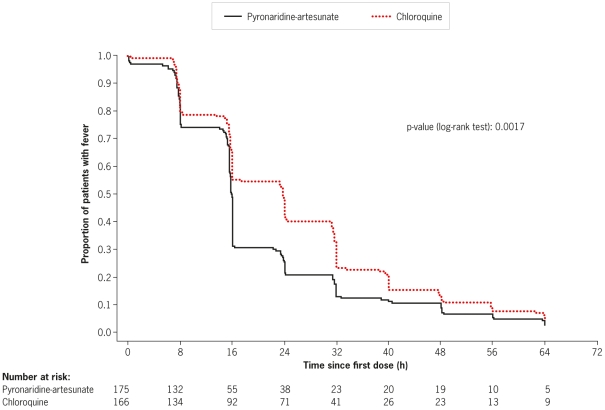
Kaplan-Meier estimates of fever clearance time (h) in patients with *P. vivax* malaria (intent-to-treat population).

Concomitant anti-pyretics were taken by 190/228 (83.3%) patients in the pyronaridine-artesunate group and 179/228 (78.5%) patients in the chloroquine group. Therefore, evaluation of the anti-pyretic activity of pyronaridine-artesunate and chloroquine cannot be fully assessed. However Kaplan-Meier analysis of the ITT population showed a shorter time to fever clearance with pyronaridine-artesunate than with chloroquine (p = 0.0017; Figure 4). Median time to fever clearance was 15.9 h (95%CI 15.7, 16.0) with pyronaridine-artesunate and 23.8 h (95%CI 16.0, 24.0) with chloroquine. Using Kaplan–Meier estimates, more patients receiving pyronaridine-artesunate had achieved fever clearance by 24 h after the first dose of study medication, 78.3% (95%CI 72.2, 84.4), than with chloroquine, 57.2% (95%CI 49.7, 64.8). The number of patients with fever clearance was similar between the two treatment groups at 48 h post-first dose: 89.1% (95%CI 84.5, 93.8) for pyronaridine-artesunate and 86.1% (95%CI 80.9, 91.4) for chloroquine; and at 72 h post-first dose: 97.1% (95%CI 94.7, 99.6) and 95.2% (95%CI 91.9, 98.4), respectively.

### Ancillary analyses

#### Time to *P. falciparum* infection

**Figure 5:**
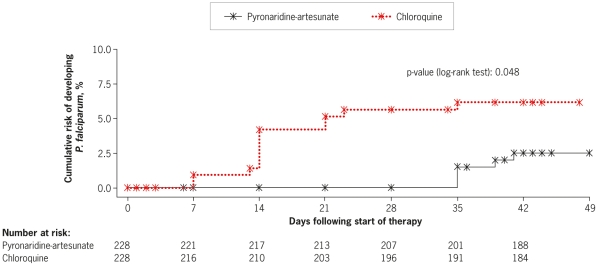
Kaplan-Meier estimates of time (h) to *P. falciparum* infection in patients treated for an initial *P. vivax* malaria (intent-to-treat population).

Five patients in the pyronaridine-artesunate group and 13 patients in the chloroquine group developed a *P. falciparum* infection during the study. Corresponding Kaplan-Meier estimates for the probability of developing such an infection until Day 42 were 2.5% and 6.1%, respectively (p = 0.048, log rank test, Figure 5). All five *P. falciparum* infections in the pyronaridine-artesunate group occurred between Days 35 and 41. In the chloroquine group, 10/13 of the *P. falciparum* infections occurred on or before Day 14 (Figure 5).

#### Time to *P. vivax* or *P. falciparum* infection

**Figure 6:**
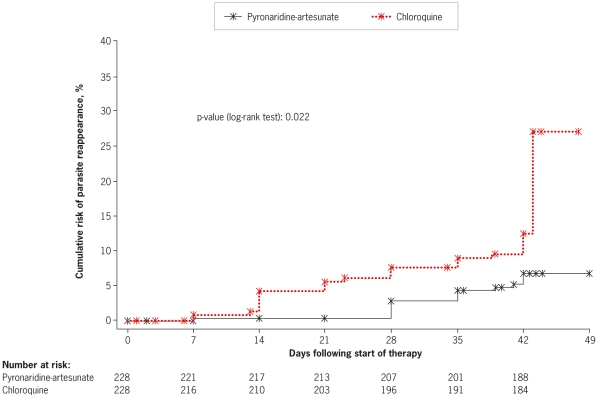
Kaplan-Meier estimates of time (h) to *P. vivax* or *P. falciparum* infection in patients treated for an initial *P. vivax* malaria (intent-to-treat population).

A total of 14 patients in the pyronaridine-artesunate group and 28 patients in the chloroquine group developed a *P. vivax* or *P. falciparum* infection during the study. Corresponding Kaplan-Meier estimates for the probability of developing such an infection until Day 42 were 6.8% and 12.4%, respectively (p = 0.022, log rank test, Figure 6).

### Adverse events

In the pyronaridine-artesunate group, 92/228 (40.4%) patients experienced a treatment-emergent adverse event of any cause compared with 72/228 (31.6%) in the chloroquine group (Table 3). Increased transaminases were more common in the pyronaridine-artesunate group (2.2%) versus the chloroquine group (0%, Table 3). All adverse events in the pyronaridine-artesunate group and the majority (70/72; 97.2%) in the chloroquine group were of mild-to-moderate severity. Adverse events deemed study drug related by the investigator occurred in 27/228 (11.8%) patients in the pyronaridine-artesunate group and 23 (10.1%) in the chloroquine group (Table 3).

**Table 3:** Treatment-emergent adverse events in the safety (intent-to-treat) population.

	Pyronaridine-artesunate (N = 228)	Chloroquine (N = 228)	Treatment difference (95%CI)
**Adverse events of any cause** *			
Patients with at least one event	92 (40.4)	72 (31.6)	8.8 (0.0, 17.5)
Headache	45 (19.7)	34 (14.9)	4.8 (−2.1, 11.8)
Myalgia	30 (13.2)	21 (9.2)	3.9 (−1.8, 9.7)
Anorexia	19 (8.3)	10 (4.4)	3.9 (−0.5, 8.4)
Nasopharyngitis	13 (5.7)	6 (2.6)	3.1 (−0.6, 6.7)
Fatigue	12 (5.3)	11 (4.8)	0.4 (−3.6, 4.5)
Blood CPK increased	9 (3.9)	8 (3.5)	0.4 (−3.0, 3.9)
Transaminases increased	5 (2.2)	0 (0.0)	2.2 (0.3, 4.1)
Cough	5 (2.2)	5 (2.2)	0 (−2.7, 2.7)
Dizziness	3 (1.3)	6 (2.6)	−1.3 (−3.9, 1.2)
Vomiting	2 (0.9)	7 (3.1)	−2.2 (−4.7, 0.4)
Electrocardiogram QT prolonged	1 (0.4)	6 (2.6)	−2.2 (−4.4, 0.1)
**Drug-related adverse events** †			
Patients with at least one event	27 (11.8)	23 (10.1)	1.8 (−4.0, 7.5)
Headache	9 (3.9)	3 (1.3)	2.6 (−0.3, 5.6)
Anorexia	6 (2.6)	2 (0.9)	1.8 (−0.7, 4.2)
Blood CPK increased	5 (2.2)	6 (2.6)	−0.4 (−3.3, 2.4)
Fatigue	3 (1.3)	1 (0.4)	0.9 (−0.8, 2.6)
Dizziness	2 (0.9)	5 (2.2)	−1.3 (−3.6, 0.9)
Vomiting	1 (0.4)	4 (1.8)	−1.3 (−3.2, 0.6)

Data are number (%) unless otherwise indicated. CPK, creatine phosphokinase.

*Adverse events that occurred in ≥2% of patients in either treatment group.

†Drug-related adverse events that occurred in ≥1% of patients in either treatment group.

There were no deaths during the study. Two patients, both in the pyronaridine-artesunate group, had serious adverse events (one pyrexia, one typhoid fever); neither was considered drug related by the investigator. Two patients, both in the chloroquine group, had adverse events leading to drug discontinuation and study withdrawal (one vomiting plus fatigue, one vomiting); all considered possibly related to drug treatment by the investigator.

Baseline hemoglobin levels were similar in the two treatment groups (Table 4). A transient decrease in mean hemoglobin levels was observed in both treatment groups, though this was greater at Days 3 and 7 in the pyronaridine-artesunate group (Table 4). No clinically meaningful difference in change from baseline in hemoglobin was observed between patients with or without phenotypic G6PD deficiency. Other hematology laboratory findings (increases in platelets, eosinophils, and lymphocytes and a decrease in neutrophils) were of similar magnitude in both treatment groups.

**Table 4:** Baseline values and change from baseline at Days 3, 7 and 28 for key laboratory measures and incidence of post-baseline Grade 3 or 4 toxicity values.

Parameter	Day	N	Pyronaridine-artesunate	N	Chloroquine
**Hb, g/L**	Baseline	228	125.3 (17.0) [70 to 166]	228	123.4 (18.4) [71 to 159]
	Day 3	226	−5.5 (8.5) [−33 to 18]	222	−3.7 (8.9) [−24 to 37]
	Day 7	219	−6.0 (9.1) [−36 to 22]	215	−0.7 (10.0) [−36 to 47]
	Day 28	203	2.9 (11.9) [−45 to 46]	203	5.7 (11.9 [−38 to 42]
	Grade 3*	228	1 (0.4)	228	3 (1.3)
**ALT, U/L**	Baseline	228	24.5 (19.1) [7 to 177]	228	25.2 (16.2) [3 to 106]
	Day 3	226	2.8 (20.8) [−106 to 132]	222	−1.3 (12.9) [−46 to 71]
	Day 7	219	17.2 (50.0) [−73 to 447]	215	−1.3 (14.2) [−58 to 56]
	Day 28	203	−3.5 (16.2) [−98 to 72]	203	−1.9 (15.9) [−66 to 87]
	Grade 3/4	228	3 (1.3)	228	0
**AST, U/L**	Baseline	228	30.6 (17.0) [6 to 138]	228	31.5 (16.0) [6 to 143]
	Day 3	226	0.7 (23.7) [−102 to 144]	222	−5.4 (11.9) [−69 to 43]
	Day 7	219	4.7 (28.8) [−83 to 317]	215	−4.3 (12.8) [−65 to 28]
	Day 28	203	−1.1 (14.7) [−105 to 52]	203	−0.7 (18.5) [−66 to 172]
	Grade 3/4	228	1 (0.4)	228	1 (0.4)
**ALP, U/L**	Baseline	228	98.3 (54.9) [26 to 385]	228	99.5 (54.5) [31 to 517]
	Day 3	222	2.2 (54.7) [−186 to 667]	217	−4.0 (30.3) [−161 to 140]
	Day 7	213	7.5 (42.4) [−106 to 361]	211	−1.3 (37.9) [−295 to 153]
	Day 28	194	1.7 (37.5) [−175 to 123]	198	−0.2 (42.4) [−201 to 170]
	Grade 3*	199	1 (0.5)	199	0
**TBIL, µmol/L**	Baseline	228	23.9 (14.5) [3.1 to 102.6]	228	23.0 (13.5) [3.4 to 94.1]
	Day 3	226	−13.4 (13.4) [−83.8 to 13.7]	222	−11.7 (11.9) [−66.7 to 8.6]
	Day 7	219	−14.3 (14.0) [−85.5 to 12.0]	215	−11.5 (10.3) [−70.1 to 8.6]
	Day 28	203	−12.8 (14.0) [−80.4 to 17.1]	203	−11.1 (12.0) [−78.7 to 12.0]
	Grade 3*	228	0	228	1 (0.4)

Data are mean (SD) [range] or number (%).

*No Grade 4 toxicities. Hb, hemoglobin; ALT, alanine aminotransferase; AST, aspartate aminotransferase; ALP, alkaline phosphatase; TBIL, total bilirubin. Grade 3 toxicity: Hb (65–79 g/L); ALT/AST/ALP (5.1–10×ULN); TBIL (2.6–5×ULN). Grade 4 toxicity: ALT/AST (>10×ULN). ULN, upper limit of normal.

Biochemistry laboratory observations were generally similar in both treatment groups, with the exception of alanine transaminase (ALT) and aspartate transaminase (AST) (Table 4). From Day 3 to the end of the study, 3/228 (1.3%) patients in the pyronaridine-artesunate group had peak ALT >5x the upper limit of normal (ULN); two (0.9%) with peak ALT >10×ULN. Total bilirubin values were within normal limits for both of these subjects throughout the study. No patients in the chloroquine group had ALT >5×ULN. Post-treatment AST was >5×ULN in one patient in the chloroquine group and >10×ULN in one patient from the pyronaridine-artesunate group; total bilirubin was within normal limits. Values for AST and ALT were close to or within normal limits by Day 28. No patient had peak ALT or AST >3×ULN plus total bilirubin >2×ULN during the study.

Clinically significant electrocardiograms were recorded for 1/226 (0.4%) patients in the pyronaridine-artesunate group and 6/222 (2.7%) in the chloroquine group. All cases were recorded as QTc prolongation. All except one event (in the chloroquine group) was considered unrelated to study treatment by the investigator.

## Discussion

Pyronaridine-artesunate was highly efficacious in the treatment of uncomplicated *P. vivax* malaria with a Day-14 cure rate of 99.5% that was non-inferior to that of chloroquine (100%). Non-inferiority of pyronaridine-artesunate to chloroquine was maintained throughout the study. However, primaquine treatment was initiated in most cases after Day 28, making comparisons after this time point difficult. There are no comparative data of pyronaridine-artesunate in *P. vivax* malaria. Results for chloroquine were comparable to other studies in Thailand and India (100% efficacy) [26], [27]. Data for Cambodia and the island of Flores in Indonesia are lacking, though studies in other regions of Indonesia indicate reduced efficacy of chloroquine against *P. vivax* [28], [29]. Although all 11 patients treated with chloroquine at Maumere, Indonesia, were cured at Day 14, the sample size was small and further studies in this region are required.

The clinical efficacy of the artemisinin derivatives is characterized by an almost immediate onset of activity and rapid reduction of parasitemia [30]. Thus, the shorter times to parasite clearance and fever clearance seen with pyronaridine-artesunate versus chloroquine in this study were expected. Studies of artesunate monotherapy in *P. vivax* malaria have also demonstrated rapid clearance of parasites and fever [15], [31], [32]. Parasite clearance was faster with pyronaridine-artesunate than chloroquine in all study centers. There was a suggestion of longer median parasite clearance times for both treatments at the Maumere center (Table 2). However, the small number of patients from this center compared with the other centers suggests caution in interpreting these results. As we did not collect susceptibility data or perform molecular analysis of isolates in this study, further investigation of these data is not possible.

The incidence of *P. vivax* or *P. falciparum* post-baseline infection was lower with pyronaridine-artesunate versus chloroquine, mainly because of the difference in *P. falciparum* post-baseline infections. Post-baseline infection with *P. falciparum* was less frequent and occurred later in the study with pyronaridine-artesunate versus chloroquine. Given the potential severity of *P. falciparum* malaria, this difference is clinically important. *P. falciparum* chloroquine resistance is widespread [33], and known to be present in the regions included in this study [12], [34]–[37]. Pyronaridine has potent *in vitro* activity against chloroquine-resistant *P. falciparum* [20] and previous Phase II and III pyronaridine-artesunate studies showed high (>99%) clinical efficacy against *P. falciparum* [23], [24]. We believe that chloroquine resistance may explain the higher rate of *P. falciparum* infections in the chloroquine group. However, we cannot confirm this as susceptibility or molecular testing of isolates was not performed.

Adverse events of any cause with pyronaridine-artesunate in this study were consistent with reports for pyronaridine and artemisinins as monotherapy in falciparum malaria [38]–[43], and previous clinical studies of fixed-dose pyronaridine-artesunate in falciparum malaria [23], [24]. Although drug-related adverse events can be difficult to interpret in malaria, they are included here for completeness and are comparable with previous studies [23], [24]. The adverse event profile for chloroquine was similar to that reported previously [41]. By proportion, there were marginally more adverse events of any cause in the pyronaridine–artesunate group than in the chloroquine group (Table 3). However, the only notable difference in individual adverse events was a greater incidence of increased transaminases with pyronaridine-artesunate versus chloroquine. This was supported by laboratory findings of transient increases in ALT and AST in the pyronaridine-artesunate group. However, there were no patients with ALT and/or AST >3×ULN and concomitant total bilirubin >2×ULN. This is in contrast to the previously reported pyronaridine-artesunate Phase III study in *P. falciparum* malaria [24], in which 2/849 (0.2%) patients had ALT and/or AST >3×ULN plus total bilirubin >2×ULN, though with no increase in ALP; there were no cases in the comparator regimen. These data were reviewed by an Independent Data Monitoring Committee (IDMC) comprising six members, including three experts in drug-induced liver injury. The IDMC concluded that pyronaridine-artesunate for three days can cause transient ALT elevations in a small subset of patients. However, the early onset (Day 3–7) and rapid resolution were all consistent with a direct low-level toxicity. These observations, combined with the fact that pyronaridine-artesunate is only administered for three days suggest that there is a very low risk of liver injury with pyronaridine-artesunate.

Mixed *P. vivax* and *P. falciparum* infection and *P. falciparum* reinfection are common in areas endemic for *P. vivax*. Practical difficulties in reliably distinguishing between the two pathogens means that, in practice, many malaria cases will be diagnosed clinically and treated empirically [12], [18]. In our study, patients with microscopically detected baseline mixed *P. vivax*/*P. falciparum* infection were excluded. However, our design does reflect the clinical situation in the study region where known *P. falciparum* malaria would not be treated with chloroquine because of the high risk of failure from widespread resistance to this agent [12]. A trial designed to provide data more relevant to the clinical situation would include *P. vivax* and mixed *P. vivax*/*P. falciparum* infection and would compare pyronaridine-artesunate to another ACT.

In this Phase III trial, the study population was restricted in order to allow comparison of the two treatments and probably does not reflect the baseline clinical and demographic characteristics of the general population of malaria patients in the region. Further studies are required to assess pyronaridine-artesunate in the wider clinical setting. Further data are also required to assess pyronaridine-artesunate efficacy in areas of high *P. vivax* chloroquine resistance. *In vitro* data indicate high activity for pyronaridine against chloroquine-resistant *P. vivax* [20], and a Phase IIIb/IV trial is planned to investigate this clinically in Papua New Guinea.

Our study included only 27 children, all over 7 years of age (probably because of the 20 kg minimum weight requirement). Pyronaridine-artesunate efficacy and safety data are required in very young children with *P. vivax* malaria, particularly because this group is the most vulnerable to severe outcomes [7], [44]. The planned study in Papua New Guinea mentioned above will be conducted in young children using a pediatric granule formulation of pyronaridine-artesunate.

In conclusion, pyronaridine-artesunate had non-inferior efficacy and faster parasite and fever clearance compared with chloroquine against *P. vivax*. Post-baseline *P. falciparum* infection was reduced and delayed with pyronaridine-artesunate versus chloroquine. Based on the results of this study in *P. vivax* and those conducted against *P. falciparum* [23], [24], pyronaridine-artesunate is a promising antimalarial therapy for use in regions where these species are sympatric.

## Supporting Information

Checklist S1 CONSORT checklist.
(0.23 MB DOC)

Protocol S1 Trial Protocol.
(0.62 MB PDF)
