# Supplementary material for: Pyronaridine-Artesunate versus Chloroquine in Patients with Acute Plasmodium vivax Malaria: A Randomized, Double-Blind, Non-Inferiority Trial
Source: PLoS One. 2011 Jan 18;6(1):e14501. doi: 10.1371/journal.pone.0014501 (PMC3022577; doi:10.1371/journal.pone.0014501)
Supplement: Protocol S1 — Trial Protocol. (0.62 MB PDF) [file pone.0014501.s002.pdf]

CONFIDENTIAL

Amendment 2 (administrative amendment)
Date: August 9th 2007

Clinical Study Protocol

A Phase III multi-centre, randomised, double-blind, double-dummy, comparative clinical study to assess the safety and efficacy of a fixed-dose formulation of oral pyronaridine artesunate (180:60 mg tablet) versus chloroquine (155 mg tablet), in children and adult patients with acute *Plasmodium vivax* malaria

Protocol number: SP-C-006-06

Protocol date: August 28th 2006
Protocol version number: Final 1.0
Amendment 1 date: March 5th 2007
Protocol version number: Final 2.0

Sponsors: Medicines for Malaria Venture, Switzerland
Shin Poong Pharmaceuticals, Republic of Korea

1. PURPOSE OF THE AMENDMENT AND RATIONALE FOR THE CHANGES

The purpose of the present administrative amendment to protocol SP-C-006-06 version 2.0, dated 5 March 2007 is to:

1.1. increase the maximum number of patients per site from 100 to approximately 160 without modifying the total number of patients required in the study, as the total number of participating sites is lower than anticipated.

1.2. increase study timelines due to a longer start up time of the study; the accrual is now anticipated to close around the end of Q3 2007 or the beginning of Q4 07.

2. CHANGES TO THE TEXT

The following corrections are to be incorporated into the study protocol:

Study Synopsis – Number of Patients and Randomisation, page 13

Formerly read:

A total of 456 patients from approximately 6 to 7 study sites will be randomised, with a maximum of 100 patients to be included per site.
Patients will be randomly assigned in a 1:1 ratio to one of the following treatment arms:
pyronaridine artesunate (180:60 mg tablets) + chloroquine-placebo
or
chloroquine (155 mg tablets) + pyronaridine artesunate-placebo

Now reads:

A total of 456 patients from approximately **4** to **6** study sites will be randomised, with a maximum of **approximately 160** patients to be included per site.
Patients will be randomly assigned in a 1:1 ratio to one of the following treatment arms:
pyronaridine artesunate (180:60 mg tablets) + chloroquine-placebo
or
chloroquine (155 mg tablets) + pyronaridine artesunate-placebo

Section 3.2 Selection of Study Population, page 28

Formerly read:

This study will be conducted in a total of 456 male and female children (≥ 20 kg body weight) and adult patients suffering from acute symptomatic uncomplicated *P. vivax* malaria recruited from trial sites located in South East Asia and India. The study will be randomised, with a maximum of 100 patients to be included per site.
Patients will be randomised in a 1:1 ratio to one of the following two treatment arms:

- Active-pyronaridine artesunate plus chloroquine-placebo
- Active-chloroquine plus pyronaridine artesunate-placebo

Now reads:

This study will be conducted in a total of 456 male and female children (≥ 20 kg body weight) and adult patients suffering from acute symptomatic uncomplicated *P. vivax* malaria recruited from trial sites located in South East Asia and India. The study will be randomised, with a maximum of **approximately 160** patients to be included per site.
Patients will be randomised in a 1:1 ratio to one of the following two treatment arms:

- Active-pyronaridine artesunate plus chloroquine-placebo
- Active-chloroquine plus pyronaridine artesunate-placebo

<u>Section 7.11 Study Timetable and Termination, page 69</u>

Formerly read:

The planned start date for this study is Q4 2006. It is anticipated that accrual will take up to approximately 5 months per site and the proposed patient completion date is estimated as Q2 2007

Now reads:

The planned start date for this study is Q4 2006. It is anticipated that accrual will take up to approximately 10 months per site and the proposed patient completion date is estimated as **the end of Q3 2007 or the beginning of Q4 07.**

CONFIDENTIAL

Clinical Study Protocol

A Phase III multi-centre, randomised, double-blind, double-dummy, comparative clinical study to assess the safety and efficacy of a fixed-dose formulation of oral pyronaridine artesunate (180:60 mg tablet) versus chloroquine (155 mg tablet), in children and adult patients with acute *Plasmodium vivax* malaria

Protocol number: SP-C-006-06
Protocol date: August 28th 2006
Amendment 1 date: March 5th 2007
Protocol version number: Final 2.0

Sponsors: Medicines for Malaria Venture, Switzerland
Shin Poong Pharmaceuticals,
Republic of Korea

Protocol Approval and Authorisation

Study Medical Monitor

Name	Affiliation	Signature	Date
**Robert Miller, FRCS**<br>**Chief Medical Officer**	**Fulcrum Pharma Developments**<br>Hemel One<br>Boundary Way<br>Hemel Hempstead<br>Hertfordshire HP2 7YU<br>United Kingdom		

Statistician

Name	Affiliation	Signature	Date
**Abdallah Ennaji, MSc**<br>**Vice President, Head of Data Management and Statistics**	**Hesperion AG**<br>Gewerbestrasse 24<br>CH-4123 Allschwil<br>Switzerland		

Sponsor Representatives

Name	Affiliation	Signature	Date
**J Carl Craft, MD**<br>**Chief Scientific Officer**	**Medicines for Malaria Venture (MMV)**<br>ICC, Route de Pre-Bois 20,<br>PO Box 1826, 1215<br>Geneva 15, Switzerland		

Name	Affiliation	Signature	Date
**Chang-Sik Shin, PhD**<br>**Director, R&D**	**Shin Poong Pharm Co., Ltd**<br>748-31 Yoksam-Dong,<br>Kangnam-Ku<br>Seoul, Korea		

ADMINISTRATIVE STRUCTURE

Clinical Study Support Structure and Responsibilities

Responsibility	Name and Affiliation
**Drug Blood Levels Analysis**	**Prof Larry Fleckenstein**<br>College of Pharmacy, S-427 PHAR<br>115 South Grand Avenue<br>University of Iowa<br>Iowa City, IA 52242<br>United States of America<br>Telephone: +1-319-335-8804<br>Fax: +1-319-353-5646<br>Email: l-fleckenstein@uiowa.edu
**Project Director**	**Dr Sarah Arbe-Barnes**<br>Fulcrum Pharma Developments<br>Hemel One<br>Boundary Way<br>Hemel Hempstead<br>Hertfordshire HP2 7YU<br>United Kingdom<br>Telephone: +44 870 710 7151<br>Fax: +44 870 710 7151<br>Email: sarah.arbe-barnes@fulcrumpharma.com
**Clinical Trial Site Management and Monitoring**	**Dr Steve Wignall**<br>FHI Asia Pacific Department<br>Sindhorn Building Tower 3 19th Floor<br>130-132 Wireless Rd.<br>Lumpini, Phatumwan<br>Bangkok 10330<br>Thailand<br>Telephone: +662 263 2300<br>Fax: +662 263 2114<br>Email: swignall@fhibkk.org
**Study Medical Monitor**	**Dr Robert Miller**<br>Fulcrum Pharma Developments<br>Hemel One<br>Boundary Way<br>Hemel Hempstead<br>Hertfordshire HP2 7YU<br>United Kingdom<br>Telephone: +44 870 710 4360<br>Fax: +44 870 710 4360<br>Email: robert.miller@fulcrumpharma.com

**Pharmacovigilance**	**Maria Hilling**<br>Manager of Pharmacovigilance Operations<br>Akos Limited<br>The Coach House, The Grove, Pipers Lane, Harpenden, Herts, AL5 1AH, England<br>Telephone: +44 (0) 1582 766339<br>Fax: +44 (0) 1582 764327<br>Email: maria.hilling@akos.co.uk
**Data Management and Biostatistics**	**Abdallah Ennaji**<br>Hesperion AG<br>Gewerbestrasse 24<br>CH-4123 Allschwil<br>Switzerland<br>Telephone: +41 61 487 16 48<br>Fax: +41 61 487 14 01<br>Email: abdallah.ennaji@hesperion.com
**Quality Assurance**	**Sponsors or Designees**

Sponsor Contact Details

**Chang-Sik Shin, PhD**<br>Director R&D	Shin Poong Pharm Co., Ltd<br>748-31 Yoksam-Dong, Kangnam-Ku<br>Seoul, Korea<br><br>Telephone: +82-2-2189-3499<br>Mobile: +82-11-895-5982<br>Fax: +82 2 553 2578<br>Email: casishin@shinpoong.co.kr
**J Carl Craft, MD**<br>Chief Scientific Officer	Medicines for Malaria Venture (MMV),<br>ICC,<br>Route de Pre-Bois 20,<br>PO Box 1826, 1215<br>Geneva 15, Switzerland<br><br>Telephone: +41 22 799 40 67<br>Mobile: +41 79 605 79 89<br>Fax: +41 22 799 40 61<br>Email: craftjc@mmv.org

Table of Contents

ADMINISTRATIVE STRUCTURE 3

LIST OF ABBREVIATIONS AND DEFINITION OF TERMS 10

DEFINITIONS 12

SYNOPSIS 13

1. INTRODUCTION 18

2. STUDY OBJECTIVES 27

3. METHODS AND INVESTIGATIONAL PLAN 28

3.1. Study Design Overview 28

3.2. Selection of Study Population 28

3.2.1. Inclusion criteria 28

3.2.2. Exclusion criteria 29

3.3. Patient Discontinuation or Protocol Violation 30

4. STUDY DRUG ADMINISTRATION PROCEDURES 32

4.1. Patient Assignment and Blinded Administration 32

4.2. Materials and Supplies 32

4.2.1. Study drug: pyronaridine artesunate or matching placebo 32

4.2.2. Chloroquine or matching placebo 33

4.2.3. Primaquine 35

4.3. Sponsor's Responsibilities 36

4.3.1. Study drug: pyronaridine and artesunate 36

4.3.2. Comparator drug: chloroquine 37

4.3.3. Primaquine 37

4.4. Storage Conditions 37

4.5. Drug Administration 37

4.6. Emergency Unblinding 38

4.7. Compliance....39

4.8. Study Site's Responsibilities....39

4.9. Clinical Study Material Accountabillity....39

4.10. Concomitant and Forbidden Medication....40

4.11. Rescue Medication....40

5. ASSESSMENTS....41

5.1. Efficacy Assessments....41

5.1.1. Parasitological assessments....41

5.1.1.1. Microscopy....41

5.1.1.2. Genotyping studies by PCR....43

5.1.2. Clinical efficacy assessments....43

5.1.2.1. Body temperature....43

5.1.2.2. Clinical signs and symptoms of malaria....44

5.1.3. Drug levels at time of treatment failure....44

5.1.4. Population pharmacokinetics....44

5.2. Safety Assessments....44

5.2.1. Clinical safety assessments....45

5.2.1.1. Physical examination....45

5.2.1.2. Body weight and height....45

5.2.1.3. Vital signs....45

5.2.2. Laboratory assessments....46

5.2.3. ECG....48

5.2.4. Adverse events....49

5.2.4.1. Adverse event definition....49

5.2.4.2. Assessment of adverse events....51

5.2.4.3. Adverse event reporting requirements....53

5.2.4.4. Serious adverse event reporting procedures....53

5.2.4.5. Safety monitoring plan....55

5.2.4.6. Sponsor emergency contact procedure....55

5.2.4.7. Procedures in case of medical emergency....56
5.2.4.8. Procedures in case of overdose....56
5.2.4.9. Emergency procedures....56
5.2.4.10. Follow-up of patients....56

6. SCHEDULE OF STUDY PROCEDURES....57

7. STUDY ADMINISTRATION....63
7.1. Monitoring....63
7.2. Special Consideration of Safety Monitoring....64
7.2.1. Medical monitor responsibilities....64
7.2.2. Sponsor responsibilities....64
7.3. Audits and Inspections....64
7.4. Training of Staff....64
7.5 Changes to the Protocol....65
7.6. Data Handling and Record Keeping....65
7.7. Patient Confidentiality....67
7.8. Direct Access to Source Data/Documents....67
7.9. Investigator Information....68
7.9.1. Protocol approval and signature....68
7.9.2. Study agreements....68
7.9.3. Publication policy....68
7.10 Financing and Insurance....68
7.11. Study Timetable and Termination....69

8. DATA MANAGEMENT....70

9. STATISTICAL METHODS....71
9.1. General Considerations....71
9.2. Analysis Populations....71
9.3. Patient Disposition....72
9.4. Determination of Sample Size....72

9.5. Statistical Evaluation 73

9.5.1. Efficacy and safety endpoints 73

9.5.2. Methods of statistical analysis 75

9.5.3. Interim analyses 78

10. ETHICS 79

10.1. Ethics Review 79

10.2. Ethical Conduct of the Study 79

10.3. Patient Information and Consent 79

10.4. Patient Data Protection 81

11. REFERENCES 82

ATTACHMENT 1 PROTOCOL APPROVAL AND SIGNATURE PAGE...86

ATTACHMENT 2 STUDY PLAN 87

ATTACHMENT 3 CLASSIFICATION OF TREATMENT OUTCOMES.....88

ATTACHMENT 4 DOSING REGIMENS 89

ATTACHMENT 5 FORBIDDEN MEDICATIONS 90

ATTACHMENT 6 DRUG CONCENTRATION SPECIMEN COLLECTION, PROCESSING, STORAGE AND SHIPPING 91

ATTACHMENT 7 DIVISION OF MICROBIOLOGY AND INFECTIOUS DISEASES (DMID) TOXICITY GRADING SCALES FOR DETERMINING SEVERITY OF ADVERSE EVENTS 93

ATTACHMENT 8 DECLARATION OF HELSINKI 105

ATTACHMENT 9 ELEMENTS OF PATIENT INFORMED CONSENT ...109

ATTACHMENT 10 ETHICAL COMMITTEE REVIEW FORM 111

LIST OF ABBREVIATIONS AND DEFINITION OF TERMS

Abbreviation	***Term***
ACPR	Adequate Clinical and Parasitological Response
ACT	Artemisinin-based Combination Therapy
AE	Adverse Event
AIDS	Acquired Immunodeficiency Syndrome
ALT /SGPT	Alanine Aminotransferase
ALP	Alkaline Phosphatase
AS	Artesunate
AST/SGOT	Aspartate Aminotransferase
BP	Blood Pressure
BUN	Blood Urea Nitrogen
°C	Degree Celcius
CBC	Complete Blood Count
Cmax	Maximum Peak Observed Concentration
CRA	Clinical Research Associate
CRF	Case Report Form
CV	Curriculum Vitae
DCF	Data Clarification Form
DHA	Dihydroartemisinin
DMID	Division of Microbiology and Infectious Disease
EC	Ethics Committee
ECG	Electrocardiogram
FCT	Fever Clearance Time
FEV	Forced Expiratory Volume
FDA	Food and Drug Administration
GCP	Good Clinical Practice
GGT	Gamma-Glutamyl-Transferase
GMP	Good Manufacturing Practice
G-6-PD	Glucose-6-Phosphate Dehydrogenase
Hb	Hemoglobin
HCG	Human Chorionic Gonadotropin
HIV	Human Immunodeficiency Virus
HR	Heart Rate
ICH	International Conference on Harmonisation
IC50	Inhibitory Concentration in 50% of cases
IEC	Independent Ethics Committee
IgM	Immunoglobulin M
ITT	Intention to Treat
IV	Intravenous
Kg	Kilogram
L	Litre
LDH	Lactate Dehydrogenase
MMV	Medicines for Malaria Venture
NCR	No Carbon Required
NOEL	No Observed Effect Level
NOAEL	No Observed Adverse Effect Level
PA	Pyronaridine Artesunate (combined)
PCR	Polymerase Chain Reaction
PCT	Parasite Clearance Time
PD	Pharmacodynamic
P	Plasmodium
PK	Pharmacokinetic
PI	Principal Investigator
PP	Pyronaridine tetraphosphate

Abbreviation	*Term*
PT	Prothrombin Time
RBC	Red Blood Cell
Rx	Treatment
SAE	Serious Adverse Event
SAP	Statistical Analysis Plan
S/P	Sulfadoxine/Pyrimethamine
STI	Swiss Tropical Institute
t½	Half-life of elimination
ULN	Upper Limit of Normal
WBC	White Blood Cell
WHO	World Health Organization
w/w	Weight/weight

Definitions

Parasite clearance: at least two consecutive negative smears for parasites obtained within an interval of 8 to 24 hours, post-dosing.

Fever clearance: at least two consecutive normal body temperature measurements obtained within an interval of 8 to 24 hours, post-dosing.

Treatment failure: the definition of treatment failure includes the following:

- Clinical deterioration due to P. vivax illness requiring hospitalization in presence of parasitaemia.
- Presence of parasitemia and axillary temperature ≥37.5°C any time between Day 3 and Day 28.
- Presence of parasitemia on any day between Day 7 and Day 28 irrespective of clinical condition.

Cure on Day 14: no parasitaemia on Day 14 irrespective of axillary temperature without previously meeting any of the criteria of treatment failure.

Cure on Day 28: no parasitaemia on Day 28 irrespective of axillary temperature without previously meeting any of the criteria of treatment failure.

Synopsis

Study Title	A Phase III multi-centre, randomised, double-blind, double-dummy, comparative clinical study to assess the safety and efficacy of a fixed-dose formulation of oral pyronaridine artesunate (180:60 mg tablet) versus chloroquine (155 mg tablet), in children and adult patients with acute *Plasmodium vivax* malaria
Regulatory Status	Investigational – Phase III.
Investigational Product and Route	Pyronaridine tetraphosphate artesunate (PA) 180:60 mg single combined tablet for oral administration.
Study Objective	The primary objective of this clinical study is to compare the efficacy and safety of the fixed combination of pyronaridine artesunate (180:60 mg) with that of standard chloroquine therapy in children and adults with acute, uncomplicated *Plasmodium vivax* (*P. vivax*) malaria.
Study Site	Approximately six to seven study sites will participate in the study.
Country Involvement	To include: Cambodia, India, Indonesia and Thailand.
Number of Patients and Randomisation	A total of 456 patients from approximately 6 to 7 study sites will be randomised, with a maximum of 100 patients to be included per site.<br>Patients will be randomly assigned in a 1:1 ratio to one of the following treatment arms:<br>pyronaridine artesunate (180:60 mg tablets) + chloroquine-placebo<br>or<br>chloroquine (155 mg tablets) + pyronaridine artesunate-placebo
Trial Design	The trial is designed as a multicentre, randomised, double-blind, double-dummy, comparative study of the efficacy and safety of a three day regimen of the fixed combination of pyronaridine artesunate (3:1) versus chloroquine.<br>Patients will be followed for safety for 42 days after the first study drug administration. The primary efficacy endpoint will be at Day 14.<br><br>Except for patients who are deficient in glucose-6-phosphate dehydrogenase (G-6-PD), patients completing the study up to Day 28 shall be given a 14-day course of primaquine (15 mg/day) starting on Day 28, after all required assessments have been completed (including demonstration of a negative pregnancy test for women of childbearing potential). Patients who are deficient in G-6-PD and who complete Day 28 will be treated as per country policy. All patients will have a final assessment on Day 42.
Study Drugs & Comparator	**Pyronaridine artesunate (or matching placebo):**<br>The tablet strength to be used is 180:60 mg pyronaridine artesunate. Depending on their body weight, patients will be administered 1 to 4 tablets once a day, for 3 days.<br>The actual dose-level range covered by this regimen is 7.2: 2.4 mg/kg to 13.8:4.6 mg/kg pyronaridine artesunate, which has been shown to be effective and safe in the phase II studies conducted in healthy volunteers and patients.<br><br>**Chloroquine (or matching placebo):**<br>The tablet strength to be used is 155 mg chloroquine base administered

	orally. Patients will receive:<br>For adults: 620 mg (i.e. 4 tablets) on Days 0 and 1 and 310 mg (i.e. 2 tablets) on Day 2.<br>For children: 10 mg/kg on Days 0 and 1 and 5 mg/kg on Day 2.
Duration of Patient Participation	In total, patient participation is for 42 days following inclusion in the trial.<br><br>Patients will be confined to the study facility for at least the first 4 days (Study Day 0, 1, 2 and 3) and will ideally remain in the vicinity of the trial site for a minimum of 7 days or when fever and parasite has been cleared for at least 24 hours, whichever is the later. The patient is to return to the study site for all scheduled follow up visits until completion of the study on Day 42.<br>In the case of adverse events reported and unresolved at visit Day 42, patients will be followed up for a further 30 days, or until resolution of the event, whichever is the earlier. Serious adverse events will be followed up to resolution or until no new medical information can be expected.
Inclusion Criteria	Patients must fulfil the following inclusion criteria to be eligible for enrolment in the study:<br>1. Male or female patients between the age of 3 and 60 years, inclusive.<br>2. Body weight between 20 kg and 90 kg with no clinical evidence of severe malnutrition.<br>3. Presence of acute uncomplicated *P. vivax* mono-infection confirmed by:<br>a. Fever, as defined by axillary/tympanic temperature ≥37.5°C or oral/rectal temperature ≥38°C, or history of fever in the previous 24 hours (history of fever must be documented) **and,**<br>b. Positive microscopy of *P. vivax* with parasite density ≥250/ µL of blood (including at least 50% of asexual parasites)<br>4. Written informed consent, in accordance with local practice, provided by patient and/or parent/guardian/spouse. If the patient is unable to write, witnessed consent is permitted according to local ethical considerations.<br>5. Ability to swallow oral medication.<br>6. Ability and willingness to participate based on information given to patient or parent or guardian and access to health facility.
Exclusion Criteria	To be eligible for enrolment in the study, patients must NOT meet any of the following criteria:<br>1. Presence of a mixed *Plasmodium* infection.<br>2. Presence of other clinical condition requiring hospitalization.<br>3. Presence of significant anaemia, as defined by Hb < 8 g/dL.<br>4. Known history or evidence of clinically significant disorders such as cardiovascular (including arrhythmia, QTc interval greater than or equal to 450 msec), respiratory (including active tuberculosis), hepatic, renal, gastrointestinal, immunological (including active HIV-AIDS), neurological (including auditory), endocrine, infectious, malignancy, psychiatric or other abnormality (including recent head trauma).<br>5. Known history of hypersensitivity, allergic or adverse reactions to pyronaridine, chloroquine or artesunate or other artemisinins.<br>6. Known history of hypersensitivity, allergic or adverse reactions to chloroquine, primaquine and related agents.<br>7. Known active Hepatitis A IgM (HAV-IgM), Hepatitis B surface antigen (HBsAg) or Hepatitis C antibody (HCV Ab).

	8. Known seropositive HIV antibody.<br>9. Have received any antimalarial treatment in the preceding 2 weeks, as determined by history and, whenever feasible, by screening test.<br>10. Have received antibacterial with known antimalarial activity in the preceding 2 weeks.<br>11. Have received any investigational drug within the past 4 weeks.<br>12. Liver function tests (AST/ALT levels) more than 2.5 times the upper limit of normal range.<br>13. Known significant renal impairment as indicated by serum creatinine levels of more than 1.4 mg/dL.<br>14. Female patients of child-bearing potential must be neither pregnant (as demonstrated by a negative pregnancy test) nor lactating, and must be willing to take measures to not become pregnant during the study period.<br>15. Previous participation in the present clinical trial with pyronaridine artesunate.
Concomitant Treatment	The administration of paracetamol/acetaminophen (≤ 1 g) on Day 0, 1 and 2 is allowed if the patient's condition warrants it and should be recorded in the appropriate section of the CRF.<br>Rescue treatment: Treatment failures will be treated with best antimalarial therapy available in the country where the study is conducted.<br>If during follow-up, infections other than malaria require the administration of drugs with antimalarial activity (such as co-trimoxazole, tetracycline or doxycycline), the patient will be considered a protocol violator, with the exception of patients given tetracycline as eye ointment.<br>All concomitant medications taken by the patient during the study, from the date of signature of the informed consent will be recorded in the appropriate section of the CRF.
Study Drugs Administration	Study drug will be administered orally once a day for three consecutive days, i.e. on Days 0, 1 and 2, by authorized member of the investigation team.<br>Depending on their body weight, patients will receive a total of between 2 to a maximum of 8 tablets per day on Days 0 and 1 and a total of between 1.5 to a maximum of 6 tablets per day on Day 2.
Endpoints	Primary efficacy endpoint:<br>♦ Cure rate on Day 14.<br><br>Secondary efficacy endpoints:<br>♦ Cure rate on Day 21 and 28.<br>♦ Parasite Clearance Time (PCT).<br>♦ Fever Clearance Time (FCT).<br>♦ Proportion of patients who have cleared parasites on days 1, 2, and 3.<br>♦ Proportion of patients who have cleared fever on days 1, 2, and 3.<br><br>Safety endpoints:<br>♦ Incidence of adverse events and of clinically significant laboratory results, ECG, vital signs or physical examination abnormalities.<br><br>Exploratory endpoints:<br>♦ Proportion of patients with PCR-corrected cure on Day 14.<br>♦ Proportion of patients with PCR-corrected cure on Day 28.<br>♦ Cure rate on Day 42.<br>♦ Proportion of patients with PCR-corrected cure on Day 42.

	Additional Assessments:<br>Characterization of pyronaridine and artesunate kinetics will be conducted using a population pharmacokinetic approach.
Clinical and Laboratory Assessments	Baseline examination:<br>♦ Demographic data, medical history and history of medications.<br>♦ Physical examination, body weight and height, vital signs, and body temperature.<br>♦ Clinical signs and symptoms of malaria<br>♦ 12-lead ECG.<br>♦ Clinical safety laboratory evaluations: haematology, biochemistry and urinalysis (dipstick).<br>♦ Urine pregnancy test in female patients of child-bearing potential.<br>♦ Urine analysis for previous anti-malarial drugs, if feasible.<br>♦ Parasitological examination: thick and thin blood films for parasite species identification and count. Both asexual parasite and gametocyte counts will be recorded at screening.<br>♦ Screening for G-6-PD deficiency.<br>Routine assessments:<br>♦ Clinical<br>o Physical examination (on Days 0, 3, 28 and 42).<br>o Recording of adverse events and concomitant medications.<br>o Vital signs: on Days 0,1, 2, 3, 7, 14, 21, 28, 35 and 42.<br>o Body temperature: on Day 0 (Pre-dose) and every 8 hours for at least 72 hours or when fever has normalised (<37.5°C axillary/tympanic or <38.0°C oral/rectal): 2 normal readings taken between 8 and 24 hours apart. Also on Days 3, 7, 14, 21, 28, 35 and 42.<br>o 12-lead ECG: on Day 0 (Pre-dose) and on Day 2, 2-4h post-dose and Day 28. Repeated, if clinically indicated, on Days 7, 14 and 42.<br>o Clinical signs and symptoms of malaria on Day 0, 1, 2, 3 and any other day with fever.<br><br>♦ Laboratory tests:<br>o Haematology on Days 0, 3, 7, 28 and 42. Repeated if clinically indicated on other visits.<br>o Blood biochemistry and urinalysis (by dipstick) on Days 0, 3, 7 and 28. Repeated if clinically indicated on Day 42.<br>o Urine hCG test for pregnancy for female patients on Days 0, 14, 28 and 42.<br>♦ Parasitological examination:<br>o Thick and thin blood films for parasite count should be obtained and examined at screening on Day 0 to confirm inclusion/exclusion criteria. Thick blood films will be examined every 8 hours until at least 72 hours or until two consecutive negative readings are obtained not less than 8 and not more than 24 hours apart. Asexual parasite and gametocyte counts will both be recorded at screening, while total parasite counts will be recorded thereafter.<br>o Thick blood films will be also examined on Days 3, 7, 14, 21, 28, 35 and 42 or on any other day if the patient spontaneously returns. Additionally, blood films should be obtained whenever parasitological reassessment is

	required.<br>o A thin blood smear will also be taken from Day 7 whenever parasitological blood samples are taken, and reserved in the event of recrudescence, to confirm *Plasmodium* species.<br>♦ PCR:<br>o Samples will be taken for PCR prior to Day 0 dosing and immediately prior to Day 1 dosing. PCR filter paper samples will also be taken at Days 7, 14, 21, 28, 35 and 42. The PCR analysis will, however, only be performed in the case of positive microscopy for asexual parasites. This analysis will be centralized at the Swiss Tropical Institute in Basel.
Drug Blood Levels	At selected clinical study sites (as determined by the Sponsor), for each patient, two blood samples will be drawn for measurement of pyronaridine and two blood samples will be drawn for measurement of artesunate concentration. The blood samples will be taken at different timepoints within specific pre-defined time windows. The exact time of blood sampling must be recorded.<br>Additionally, at all sites, a blood sample must be drawn for assay of pyronaridine (in whole blood) at time of treatment failure.
Statistical Analysis & Sample Size Justification	The primary efficacy analysis will test the non-inferiority of the pyronaridine artesunate (PA) group compared to the chloroquine group with regard to cure rate at Day 14 using two sided 95% confidence interval and a 10% non-inferiority margin and be performed on the efficacy evaluable (EE) population. Non-inferiority will be demonstrated if the lower limit of the two-sided 95% confidence interval (CI) for the difference in cure rates at Day 14 (normal approximation) is not lower than -10%.<br><br>Assuming a cure rate on Day 14 of 90% in both treatment groups and assuming a non-inferiority limit of -10%, then a sample size of 410 evaluable patients randomized in 1:1 ratio (205 patients in each treatment group) would provide 90% power to demonstrate non-inferiority of PA compared to chloroquine, using a two-sided 95% confidence interval with Normal approximation. Assuming a drop out rate of 10%, 456 subjects would be randomized to the study (228 subjects in each treatment group).

1. INTRODUCTION

Background

Plasmodium vivax malaria

Plasmodium vivax (*P. vivax*) represents a major health problem throughout the tropics. Outside of Africa, it accounts for over 50% of malaria cases, affecting an estimate of 70-80 million people per year, notably in South-East Asia and Central- and South-America and has a particularly strong impact on the archipelago of Indonesia. In addition, it is estimated that 10-20% of the *P. vivax* cases occur in Eastern and Southern Africa, while *P. vivax* cases are extremely rare in the countries of sub-Saharan West Africa. This is apparently due to the high prevalence of the Duffy negative trait in West Africans, a phenotype that lacks the receptor for invasion of the human red blood cell by *P. vivax* merozoites (Mendis et al, 2001). Furthermore, in recent years the re-emergence of *P. vivax* has become a major problem in malaria-endemic areas, such as Korea or China, where the disease had been eradicated many years ago (Sleigh et al, 1998; Chai et al, 1999; Oh et al, 2001).

The *P. vivax* infection is rarely life-threatening, but it is responsible for an important morbidity in all age groups (Karunaweera et al, 2003). Indeed*, P. vivax* forms persistent hypnozoite parasite stages in the liver that can result in multiple relapses of infection, weeks to months after the primary infection (Krotosky et al, 1982). Thus a single infection causes repeated bouts of illness that impact significantly the patients' health and ability to carry on activities of daily living. *P. vivax* causes a debilitating febrile illness with fevers as high as 39°-41°C. Other major symptoms include headache, myalgia, nausea, diarrhea and vomiting. In the majority of cases, *P. vivax* malaria is benign and vital organ dysfunction is very rare. Nevertheless, reports of cases of severe *P. vivax* malaria have been published and acute respiratory distress syndrome seems to be one of the more common complications (Kochar et al, 2005).

The blood schizontocide chloroquine and the tissue schizontocide primaquine have been the mainstay of *P. vivax* treatment in most areas of the world for the past 50 years. However, susceptibility of *P. vivax* to chloroquine has significantly decreased in various countries within the past two decades. Reports of chloroquine-resistance have emerged from several geographical regions,

including Papua New Guinea, Indonesia Irian Jaya, India, Myanmar, Guyana, Brazil, Peru and Colombia (Rieckmann et al, 1989, Shwartz et al, 1991; Baird, 1991; Kyaw et al, 1993; Murphy et al , 1993; Garg et al 1995; Marlar-Than et al, 1995; Baird et al, 1996; Philips et al, 1996; Dua et al 1996; Whitby, 1997; Villalobos-Salcedo et al, 2000; Soto et al, 2001; Sumawinata et al, 2003; Baird, 2004).

In the case of *Plamodium falciparum* (*P. falciparum)*, the combination Sulfadoxine / Pyrimethamine (S/P) is often the alternative chosen in the case of chloroquine-resistant parasites. S/P is not recommended for primary therapy of *P. vivax* malaria, however, due to its poor clinical efficacy (Doberstyn et al, 1979; Imwong et al, 2001; Pukrittayakamee et al, 2004).

Therefore, the evaluation of alternative therapies for the treatment of the blood stage of *P. vivax* is strongly needed. Furthermore, mixed infections with *P. falciparum* and *P. vivax* occur in some geographic locations, yet their frequency is difficult to estimate as they are usually underestimated when detected microscopically (Mayxay et al, 2004; Pukrittayakamee et al, 2004). Mixed-infections are reported to be more common when detected by the more sensitive technique PCR. Drugs that clear both species from the circulation are therefore needed.

Artesunate

Artemisinin derivatives are powerful blood schizontocidal anti-malarial agents with a history of clinical use both alone and in combination with other drugs, mainly to treat *P. falciparum* malaria (Woodrow et al 2005), but also for *P. vivax* malaria (Li et al, 1994; Li et al 1999; Phan et al 2002).

The clinical efficacy of the artemisinin derivatives is characterised by an almost immediate onset and rapid reduction of parasitaemia, with complete clearance in most cases within 48 hours (De Vries et al, 1996). To prevent the recrudescence observed with monotherapy, however, treatment for at least 5 days is required.

Despite extensive clinical and laboratory experience, their molecular target is not yet fully identified.

Of the available derivatives, artesunate has the most favourable pharmacological profile for use in the treatment of uncomplicated malaria. The presence of a hemisuccinate group in the molecule confers water solubility and relatively high

oral bioavailability. It is rapidly and quantitatively converted *in vivo* to the potent active metabolite dihydro-artemisinin.

Studies of artesunate monotherapy for *P. vivax* infection have demonstrated rapid clearance of fever and parasites (Batty et al, 1998; Pukrittayakamee et al 2000; Hamedi et al, 2004). Interestingly, a significant gametocidal activity of artesunate has also been reported. By reducing the transmission of *Plasmodium vivax* to the vector, artesunate could therefore reduce the incidence of *P. vivax* malaria (Nacher et al, 2004). However, 5-7 days monotherapy regimes are associated with recrudescence. This phenomenon highlights the limitations of monotherapy. To ensure high cure rates with short course 3-day therapy, combinations are recommended (Wilairatana et al, 1999; Tjitra et al, 2002; Silachamroon et al, 2003). Combination is also one method of overcoming drug-resistance and the use of combination antimalarial therapy, particularly artemisinin combination therapies (ACT), has been strongly recommended (WHO 2001).

Pyronaridine

Pyronaridine has been used in China for over 30 years and initial clinical trials conducted in this country have demonstrated its safety and efficacy by oral and parenteral routes against both *P. falciparum* and *P. vivax* (Fu and Xiao, 1991; Chen et al, 1992). These observations have been further extended and the drug has been shown to be satisfactorily tolerated and highly effective in treating malaria patients in other regions (Ringwald et al, 1996; Looareesuwan et al, 1996). Available data also indicate that pyronaridine is effective in cases of chloroquine resistance. In addition, evidence has been provided that the drug is also useful for treating *P. ovale* and *P. malariae* infections (Ringwald et al, 1997).

In addition to its effective short term effect, pyronaridine, with its intermediate half-life in the region of 7 days, provides a sustained schizontocidal effect.

The efficacy and toxicity of the combination of pyronaridine and primaquine have been studied in a number of animal models. Data obtained in a mouse model show that pyronaridine did not affect the tissue schizontocidal action of primaquine against *P. yoleii.* Similarly, it was demonstrated that pyronaridine did not affect the tissue schizontocidal action of primaquine in a monkey model using *P. cynomolgi* (Shao et al, 1988; 1990). Furthermore, studies performed in mice and rats indicate that the toxicity of pyronaridine combined with primaquine is lower than that of chloroquine in combination with primaquine.

The combination of oral pyronaridine (1.2 or 1.6 g divided in 3 to 4 doses) with primaquine has also been studies in a number of clinical trials in China (Hui et al, 1984; Liu et al 1990; Geng et al, 1991). All patients showed immediate clinical cure, while recrudescence rates were reported to be between 3.5 and 8.5%, 8 to 12 months after treatment. It was reported that patients tolerated the drug well and mild nausea and/or dizziness occurred only in very few cases.

Artesunate, pyronaridine and the treatment of *Plasmodium vivax* in children

Millions of doses of artemisinin drugs have been dispensed for the treatment of both *vivax* and *falciparum* malaria, and these compounds appear to be both safe and well-tolerated (Duffy et al, 2006). Available data suggest that both the artemisinin derivatives and pyronaridine are also safe and effective therapies for *P. vivax* in the paediatric population. Artesunate quickly cleared *vivax* parasites from the blood of children in Brazil and in Papua New Guinea (Alecrim et al, 2000; Karunajeewa et al, 2003). Artesunate quickly cleared both fever and parasitemia in 42 Thai patients aged 12-56 infected with *P. vivax* (Hamedi et al, 2004). The therapeutic response to artesunate was judged better than that of chloroquine. Both artesunate-mefloquine and dihydroartemisinin-piperaquine were highly efficacious and well-tolerated treatments for uncomplicated *falciparum* malaria in children aged 1-year or older in Burma (Smithuis et al, 2006). Several thousand cases of both *vivax* and *falciparum* malaria in China have been treated effectively and safely since 1971 in clinical trials of pyronaridine (Fu et al, 1991). Pyronaridine was well tolerated and safe when given to 41 African children with a mean age of 9.6 years (range 5-15) as treatment for *falciparum* malaria (Ringwald et al, 1998).

Fixed-dose combination of pyronaridine artesunate

The goal of anti-malarial drug development is to develop potent, safe, easy-to-administer and inexpensive combination therapies. Importantly, there is a need for new drugs that are efficacious against both *P. falciparum* and *P. vivax*, because in areas where both species exist and health systems are undersourced, it is often not possible to distinguish between the two species at the initial diagnosis. Given the data accumulated to date, it is anticipated that the combination of pyronaridine and artesunate, a new ACT, will fulfil these needs.

A large multicentre dose ranging study of fixed pyronaridine artesunate combination in acute uncomplicated malaria patients has been completed recently. The pyronaridine artesunate combination was shown to be highly effective and well tolerated in the dose range intended for the final product.

A single tablet strength containing 180:60 mg pyronaridine artesunate has been selected for development and licensing, to ease the administration, dosing regimen as well as the maintenance of inventory in low income countries.

Taken together, this forms the rationale for developing the fixed-dose combination pyronaridine artesunate (3:1) 3-day regimen for the treatment of blood stage *P. vivax* malaria, in parallel to its development in *P. falciparum* malaria.

Choice of comparator

In spite of the increasing numbers of resistant strains, chloroquine monotherapy is still recommended as standard blood-stage therapy for patients with *P. vivax* malaria in the countries in which this trial will be conducted. Its selection as comparator is therefore justified. The adult dose of chloroquine will be 620 mg for 2 days followed by 310 mg on the third day and for children 10 mg/kg for the first two days and 5 mg/kg for the third day. Total dose is in accordance with the current practice in the countries where the study is conducted. The safety profile of chloroquine is well established and known. Although generally well tolerated, the following side-effects of chloroquine treatment have been described:

Gastro-intestinal disturbances, headache, hypotension, convulsions, visual disturbances, depigmentation or loss of hair, skin reactions (rashes, pruritus) and, rarely, bone-marrow suppression and hypersensitivity reactions such as urticaria and angioedema. Their occurrence during the present trial may however be unlikely given the short (3-day) duration of treatment.

Non-clinical Summary

ICH-compliant pre-clinical studies have been conducted to GLP on pyronaridine, artesunate and the combination. The clinical study material has been produced to GMP standard by the Sponsor, Shin Poong Pharm, in conjunction with the Medicines for Malaria Venture (Pyronaridine Artesunate Investigator Brochure, Edition 3, March 2006).

Efficacy pharmacology

It has been shown that pyronaridine is active *in vitro* against *Plasmodia* species, laboratory and field isolates, both sensitive and resistant to other agents such as chloroquine with an IC_{50} of the order of 8 nmol/l. Artesunate, and its active metabolite DHA, are also active against a similar range of *Plasmodia* species with an IC_{50} values of the order of 4 and 1.5 nmol/l respectively.

Pyronaridine and artesunate alone inhibited *P.chabaudi* in mice at doses of 12 and 4 mg/kg respectively. A combination of a 3:1 ratio of the two drugs was equally active at 8 mg/kg (6 + 2 mg/kg).

Safety pharmacology

Pyronaridine had a mild peripheral analgesic activity and lowers body temperature at very high doses in rat. Artesunate showed an inhibition of barbiturate-induced sleeping times and a decrease in gastric acid secretion and urine volume, again, at high dose levels at and above 250 mg/kg. In combination there were no real changes in the pharmacological effects, other than those seen individually.

Pharmacokinetics

Both pyronaridine and artesunate are readily absorbed from the gastric mucosa. A steady state following repeated dosing is reached with pyronaridine in around seven days. There is dose-proportionality with a blood half-life of two to eight days. Pyronaridine is associated with erythrocytes in the circulation and is readily metabolised. Some 14 metabolites have been detected. Artesunate is rapidly converted to DHA (within minutes) in the circulation; the half-life is 25-60 minutes following oral administration. Artesunate circulates in the plasma compartment of the blood. Administration of the two drugs together appears to have little effect, if any, on the pharmacokinetics of either drug.

Toxicology

In GLP studies, pyronaridine was shown to produce hepatic, splenic and renal inflammatory changes in the rat with a NOEL of 12 mg/kg. In the dog the NOAEL was 15 mg/kg there was some incidence of reduction in erythrocyte indices and at high doses precipitation of yellow material in many tissues, presumed to be the drug substance or metabolites.

Artesunate induced a reduction in food consumption and body weight loss in rats and dogs. The NOEL in the rat was 30 mg/kg and the NOAEL 6 mg/kg in the dog. There was also a reduction in erythrocyte indices.

When administered together there was no evidence of the toxicity of either drug being enhanced.

Genotoxicity

Artesunate may be considered to be free from mutagenic or clastogenic potential.

Pyronaridine has shown positive genotoxic results in one study *in vitro*, however the *in vivo* studies showed no genotoxic effects up to doses which resulted in exposures of about 25 times the expected human C_{max} after the third dose. Studies of the genotoxicity of the combination of the two drugs provided no evidence of any effects other than those for pyronaridine alone.

Phase I Data with Pyronaridine-Artesunate Combination

A randomised, blinded phase I clinical study has been conducted with the fixed ratio combination of pyronaridine artesunate (3:1). The protocol combined multiple objectives of single and multiple dose-rising phases as well as food-effect and drug-interaction sub-studies. Four dose levels of pyronaridine artesunate were investigated in the single and multiple rising dose parts (6:2; 9:3; 12:4; 15:5 mg/kg pyronaridine artesunate, respectively). The study recruited healthy male and female subjects aged 19-40 years: 9 per group (7 active 2 placebo) in the single-rising dose; 10 per group (5 male, 5 female) in both the interaction and food effect studies; 8 per group (6 active 2 placebo) in the multiple dose-rising part. Dose escalation was approved by medical reviewers and Investigators on review of safety data. Assessments consisted of safety evaluation (vital signs, ECG, lab tests, adverse events), up to 14 days after dosing. With single and multiple dosing (once daily for 3 days), no individual clinically significant sustained drug-related changes were recorded in vital signs, ECGs and clinical laboratory tests. The adverse events at doses studied were minor or moderate in nature and resolved without sequelae. No serious or severe adverse events were reported. The adverse event profile was consistent those reported in the literature for both components and are predominantly gastrointestinal in nature. No subject discontinued treatment due to adverse events including vomiting. In summary, the combination was well-tolerated up to and including the dose ratio of 15:5 mg/kg,

with only mild or moderate adverse events, including gastrointestinal effects, observed. No significant food effect was reported and there was no notable drug interaction effect.

Moreover the pharmacokinetic profile of the combination therapy revealed that the artesunate component has a short elimination half-life and the pyronaridine component has an intermediate half-life. Blood pyronaridine and plasma artesunate exhibited approximate linear dose proportionality over the dose range: 6:2 to 15:5 mg/kg (pyronaridine:artesunate respectively) in the single dose study. Absorption was rapid, with a T_{max} of 1.6 to 4.8 h. for pyronaridine and 0.6 to 0.9 h. for artesunate. The elimination half-life ranged from 6.6 to 9.7 days for pyronaridine, 0.44 to 0.53 h. for artesunate, and 0.9 to 1.5 h. for DHA. Pharmacokinetic analyses show that blood pyronaridine concentrations accumulate with repeated daily doses, whereas plasma artesunate and DHA concentrations do not. Furthermore the food effect study does not suggest any clinically relevant effect of food on the bioavailability of either compound.

The data from the phase I study supported the transition for the combination product into adult patients with acute uncomplicated malaria. The pharmacokinetic profile after single and multiple doses regimens supports once-daily dosing.

Phase II Clinical Study in acute uncomplicated P. falciparum malaria

A phase II dose ranging study (SP-002-05) has been conducted in acute uncomplicated *P. falciparum* malaria patients between the ages of 15 and 60 years with doses of pyronaridine:artesunate of 6:2, 9:3 or 12:4 mg/kg. Patients were followed to 42 days post therapy, with a primary endpoint of 28 day PCR-corrected adequate clinical and parasitological response (ACPR). Four hundred and seventy seven patients were recruited in 8 sites in 6 countries in Africa and South-East Asia.

Parasitaemia cleared within approximately 30 hours at all dose levels. The cure rate at Day 28 was greater than 95% in all three dose levels.

Adverse events were as anticipated with the class of drugs, including those related to clinically significant laboratory parameters. Central reading of all ECGs,

demonstrated no notable effects, either during the dosing period or out to Day 28. Of the 6 serious adverse events (SAEs) reported in 4 patients, only 2 were regarded as possibly or probably related to pyronaridine artesunate administration.

A further dose escalation study (SP-003-05) is being conducted, in paediatric patients between 2 and 14 years old (body weight between 10 and 40 kg inclusive) with uncomplicated *P. falciparum* malaria at a study site in Gabon. Doses of pyronaridine artesunate of 6:2, 9:3 or 12:4 mg/kg are being studied in cohorts of 15 patients per dose level. Pharmacokinetics are studied in each patient. Additional endpoints include PCR-corrected and crude ACPR at Day 28 and safety outcomes including laboratory parameters, adverse events and ECGs.

2. STUDY OBJECTIVES

The primary objective of this clinical study is to compare the efficacy and safety of the fixed combination of pyronaridine artesunate (180:60 mg) with that of standard chloroquine therapy in children and adults with acute, uncomplicated *Plasmodium vivax* (*P. vivax*) malaria.

3. METHODS AND INVESTIGATIONAL PLAN

3.1. Study Design Overview

The study is designed as a multicentre, randomized, double-blind, double-dummy, parallel group, comparative trial.

Patients will be randomized to receive either oral pyronaridine artesunate (180:60 mg tablets), plus chloroquine-placebo or oral chloroquine (155 mg tablets), plus pyronaridine artesunate-placebo, once a day for 3 consecutive days. Patients will be followed for 42 days after the first study drug administration, the primary efficacy endpoint occurring at Day 14.

For patients who complete the study up to Day 28 and who have normal G-6-PD-activity, a 14-day course of primaquine (15 mg/day) shall be administered starting on Day 28, after all required assessments have been performed, to complete their radical cure. Patients who are deficient in G-6-PD and who complete the study up to Day 28 will be treated as per country policy. For all patients, a final safety assessment will be performed on Day 42.

3.2. Selection of Study Population

This study will be conducted in a total of 456 male and female children (≥ 20 kg body weight) and adult patients suffering from acute symptomatic uncomplicated *P. vivax* malaria recruited from trial sites located in South East Asia and India. The study will be randomised, with a maximum of 100 patients to be included per site.

Patients will be randomised in a 1:1 ratio to one of the following two treatment arms:

- Active-pyronaridine artesunate plus chloroquine-placebo
- Active-chloroquine plus pyronaridine artesunate-placebo

3.2.1. Inclusion criteria

Patients must fulfil the following inclusion criteria to be eligible for enrolment in the study:

1. Male or female patients between the age of 3 and 60 years, inclusive.
2. Body weight between 20 kg and 90 kg with no clinical evidence of severe malnutrition.
3. Presence of acute uncomplicated *P. vivax* mono-infection confirmed by:

a. Fever, as defined by axillary/tympanic temperature ≥37.5°C or oral/rectal temperature ≥38°C, or
   history of fever in the previous 24 hours (history of fever must be documented) **and,**
b. Positive microscopy of *P. vivax* with parasite density ≥250/ µL of blood (including at least 50% of asexual parasites)

4. Written informed consent, in accordance with local practice, provided by patient and/or parent/guardian/spouse. If the patient is unable to write, witnessed consent is permitted according to local ethical considerations.
5. Ability to swallow oral medication.
6. Ability and willingness to participate based on information given to patient or parent or guardian and access to health facility.

3.2.2. Exclusion criteria

To be eligible for enrolment in the study, patients must NOT meet any of the following criteria:

1. Presence of a mixed *Plasmodium* infection.
2. Presence of other clinical condition requiring hospitalization.
3. Presence of significant anaemia, as defined by Hb < 8 g/dL.
4. Known history or evidence of clinically significant disorders such as cardiovascular (including arrhythmia, QTc interval greater than or equal to 450 msec), respiratory (including active tuberculosis), hepatic, renal, gastrointestinal, immunological (including active HIV-AIDS), neurological (including auditory), endocrine, infectious, malignancy, psychiatric or other abnormality (including recent head trauma).
5. Known history of hypersensitivity, allergic or adverse reactions to pyronaridine, chloroquine or artesunate or other artemisinins.
6. Known history of hypersensitivity, allergic or adverse reactions to chloroquine, primaquine and related agents.
7. Known active Hepatitis A IgM (HAV-IgM), Hepatitis B surface antigen (HBsAg) or Hepatitis C antibody (HCV Ab).
8. Known seropositive HIV antibody.
9. Have received any antimalarial treatment in the preceding 2 weeks, as determined by history and, whenever feasible, by screening test.
10. Have received antibacterial with known antimalarial activity in the preceding 2 weeks.

11. Have received any investigational drug within the past 4 weeks.
12. Liver function tests (AST/ALT levels) more than 2.5 times the upper limit of normal range.
13. Known significant renal impairment as indicated by serum creatinine levels of more than 1.4 mg/dL.
14. Female patients of child-bearing potential must be neither pregnant (as demonstrated by a negative pregnancy test) nor lactating, and must be willing to take measures to not become pregnant during the study period.
15. Previous participation in the present clinical trial with pyronaridine artesunate.

3.3. Patient Discontinuation or Protocol Violation

A patient may withdraw consent at any time, without prejudice for further follow up or treatment by the study site.

A patient may be discontinued prematurely from the study if the Investigator decides that a patient should be withdrawn for safety reasons, due to an adverse event of such a nature or intensity (including abnormal laboratory finding), where study drug withdrawal or withdrawal from the study is advisable. Information concerning any adverse event and symptomatic treatment given must be recorded on the case record form (CRF). If the adverse event is serious, the Investigator must notify the Sponsor or its designee immediately and the reporting procedures described in the safety section must be followed. Definitions and reporting requirements for adverse events are provided in section 5.2.4.

Self or third party administration of antimalarial (or antibiotics with antimalarial activity) treatment other than for treatment failure will be classified as a protocol violation. A list of Forbidden Medication is provided in Attachment 5.

In the case of a patient vomiting the first dose (Day 0) within 0 to 30 minutes of study drug administration, a repeat full dose will be given. If patient vomits the study drug on either the second (Day1) or third day (Day 2) of dosing or if the patient vomits a re-administered dose on Day 0, they will not be re-dosed but will be withdrawn from the study. A patient who is withdrawn due to vomiting should receive rescue medication, after having completed end of study (Day 28) assessments.

A patient who terminates participation after any study drug administration for any reason will be considered as having discontinued from the study and will have

end of study (Day 28) procedures performed. The reason for discontinuation will be recorded on the CRF. If a patient is discontinued for an adverse event, the Investigator will continue to follow the patient until resolution or until no further medically relevant information can be expected.

Every effort must be made to schedule a follow-up visit for patients who fail to return to the study site after administration of the study drug. Women who inadvertently become pregnant during the study must be followed to full term and the outcome of the birth fully documented on a specific form provided by Sponsor.

4. STUDY DRUG ADMINISTRATION PROCEDURES

4.1. Patient Assignment and Blinded Administration

Patients who meet all entry criteria and present no exclusion criteria will be randomised to receive either pyronaridine artesunate (plus matching placebo of chloroquine) or chloroquine (plus matching placebo of pyronaridine artesunate) in a 1:1 ratio according to the randomisation scheme provided by the Sponsor or designee. The randomisation scheme will be generated using permutation blocks technique. Complete blocks will be assigned to each study site. Patients will be assigned, in ascending order, a randomisation number according to the order recruited. The patient will be allocated an individual numbered treatment pack which contains sufficient tablets for 3 days therapy plus an overage bottle containing tablets in case the patient vomits the first dose.

The Investigator will measure the patient's body weight at entry and refer to the chart (Attachment 4) to calculate the actual number of tablets to be administered daily.

Patients will be allocated the following identification nomenclature according to the order recruited within a given site: **site code-patient number.**

Clinical trial material will be administered using a double blind design. That is: neither the Investigator neither the patient will be aware of the treatment arm to which the patient has been assigned.

4.2. Materials and Supplies

4.2.1. Study drug: pyronaridine artesunate or matching placebo

Pyronaridine artesunate and its matching placebo will be supplied by Shin Poong Pharm Co. Ltd in high density polyethylene bottles as numbered individual treatment allocations. The active ingredients of the investigational product are pyronaridine tetraphosphate and artesunate.

The tablet strength to be used is 180:60 mg pyronaridine artesunate. Depending on their body weight, patients will receive between 1 to 4 tablets per day (see Attachment 4). The actual dose-level range covered by this regimen is between 7.2:2.4 mg/kg and 13.8:4.6 mg/kg pyronaridine artesunate, respectively.

Pyronaridine artesunate and its matching placebo clinical trial material will be packaged in 3 bottles corresponding to each dosing day and each containing 4 tablets. Unused tablet shall be kept in their original packaging and remain the Investigators' responsibility for drug accountability. A fourth bottle will be provided as an overage of one day's supply, in the event a patient requires re-dosing due to vomiting the first day's treatment (Day 0). According to the randomization list, the tablets will contain either active drug or a placebo with matching aspect, color and taste.

Example Product Label for pyronaridine artesunate:

**Product for Clinical Study Use ONLY**
Study Reference: **SP-C-006-06**
Patient number:
Study Visit: *Day 0 (1 or 2 or Overage)*
Contains: *4 Tablets*
Each tablet contains:
pyronaridine *180 mg* / artesunate *60 mg* or matching placebo
Storage: Below 30 degrees C
Lot No.:
Expiration date:
To be administered by Study Staff only
Sponsor: SHINPOONG PHARM. Co., Ltd., Republic of Korea

4.2.2. Chloroquine or matching placebo

The comparator drug chloroquine and its matching placebo will be supplied by Shin Poong Pharm Co. Ltd. Clinical trial material will be presented in high density polyethylene bottles as numbered individual treatment allocations.

The dosage to be used for adults is 620 mg chloroquine on Days 0 and 1 and 310 mg on Day 2. The tablet strength to be used is 155 mg chloroquine base, which

will provide a posology for adults of 4 tablets on Days 0 and 1 and 2 tablets on Day 2. The dosage to be used for children is 10 mg/kg chloroquine base (i.e. between 1 and 3 tablets, depending on the child's weight) on Days 0 and 1 and 5 mg/kg (i.e. between 0.5 and 1.5 tablets depending on the child's weight) on Day 2. The dosage should not exceed the adult dose regardless of weight (see Attachment 4).

Chloroquine and its matching placebo clinical trial material will be packaged in 3 bottles corresponding to each dosing day. The bottles for Day 0 and 1 will contain 4 tablets, while the bottle for Day 2 will contain 2 tablets. Unused tablets of chloroquine or matching placebo shall be kept in their original packaging and remain under the Investigator's responsibility for drug accountability. A fourth bottle containing 4 tablets will be provided as an overage of the first day's supply, in the event a patient requires re-dosing due to vomiting the first day's treatment (Day 0). According to the randomization list, the tablets will contain either active drug or a placebo with matching aspect, color and taste.

Example Product Label for chloroquine:

Product for Clinical Study Use ONLY

Study Reference: **SP-C-006-06**

Patient number:

Study Visit: *Day 0 (or 1 or Overage)*

Contains: 4 *Tablets*

Each tablet contains:

chloroquine base 155 mg or matching placebo

Storage:

Lot No.:

Expiration date:

To be administered by Study Staff only

Sponsor: SHINPOONG PHARM. Co., Ltd., Republic of Korea

Product for Clinical Study Use ONLY

Study Reference: **SP-C-006-06**

Patient number:
Study Visit: *Day 2*

Contains: 2 *Tablets*
Each tablet contains:
chloroquine base 155 mg or matching placebo
Storage:
Lot No.:
Expiration date:
To be administered by Study Staff only
Sponsor: SHINPOONG PHARM. Co., Ltd., Republic of Korea

4.2.3. Primaquine

Primaquine will be supplied by Shin Poong Pharm Co. Ltd. Clinical trial material will be presented in an open-label manner, in high density polyethylene bottles.

The dosage to be used for adults is 15 mg primaquine on Days 28 through 42. The tablet strength to be used is 26.3 mg primaquine phosphate (equivalent to 15 mg primaquine base), which will provide a posology for adults of 1 tablet every day for 14 consecutive days, starting on Day 28 (see Attachment 4).
The dosage to be used for children is 0.5 mg/kg primaquine phosphate (equivalent to 0.3 mg/kg primaquine base) per day (see Attachment 4).

Note: Due to the risk of haemolytic anemia in G-6-PD-deficient patients, primaquine shall be administered only to patients with normal G-6-PD activity (as determined at baseline). Furthermore, for women of childbearing potential, primaquine will only be administered once a negative pregnancy test has been demonstrated.

Example Product Label for primaquine:

Product for Clinical Study Use ONLY

Study Reference: **SP-C-006-06**

Patient number:

Study Visit: *Day 28 through Day 42*

Contains: 14 *Tablets*

Each tablet contains:

primaquine base 15 mg

Storage:

Lot No.:

Expiration date:

To be administered by Study Staff only

Sponsor: SHINPOONG PHARM. Co., Ltd., Republic of Korea

4.3. Sponsor's Responsibilities

4.3.1. Study drug: pyronaridine and artesunate

Pyronaridine artesunate and its matching placebo will be manufactured by Shin Poong Pharm Co. Ltd in accordance with appropriate quality (GMP) standards. The manufacturer will supply certificates of analyses and a statement of the expiry or re-test dates. Drug supplies will be shipped to the Investigational site after all necessary ethical and local authority's approval have been provided.

Shin Poong will provide study drug packaged to prevent contamination or deterioration during transport and storage. Shin Poong have determined acceptable storage temperatures, conditions and times to be observed for study drug and will inform the study site of these. Expiry dates are provided on the labelled study material.

4.3.2. Comparator drug: chloroquine

Chloroquine and its matching placebo will be manufactured by Shin Poong Pharm Co. Ltd in accordance with appropriate quality (GMP) standards. Shin Poong Pharm Co. Ltd will supply certificates of analyses and a statement of the expiry or re-test dates. Drug supplies will be shipped to the Investigational site after all necessary ethical and local authority's approval have been provided.

Shin Poong will provide study drug packaged to prevent contamination or deterioration during transport and storage. Shin Poong have determined acceptable storage temperatures, conditions and times to be observed for study drug and will inform the study site of these. Expiry dates are provided on the labelled study material.

4.3.3. Primaquine

Primaquine will be manufactured by Shin Poong Pharm Co. Ltd in accordance with appropriate quality (GMP) standards. Shin Poong Pharm Co. Ltd will supply certificates of analyses and a statement of the expiry or re-test dates. Drug supplies will be shipped to the Investigational site after all necessary ethical and local authority's approval have been provided.

Shin Poong will provide study drug packaged to prevent contamination or deterioration during transport and storage. Shin Poong have determined acceptable storage temperatures, conditions and times to be observed for study drug and will inform the study site of these. Expiry dates are provided on the labelled study material.

4.4. Storage Conditions

Clinical trial material is to be stored under the strict conditions stipulated by Shin Poong Pharm.
Stability testing has been conducted prior to the conduct to this trial to confirm the stability of the clinical trial material.

4.5. Drug Administration

Study drug will be administered to patients by a qualified member of the study site who has been designated by the Investigator. All parties will be blind to treatment assignment and will not be informed of treatment allocation unless the blind needs

to be broken for a medical emergency. Sealed envelopes with treatment allocation will be available at each site to be opened only in case of emergencies. Patient's weight at screening should be used to calculate the number of tablets to be administered.

Study drug will be given to each patient with up to 240 ml (full glass) water. All tablets must be swallowed. Patients will take the medication in an upright position (seated or standing). The patient may be given food prior to drug administration.

Initial drug administration day is designated Day 0. A dispensing record detailing the number of tablets administered for each patient, as well as the date and time, will be maintained. Exact time of dose administration will be recorded in the CRF.

Dosing on the two subsequent days will occur no less than 10 hours after the previous dosing. Exact time of daily dose administration will be recorded in the CRF.

In the case of a patient vomiting the first dose (Day 0) within 0 to 30 minutes of study drug administration, a repeat full dose will be given. If patient vomits the study drug on either the second (Day 1) or third day (Day 2) of dosing or if the patient vomits a re-administered dose on Day 0, they will not be re-dosed and will be withdrawn from the study. A patient who is withdrawn due to vomiting must be given rescue medication.

Unused medication will remain in a locked cabinet in the pharmacy until full accountability has been performed by the Sponsor or its designee and instructions given for their return to Shin Poong.

Patients with normal G-6-PD activity (as at screening) and who complete the study up to Day 28 will be given a 14-day course of primaquine (15 mg/day) in an open-label manner starting on Day 28, after all required assessments have been completed. For women of childbearing potential, a negative pregnancy test should be demonstrated before starting primaquine treatment. Primaquine treatment should not be administered to pregnant women.

4.6. Emergency Unblinding

Sealed envelopes containing the study medication assignment for each patient will be provided to the Investigator. The envelopes will be retained by the Investigator (or designee) in a secure area and may be may be opened in case of emergency only if the Investigator(s) need to know the study medication in order to manage the patient's condition. The Sponsor must be notified before breaking the blind, unless identification of the study medication is required for emergency

therapeutic measures. The Sponsor must then be notified within 24 hours of the blind being broken. The date that the blind was broken, the reason for the unblinding and the name of the person performing the unblinding must be recorded in the source documents and on the CRF. All code break envelopes (opened and unopened) will be returned to the Sponsor at the end of the study.

4.7. Compliance

Every attempt will be made to select patients and carers who have the ability to understand and comply with instructions, in particular the requirements for patients to attend the scheduled study visits.

The staff member designated by the Investigator to administer the study drug will ensure that each patient receives and swallows the appropriate number of tablets by direct observation and monitoring of drug administration. Monitors will perform drug counts and compliance for each patient on an ongoing basis during monitoring visits. Drug accountability records will be maintained by the designated clinical study site personnel on specific documentation forms provided by the Sponsor.

4.8. Study Site's Responsibilities

Upon receipt of study drug, the Principal Investigator will conduct an inventory and sign the accompanying receipt and return this to the Sponsor.

Study drugs packs will be stored under the control of the Principal Investigator in a locked cabinet in a secure facility appropriate for the advised storage conditions and nature of products.

Clinical trial material may be used only in the conduct of this study and any unused medication must be returned as instructed by Shin Poong on completion of the study.

4.9. Clinical Study Material Accountabillity

A drug accountability utilisation log will be maintained for clinical study material. This will include the date and time of administration, quantity administered, lot number and patient randomisation number. The log should be signed and dated by the dispenser.

At the end of the study, accountability will be checked by the Sponsor or designee, and the Sponsor and the Principal Investigator will retain copies of the complete logs.

Unused supplies will be returned in accordance with written instructions from Shin Poong.

4.10. Concomitant and Forbidden Medication

Patients should not have taken any other antimalarial therapy in the 2 weeks preceding the first study drug administration, as evidenced by the Saker Solomon and Lignin tests.
Treatment of fever over 38.0°C (axillary/tympanic) or 38.5°C (oral/rectal) with paracetamol (acetaminophen) up to 1000 mg, according to age of patient, is permitted. Concomitant disease and/or adverse events requiring treatment may be treated according to local practice.

Antimalarials and antibiotics with antimalarial activity including macrolides and cyclins are not allowed during the study except tetracycline for ophthalmic use. A list of forbidden medication is provided in Attachment 5.

Name (generic), dosage (only for antipyretics), dates of administration and reason for administration of any medication given during the study must be recorded in the CRF. If the medication is given to treat an adverse event, the appropriate adverse event form must be completed.

4.11. Rescue Medication

Treatment failures will be treated with the best antimalarial therapy available in the country where the study is conducted. All procedures to be performed at Day 28, including sampling for PCR, preparation of a thin and thick films and **whole blood sample for measurement of pyronaridine and/or chloroquine/chloroquine metabolites blood levels** must be undertaken before rescue medication is administered.

5. ASSESSMENTS

5.1. Efficacy Assessments

5.1.1. Parasitological assessments

5.1.1.1. Microscopy

Parasite density expressed as the number of parasites per microlitre (µL) of blood will be measured serially to determine parasite clearance time (PCT). Asexual parasite and gametocyte counts will be recorded separately at screening, while total parasite counts will be recorded at any timepoint thereafter. Blood sampling for parasitology will be done usually by means of finger prick except when the timing for parasitology assessments coincide with time for clinical laboratory tests, in which cases, blood smears (thick and thin) and blood drop for PCR can be taken from the venous blood collected for clinical laboratory analyses.

Blood smears preparation, staining, examination and interpretation should be in accordance with the guidelines provided by the Sponsor. Any deviation from the Sponsor guidelines must be discussed and approved by the Sponsors before the study commences.

Thick and thin blood films for parasite count should be obtained and examined at screening on Day 0 to confirm inclusion/exclusion criteria. Thick blood films will be examined every 8 hours (+/- 1 hour) following first dose administration and for at least 72 hours (Day 1 and Day 2) or until the parasites have cleared as evidenced by two consecutive negative slide readings taken 8 to 24 hours apart. To avoid disruption of patient sleep at night, the first interval between the first dose administration and the next smear may be shorter or longer than 8 hours but shall not exceed 12 hours.

Thick blood films will be also examined on Days 3, 7, 14, 21, 28, 35 and 42 or on any other day if the patient spontaneously returns. Additionally, blood films should be obtained whenever parasitological reassessment is required. A thin blood smear will also be taken from Day 7 whenever parasitological blood samples are taken, and reserved in the event of recrudescence, to confirm Plasmodium species.

Parasitaemia Blood Sampling Schedule for the first 72 hours

Hours	0	8	16	24	32	40	48	56	64	72
PCR sample	1	0	0	1	0	0	0	0	0	0
No of thick films	2	2	2	2	2	2	2	2	2	2
No. of thin films	2	0	0	0	0	0	0	0	0	0

Parasitaemia Blood Sampling Schedule Days 7 to 42

	Day 7	Days 14, 21, 28, 35 and 42	Day of failure	Any additional visit
PCR Sample	1	1	1	0
No. of thick films	2	2	2	2
No. of thin films	1	1	1	1

The method of counting parasites will be described in the Study Laboratory Procedures Manual including gametocyte counts.

Local quality control of slides is to be assured by reading of slides by 2 different qualified microscopists, reporting independently, with the arithmetic mean of the 2 counts being recorded in the patient record and CRF. In the case of discrepancy, a third microscopist will review the slides and the Site Principal Investigator will make the final classification to be reported in the CRF.

An external quality control of slide reading will be established by an independent laboratory at Swiss Tropical Institute (STI), Basel, Switzerland. The external quality control will be blinded to treatment assignment. STI will examine all slides in the case of suspected recrudescence plus a proportion of slides from each study site. The procedure for quality control will be described in the Laboratory Procedures Manual.

5.1.1.2. Genotyping studies by PCR

Genotyping by PCR will be used as an exploratory tool to differentiate re-infection (New infection) from recrudescence or relapse (relapse being parasitemia originating from latent hypnozoites).
Samples will be taken for PCR assessment prior to initial dosing on Day 0 and again prior to Day 1 dosing. PCR samples will also be taken from Day 7 whenever parasitological blood samples are taken, and reserved. The PCR analysis will, however, only be performed in the case of reappearance of parasites as judged by a positive microscopy slide.

A common technical protocol will be used for PCR sampling and analysis. PCR samples will be shipped by courier for centralised assessments at STI, Basel, Switzerland for reporting of PCR data conducted for patients classified as treatment failure. Instructions for sampling and storing filter paper for molecular marker studies will be provided in a Laboratory Procedures Manual.

5.1.2. Clinical efficacy assessments

5.1.2.1. Body temperature

Body temperature will be measured at screening on Day 0 (Pre-Dose) and every 8 hours over at least 72 hours following first study drug administration or when fever has normalised (<37.5°C axillary/tympanic or <38°C oral/rectal): 2 normal readings taken between 8 and 24 hours apart. Temperature will be measured at every visit thereafter and as clinically indicated.

Temperature methodology shall be according to local practice. For each individual patient, the same means of temperature measure (axillary, oral, tympanic, rectal) should be used throughout the entire study period. Patients who have had a documented reported history of fever (≥37.5°C axillary/tympanic or ≥38.0°C oral/rectal) prior to screening should be monitored closely for fever peaks.

Patients entered in the study on the basis of history of fever and who do not subsequently have at least one body temperature measurement indicating presence of fever will not be included in the analysis of fever clearance time.

Quality of temperature-taking technique and thermometers should be checked and calibrated prior to study commencement and at regular intervals during study.

5.1.2.2. Clinical signs and symptoms of malaria

Clinical signs and symptoms of malaria will be assessed during the treatment period, plus on any other occasion where the patient presents to the site, when clinically indicated. The following signs and symptoms will be specifically assessed: rigors/chills, sweating, headache, nausea, vomiting, cough, loss of appetite/anorexia, fatigue (asthenia/lethargy/malaise), myalgia (back and limbs), jaundice (hyperbilirubinemia), hepatomegaly and splenomegaly.
If any sign or symptom of acute malaria represents a clinically significant worsening compared to the patient's baseline condition, which are considered to be more severe than normal daily fluctuation of the disease process, then this should be recorded as an adverse event.

5.1.3. Drug levels at time of treatment failure

At time of suspected treatment failure, prior to administration of rescue treatment, a 1 mL of whole blood sample will be drawn for assay of pyronaridine and/or chloroquine (and metabolites) levels.
For full details on sample handling procedures, see Attachment 6.
The actual times of sampling will be recorded in the CRF.

5.1.4. Population pharmacokinetics

At selected clinical study sites, as determined by the Sponsor, the Investigator is required to collect samples as follows:

- Sampling for Pyronaridine Population Pharmacokinetics: 2 blood samples from each patient at two different timepoints. One sample will be drawn between Day 0 (post dose) and Day 3, and a second between Day 4 and Day 42.
- Sampling for Artesunate Population Pharmacokinetics: 2 plasma samples from each patient at two different timepoints. One sample will be drawn on Day 0 (post-dose) or Day 2, and a second one on Day 3.

A list of timepoints will be generated corresponding to each patient number and provided to the Principal Investigator. Actual time must be recorded

5.2. Safety Assessments

Safety will be assessed using the following criteria:

- Nature, incidence, relationship and severity of adverse events (AEs) and serious adverse events (SAEs). Adverse event reporting will be assessed through direct questioning.
- Significant change from baseline clinical laboratory parameters
- Incidence of and reasons for withdrawals
- Physical examinations and vital signs
- 12-Lead ECG assessments

5.2.1. Clinical safety assessments

Clinical evaluations will be undertaken in all patients using the following parameters:

5.2.1.1. Physical examination

A standard physical examination will be performed at baseline (Day 0 pre-dose), as well as on Days 3, 28 and 42, and in case of early termination. At a minimum the following body systems will be examined: general appearance; head and eyes; ears, nose and throat; chest and lungs; cardiovascular; abdomen; neurological; abdomen; lymphatic and musculoskeletal.
A complete medical history, demography and contact address and details will be taken at baseline.

5.2.1.2. Body weight and height

Body weight and height will be recorded at screening on Day 0 (pre-dose). The screening weight will be used to satisfy the inclusion/exclusion criteria for minimum/maximum weight as well as to calculate the number of tablets of study drug (as detailed in Attachment 4) to be administered. Body weight will be measured again on Day 28 to calculate the number of primaquine tablets to be administered (as detailed in Attachment 4).

The reliability of weighing scales will be verified prior to commencement of study and will be checked and documented at regular intervals during the study.

5.2.1.3. Vital signs

Vital signs (supine blood pressure and heart rate) will be taken after the patient has been supine for 5 minutes. Vital signs will be measured at baseline (Day 0 pre-dose) and on Days 1, 2, 3, 7, 14, 21, 28, 35 and 42 and in the case of early termination. In addition, vital signs may be measured during the study period, as clinically indicated.

5.2.2. Laboratory assessments

Clinical laboratory tests are to be performed as indicated in Table 1.

Haematology parameters are to be measured on Days 0, 3, 7, 28 and 42 and, if clinically indicated, on other visits.

Biochemistry and urinalysis are to be measured on Days 0, 3, 7 and 28, as well as Day 42 if clinically indicated.

Female patients of child-bearing potential will have a urine hCG test for pregnancy prior to inclusion and on Days 14, 28 and 42.

Clinical laboratory tests will require 2 ml for haematology and 2 ml for clinical chemistry, plus a urine sample for dipstick analysis.

Table 1 Clinical Laboratory Tests

**Haematology:**	**Biochemistry (serum):**
Haematocrit<br>Haemoglobin<br>Erythrocyte count (RBC)<br>Platelet count<br>Leukocytes (WBC) with differential count including Eosinophils<br><br>**Urinalysis:**<br>Dipstick for presence of glucose, protein and blood<br><br>**Pregnancy test (urine hCG)** *<br>**G-6-PD testing** **	Total bilirubin<br>Albumin<br>Alanine aminotransferase (ALT/SGPT)<br>Aspartate aminotransferase (AST/SGOT)<br>Creatine kinase<br><br>Alkaline phosphatase<br>Urea<br>Creatinine<br><br>Sodium<br>Potassium<br>Glucose

* For females of childbearing potential only
** At screening only

Entry Laboratory Screen

At a minimum the results of the following tests must be available before the patient is randomised:

- Haemoglobin
- Urea
- Sodium
- Potassium
- Blood glucose
- Alanine aminotransferase (ALT/SGPT)
- Aspartate aminotransferase (AST/SGOT)
- Creatinine
- Pregnancy test (Urine hCG)

If the results of baseline laboratory evaluations of other haematology, biochemistry and urine parameters fall outside of the normal range and are regarded as clinically significant, the Study Medical Monitor should be notified.

The estimated total volume of blood that will be drawn from each patient in this study is presented in Table 2.

Table 2 Volume of blood to be drawn from each patient over 42 days

Assessment		Sample volume (ml)	No. of samples	Total volume (ml)
Pharmacokinetic Drug levels*		**1**	**4**	**4**
**Pharmacokinetic**	**Treatment failure only**	**1**	**1**	**0 to 1**
Safety	Biochemistry	2	4 to 5	8 to 10
	Haematology	2	5	10
Total				**22 to 25**

*** at selected sites only**

Laboratory values that fall outside a clinically accepted reference range must be evaluated and commented on by the Investigator, and graded according to the DMID Toxicity Scale for Determining Severity of Adverse Events (Attachment 7). Generally, clinically significant abnormalities that represent a change from baseline and could be considered to be detrimental are to be recorded as adverse events. It is anticipated that a fall in haematological parameters such as haemoglobin may occur during treatment and, in general falls of up to 2 g/dL need not be reported as adverse events unless that fall is considered to be detrimental to the patient. Detrimental changes in differential white cell count (other than clinically significant falls in neutrophils) should only be reported as adverse events

if their change is considered to be contributing to deterioration on the patients' condition.

Investigators must document their review of each laboratory report by signing and dating each report. Laboratory data will be transcribed into the CRF. The laboratory reports are to be kept with the CRF until source data verification has been performed by the monitor and then it should be filed with the source document for the study.

5.2.3. ECG

The incidence and nature of clinically significant ECG abnormalities will be assessed.

A 12-lead resting ECG will be performed at screening on Day 0 (Pre-Dose), and approximately 2 - 4 hours after study drug administration on Day 2, as well as on Day 28. Further ECG measurements at other times may be taken as clinically indicated.

Clinical indications for further ECG recordings would be the finding of obvious abnormalities of rhythm, and/or obvious changes from the previous ECG or if the patient experiences cardiac-type symptoms such as chest pain, palpitation or shortness of breath. If the ECG recording device provides an automated report then this can be used as a guide as to whether a further ECG would be required. The automated report should not, however, be the only source of information as to whether an abnormality is present for reporting purposes.

For each ECG recording, care must be taken to ensure that the leads are placed in the same place at each time point. Only one recording is required, however, after the recording has been made, on each occasion the ECG should be inspected to ensure that it is of sufficient quality for interpretation (i.e. all of the leads are in their correct place and there is minimum artefact). If the quality is not adequate the recording should be repeated. Once this has been performed a copy should be made from the original recording. One copy of the ECG should be included with the CRF and the other with the source documents.

The ECG recording should be read and interpreted by the Investigator. Should changes from baseline be present, the Investigator should assess whether these are clinically significant and if so record the findings on the CRF and if considered a detrimental change, record as an adverse event. A proportion of ECGs from

each site will be sent to a central blinded reviewer for review. A report on the central review will be sent back to site.

The ECG should be reviewed for:

1. Waveforms P, QRS and T as well as the presence of U waves
2. Rhythm (abnormal rhythms as well as tachycardia and bradycardia)
3. Evidence of abnormalities of PR, QT, and ST segments
4. An assessment of overall "normality" of the ECG and where borderline or abnormal whether the abnormality is clinically significant
5. Any change from baseline or from one ECG to the next
6. Where an ECG is considered abnormal at baseline consideration, as to whether an exclusion criterion has been violated, will need to be considered
7. Where an ECG exhibits a change this will need to be recorded and clinical significance assigned (Yes/No); if clinically significant then an AE should be recorded if appropriate.

5.2.4. Adverse events

5.2.4.1. Adverse event definition

The following definitions will be employed for the reporting of Adverse Events:

A) Adverse Event:

An adverse event is defined as any unfavourable and unintended sign, symptom, syndrome, or illness that develops or worsens during the period of observation in the clinical study. Clinically relevant abnormal results of diagnostic procedures including abnormal laboratory findings (e.g., requiring unscheduled diagnostic procedures or treatment measures, or resulting in withdrawal from the study) which are considered by the Principal Investigator to be detrimental should be recorded as adverse events whether or not they have a causal relationship with the study drug. Where the laboratory result is a sign of another clinical condition, the clinical condition itself should be the reported adverse event. An event unequivocally caused by a significant deterioration of the underlying condition related to malaria is regarded as an adverse event.

Occurrence of *P.falciparum* infection occurring during the course of the study will be captured on a specific form on the CRF. However these events will not be reported as adverse events unless the resulting malaria is of an unexpected severity.

B) Unexpected Adverse Event:

Any adverse experience that has not been previously observed (i.e. included in the protocol or Investigator's Brochure) whether or not the event is anticipated because of the pharmacologic properties of the study drug.

C) **Serious** Adverse Event

Any adverse experience occurring at any dose that results in any of the following outcomes:

- **Death.**
- **Life threatening**- defined as an experience that places the patient or subject, in the view of the Investigator, at immediate risk of death from the reaction as it occurred. (Note: this does not include a reaction that, had it occurred in a more severe form, might have caused death).
- Requires inpatient **hospitalisation** or prolongation of existing hospitalisation.
- Results in a **congenital** anomaly or birth defect.
- Results in a **persistent or significant disability** or incapacity.
- **Important medical events** that may not result in death, be life-threatening or require hospitalisation may be considered as a serious adverse drug experience when, based upon appropriate medical judgement, they jeopardise the patient or subject and may require medical or surgical intervention to prevent one of the outcomes listed above (for example, an event may be defined as serious based on a deterioration in grade in Toxicity Tables).

"Persistent or significant disability or incapacity" means that there is a substantial disruption of a person's ability to carry out normal life functions.

Medical and scientific judgement should be exercised in deciding whether expedited reporting is appropriate in situations where none of the outcomes listed above occurred. Important medical events that may not be immediately life-threatening or result in death or hospitalisation but may jeopardise the patient's health or may require intervention to prevent one of the other outcomes listed in the definition above should also usually be considered serious.

The following will not be considered as Serious Averse Events: cases of hospitalisation for elective surgery, need to observe high risk patients to prevent serious events, and visiting the emergency room due to suffering from adverse events but discharged after treatment without hospitalisation.

D) Alert terms and other reasons for expedited reporting to Pharmacovigilance
No specific events are subject to reporting as alert terms in this study.

Pregnancy should be reported **promptly** for tracking purposes but does not constitute as such an adverse event. The pregnancy should be reported on the Pregnancy Report Form. The pregnant patient must be followed until birth of the child and the outcome of the pregnancy and birth reported to the Sponsor. However, any adverse outcome to the mother, foetus or newborn must be regarded as a serious adverse event and must be **immediately** reported to the Sponsor.

Worsening of the baseline conditions that are judged by the Investigator to be more significant than normal daily fluctuation of the disease symptoms should be regarded as adverse events.

Prior to randomisation, study site personnel will note the occurrence, nature and severity of each patient's medical condition(s). During the study, site personnel will note any change in the condition(s) and/or the occurrence and nature of any adverse events.
At each visit the patient will be asked about any change in his/her condition since the last evaluation.

5.2.4.2. Assessment of adverse events

A) Severity

The Investigator will assess the severity/intensity of the adverse reactions and clinical laboratory changes using the DMID Toxicity Grading Scale for Determining the Severity of Adverse Events provided in Protocol Attachment 7.

If an adverse event is not listed in the DMID table, the Investigator will assess the severity using the following guidelines:

1 = Mild: awareness of sign or symptom, but easily tolerated
2 = Moderate: enough discomfort to cause interference with usual activity

3 = Severe: incapacitating with inability to work or do usual activity

4 = Life-Threatening

Clarification of the difference in meaning between "severe" and "serious"

The term "severe" is often used to describe the intensity (severity) of a specific event (as in mild, moderate, or severe myocardial infarction); the event itself, however, may be of relatively minor medical significance (such as severe headache). This is not the same as "serious", which is based on the outcome or criteria defined under the serious adverse event definition. An event can be considered serious without being severe if it conforms to the seriousness criteria, similarly severe events that do not conform to the criteria are not necessarily serious. Seriousness (not severity) serves as a guide for defining regulatory reporting obligations.

B) Relationship or Association with the Use of Study Drug or Study Procedure

The Investigator will assess the relationship of the event with the study drug using the following guidelines and terminology:

Definite: clear-cut temporal association, with a positive re-challenge test or laboratory confirmation.

Probable: clear-cut temporal association, with improvement upon drug withdrawal, and not reasonably explained by the patient's known clinical state.

Possible: less clear temporal association; other aetiologies are possible. Other possible aetiologies should be recorded on the CRF.

None: no temporal association with the study drug; related to other aetiologies such as concomitant medications or conditions, or subject's known clinical state.

C) Action Taken

- Study drug discontinued
- Patient withdrawn from study
- Concomitant Medication required
- Hospitalisation required or prolonged (this should also be reported as a Serious Adverse Event
- Other

D) Outcome

The Investigator will follow-up the AE until resolution or until no further medically relevant information can be expected. AE outcome will be classified as follows:

- Resolved
- Resolved with sequelae
- Continuing
- Death

5.2.4.3. Adverse event reporting requirements

All adverse events must be recorded on the standard Adverse Events Form in the CRF. All serious adverse events occurring during the study must be recorded on the SAE Form. These reports are to be made whether or not the Investigator considers the serious adverse events to be related to the investigational drug.

A full written report to the Sponsor's Medical Monitor will include the SAE Form, photocopies of pertinent test results and medical notes / study records, consultant report(s), hospital discharge summary and autopsy report (if relevant and as soon as available to the Investigator), and a summary of the outcome of the reaction plus the Investigator's opinion of drug relationship to the serious adverse event(s). The provision of this report must not delay reporting of the SAE to the Sponsor as described below.

The Investigator or his / her staff will notify the Independent Ethics Committee (IEC) of all SAEs as soon as possible and in accordance with local regulations.

Adverse events and / or laboratory abnormalities identified in the protocol as critical to safety evaluations shall be reported by the Investigator to the Sponsor according to the reporting requirements and within the time periods specified for serious adverse events.

5.2.4.4. Serious adverse event reporting procedures

Study site personnel must report any serious adverse event immediately by telephone (within 24 hours of becoming aware of the event) to the Regional Medical Monitor or Central Medical Monitor if the Regional Medical Monitor is not available. The Regional or Central Medical Monitor will notify the

Pharmacovigilance group (AKOS Ltd) with 1 working day. The Investigator must send SAE form to AKOS and alert monitoring personnel within 24 hours of knowledge of the serious event (via fax or email with scanned form or phone call followed by form as soon as possible). The pharmacovigilance group will notify the Regional Medical Monitor, Central Medical Monitor, study project managers and Sponsors (MMV, Shin Poong) and other parties as specified, within 1 working day of receipt. The exception to above: a life-threatening or fatal event regardless of relationship will be notified to the Central Medical Monitor immediately (within 2 hours of receipt) by phone.

The Pharmacovigilance contact details for SAE notification are as follows:

**Maria Hilling**<br>Manager of Pharmacovigilance Operations<br>Akos Limited<br>Herts, AL5 1AH,<br>United Kingdom<br>**Tel: +44 (0)1582 766339**<br>**Fax: +44 (0)1582 764327**<br>**Email: maria.hilling@akos.co.uk**

Regional Medical Monitor:

**George Watt**<br>Family Health International<br>19th floor, Tower 3, Sindhorn Building<br>130-132, Wireless Road<br>Kwaeng Lumpini, Khet Phatumwan<br>Bangkok 10330, Thailand<br>Tel: +66 2 263 2300 ext 139<br>Cell +6681 814 1032<br>Home: +66 2 966 8001<br>Email: gwatt@fhibkk.org

Central Medical monitor:

**Robert Miller**<br>Fulcrum Pharma Developments<br>Hemel One<br>Boundary Way<br>Hemel Hempstead,<br>Hertfordshire HP2 7YU,<br>United Kingdom<br>Tel: +44 870 710 4360<br>Fax: +44 870 710 4360<br>Email: robert.miller@fulcrumpharma.com

The Investigator will be required to provide the following information:

- **Patient's ID number, date of birth and gender**
- **Description of the event**
  - Date, time of onset
  - Clinical history
  - Associated signs and symptoms
  - Temporal association with the study drug
  - Medical management, including rationale
  - Pertinent laboratory tests
  - Severity (see definition or toxicity score)
  - Causal relationship to the study drug
- **Other information**
  - Relevant past medical history
  - Concomitant medications
  - Autopsy report or expectation of an autopsy in the case of death
- **Outcome of the event**
  - Date, time of resolution, if resolved
- **Documentation of notification to the IEC**

If a patient's dosage is reduced or treatment is discontinued as a result of an adverse event, study site personnel must clearly document the circumstances and data leading to any such dosage reduction or discontinuation of treatment.

5.2.4.5. Safety monitoring plan

The assigned Regional Medical Monitors and Study Medical Monitor will review the safety information based on routine monitoring reports and status reports as instructed by Sponsor. Investigators will be required to provide a weekly status report indicating occurrence of treatment failure or clinically relevant findings. Safety oversight will be detailed in a specific Safety Monitoring Plan.

5.2.4.6. Sponsor emergency contact procedure

In the case of a medical emergency, contact Sponsor personnel shown below:

First line contact:

- **Regional Medical Monitor Asia -** Dr. George Watt
  Office: +66 2 263 2300 ext 139

Cell +6681 814 1032

Home: +66 2 966 8001

Email: gwatt@fhibkk.org

- **Central Medical Monitor – Dr Robert Miller** (who should be called in all cases of life-threatening events or events that result in death)

  Office: ++44 870 710 4360

  Cell +44 870 710 4360

  Fax: +44 870 710 4360

  Email: robert.miller@fulcrumpharma.com

5.2.4.7. Procedures in case of medical emergency

The principal Investigator(s) is responsible for ensuring that procedures and expertise are available to cope with medical emergencies during the study.

5.2.4.8. Procedures in case of overdose

The treatment of AEs associated with overdose should be supportive and for the underlying adverse symptoms. To date, no patient has experienced an overdose with the pyronaridine artesunate fixed combination.

Information on overdoses that do not result in AEs should be forwarded to the responsible CRA. This will then be reported to the Medical Monitor in accordance with procedures defined for non-SAEs.

5.2.4.9. Emergency procedures

Emergency equipment and drugs will be available within the clinical unit and their use in the context of the study will be documented on the CRF.

5.2.4.10. Follow-up of patients

Patients experiencing adverse events at D42 will be followed for a minimum of 30 days after the end of study or until resolution of the event, whichever is the shorter. A serious adverse event will be followed up to resolution or until no new medical information can be expected.

6. SCHEDULE OF STUDY PROCEDURES

The Study Plan is provided in Attachment 2.

Patients presenting at the clinical study site with potential compatibility with study inclusion/exclusion criteria will be considered for this clinical study. Prior to any study procedure, informed consent should be obtained from patient and/or parent/guardian/spouse, in accordance with local practice. If the patient is unable to write, witnessed consent is permitted according to local ethical considerations. On provision of consent the patient shall undergo the following:

	**Procedures**
**Screen and Pre-Dose (Baseline)**	**All assessments required by Inclusion and Exclusion criteria including:**<br>➢ Informed Consent/assent<br>➢ Clinical assessment<br>➢ Medical history, demography, contact and home details for follow up<br>➢ Physical examination including weight and height<br>➢ Vital signs: BP and HR<br>➢ Axillary, oral, tympanic or rectal temperature recorded<br>➢ Clinical signs and symptoms of malaria<br>➢ 12-lead ECG<br>➢ Haematology<br>➢ Blood biochemistry and urinalysis<br>➢ Thick and thin blood films for parasitological assessment in relation to confirmation of diagnosis of *P. vivax* malaria. Asexual parasites and gametocytes counts are recorded separately.<br>➢ G-6-PD testing<br>➢ Presence of antimalarials (urine tests: Saker Solomon and Lignin), if feasible<br>➢ Urine pregnancy test for female patients of child-bearing potential

**Patient Inclusion**	If Inclusion and Exclusion criteria are satisfied:<br><br>➢ Thick and thin blood films should be examined on Day 0 to confirm Inclusion / Exclusion criteria.<br>➢ Sample for PCR prior to dosing<br>➢ Concomitant medication recording<br><br>**Patient Inclusion and dosing (first dose D0)**
**First Dose Administered: D0**	➢ Body temperature recorded 8-hourly<br>➢ Thick blood films (2) prepared 8-hourly<br>➢ Administration of study medication Day 0 (first dose)<br>➢ Adverse events recording<br>➢ Concomitant medication recording
**Second Dose Administered: D1**	➢ Sample for PCR (prior to dosing)<br>➢ Administration of study medication Day 1 (second dose)<br>➢ Vital signs: BP and HR<br>➢ Temperature recorded 8-hourly<br>➢ Clinical signs and symptoms of malaria<br>➢ Thick blood films (2) prepared 8-hourly<br>➢ Adverse events recording<br>➢ Concomitant medication recording
**Third Dose Administered: D2**	➢ Administration of study medication Day 2 (third dose)<br>➢ Vital signs: BP and HR<br>➢ Temperature recorded 8-hourly<br>➢ Clinical signs and symptoms of malaria<br>➢ 12-lead ECG (2-4 hours post dose)<br>➢ Thick blood films (2) prepared 8-hourly<br>➢ Adverse events recording<br>➢ Concomitant medication recording
**Assessment D3**	➢ Physical examination<br>➢ Vital signs: BP and HR<br>➢ Temperature recorded 8-hourly<br>➢ Clinical signs and symptoms of malaria<br>➢ Haematology

	➢ Blood biochemistry and urinalysis<br>➢ Thick blood films (2) prepared 8-hourly<br>➢ Adverse events recording<br>➢ Concomitant medication recording<br>➢ Patient scheduled for follow up visits on Days 7, 14, 21, 28, 35 and 42<br>➢ Patient may be discharged from study site if parasite- and fever-free, with instructions to return in case of any recurrence of symptoms<br><br>note: If parasites and fever have not cleared on Day 3, the patient should be followed up until total clearance of parasites. If parasitaemia remains significant, the patient should be viewed as a treatment failure and withdrawn from the study. All End of Study Assessments (Day 28) should be performed before discharge and before rescue medication is given.<br><br>➢ Patient provided with an appropriate bed net
**Assessment D7 (+/- 1 day)**	➢ Vital signs: BP and HR<br>➢ Temperature recorded<br>➢ Clinical signs and symptoms of malaria (if clinically indicated)<br>➢ 12-lead ECG (if clinically indicated)<br>➢ Haematology<br>➢ Blood biochemistry and urinalysis<br>➢ Thick blood films (2) for asexual parasites and gametocytes counts (the total count is recorded)<br>➢ Thin blood film (as a reserve)<br>➢ Sample for PCR (as a reserve)<br>➢ Adverse events recording<br>➢ Concomitant medication recording
**Assessment D14 (window + 1 day)**	➢ Vital signs: BP and HR<br>➢ Temperature recorded<br>➢ Clinical signs and symptoms of malaria (if clinically indicated)<br>➢ 12-lead ECG (if clinically indicated)<br>➢ Thick blood films (2) for asexual parasites and gametocytes counts (the total count is recorded)<br>➢ Thin blood film (as a reserve)

	➢ Sample for PCR (as a reserve)<br>➢ hCG test for pregnancy in female patients of child-bearing potential<br>➢ Adverse events recording<br>➢ Concomitant medication recording
**Assessment D21 (window +/- 1 day)**	➢ Vital signs: BP and HR<br>➢ Temperature recorded<br>➢ Clinical signs and symptoms of malaria (if clinically indicated)<br>➢ Thick blood films (2) for asexual parasites and gametocytes counts (the total count is recorded)<br>➢ Thin blood film (as a reserve)<br>➢ Sample for PCR (as a reserve)<br>➢ Adverse events recording<br>➢ Concomitant medication recording
**Assessment D28 (window +1 days)**<br><br>**Or**<br><br>**Day of Treatment Failure**	➢ Physical examination<br>➢ Weight<br>➢ Vital signs: BP and HR<br>➢ Temperature recorded<br>➢ Clinical signs and symptoms of malaria (if clinically indicated)<br>➢ 12-lead ECG<br>➢ Haematology<br>➢ Biochemistry and urinalysis<br>➢ Thick blood films (2) for asexual parasites and gametocytes counts (the total count is recorded)<br>➢ Thin blood film (as a reserve and in case of re-appearing parasitaemia)<br>➢ Sample for PCR<br>➢ Urine hCG test for pregnancy in female patients of child bearing potential<br>➢ Adverse events recording<br>➢ Concomitant medication recording<br>➢ Rescue therapy in case of treatment failure<br>➢ Pyronaridine blood levels in case of treatment failure<br>➢ **For patients with normal G-6-PD activity, oral primaquine treatment (once daily for 14 days) to be started AFTER all**

	**assessments have been performed. For women of childbearing potential, primaquine should only be started once a negative pregnancy test is demonstrated.**
**Assessment D35**<br><br>**(window +/- 2 day)**	➢ Vital signs: BP and HR<br>➢ Temperature recorded<br>➢ Clinical signs and symptoms of malaria (if clinically indicated)<br>➢ Thick blood films (2) for asexual and gametocytes parasite counts (the total count is recorded)<br>➢ Thin blood film (as a reserve)<br>➢ Sample for PCR (as a reserve)<br>➢ Adverse events recording<br>➢ Concomitant medication recording
**Assessment D42**<br>**(window +2 day)**	➢ Physical examination<br>➢ Vital signs: BP and HR<br>➢ Temperature recorded<br>➢ Clinical signs and symptoms of malaria (if clinically indicated)<br>➢ 12-lead ECG (if clinically indicated)<br>➢ Haematology<br>➢ Blood biochemistry and urinalysis (only if clinically significant abnormalities have been observed)<br>➢ Thick blood films (2) for asexual and gametocytes parasite counts (the total count is recorded)<br>➢ Thin blood film (as a reserve)<br>➢ PCR sample (as a reserve)<br>➢ Urine hCG test for pregnancy in female patients of child bearing potential<br>➢ Adverse events recording<br>➢ Concomitant medication recording
**Any day with fever or unscheduled visit**	➢ Temperature recorded<br>➢ Clinical signs and symptoms of malaria<br>➢ Thick (2) blood films for asexual and gametocytes parasite counts (the total count is recorded)<br>➢ Thin blood film in case of re-appearing parasitaemia<br>➢ Sample for PCR in case of re-appearing parasitaemia

	➢ Blood sample for pyronaridine blood levels in case of re-appearing parasitemia<br>➢ Rescue therapy in case of treatment failure<br>➢ Adverse events recording<br>➢ Concomitant medication recording
**Population PK**<br><br>**(pyronaridine and artesunate blood levels)**	At **selected** clinical study sites, **as determined by the Sponsor**, the Investigator is required to collect **samples as follows**:<br><br>➢ **Sampling for Pyronaridine Population Pharmacokinetics: 2 blood samples from each patient at two different timepoints. One sample will be drawn between Day 0 (post dose) and Day 3, and a second between Day 4 and Day 42.**<br>➢ **Sampling for Artesunate Population Pharmacokinetics: 2 plasma samples from each patient at two different timepoints. One sample will be drawn on Day 0 (post-dose) or Day 2, and a second one on Day 3.**<br><br>A list of timepoints will be generated corresponding to each patient number and provided to the Principal Investigator. Actual time must be recorded.<br><br>Additionally, **at all sites**, a blood sample must be drawn for assay of pyronaridine (in whole blood) at time of treatment failure.

7. STUDY ADMINISTRATION

7.1. Monitoring

Study monitors will contact and visit the Investigator regularly and will be allowed, on request, to inspect the various records of the study provided that patient confidentiality is maintained in accord with local requirements.

Before the initiation of the study, a representative of the Sponsor will visit the investigational site to:

- Determine the adequacy of the facilities in the context of malaria endemic countries
- Discuss with the Investigator(s) (and other personnel involved with the study) their responsibilities with regard to protocol adherence, and the responsibilities of Sponsor or its representatives

During the study, the monitor or another representative of the Sponsor will have regular contacts with the investigational site, including visits to:

- Provide information and support to the Investigator(s)
- Confirm that facilities and equipment remain acceptable
- Confirm that the investigational team is adhering to the protocol, that data are being accurately recorded in the CRFs, and that investigational product accountability checks are being performed
- Perform source data verification (a comparison of the data in the CRFs with the source records relevant to the study). This will require direct access to all original records for each patient (e.g. clinic charts, laboratory test reports) where relevant. A description of source data will be described in the monitoring plan.

The monitor or another representative of the Sponsor will be available between visits if the Investigator(s) or other staff at the centre needs information and advice.

The Investigator shall agree to co-operate with the study monitor to ensure that any problems detected in the course of these monitoring visits are resolved in a timely manner.

7.2. Special Consideration of Safety Monitoring

7.2.1. Medical monitor responsibilities

The Study Medical Monitor will be responsible for the evaluation of safety aspects of the study. This includes the review of available information from the regional medical monitors and the weekly status. Furthermore, the medical monitor would review and assess all reports of SAEs and determine whether these events must be reported to the competent authorities.

7.2.2. Sponsor responsibilities

Sponsors' representatives will promptly notify all concerned Investigators, IEC and the regulatory authorities of findings that could adversely affect the safety of patients, impact the conduct of the study, or alter the IEC's approval/favourable opinion to continue the study. In addition, Sponsors' representatives will expedite the reporting to all concerned Investigators/institutions, to the IEC and to the regulatory authorities of all adverse drug reactions that are both **serious and unexpected**.

7.3. Audits and Inspections

Authorised representatives of Sponsor, a regulatory authority, an IEC or an IRB may visit the centre to perform audits or inspections, including source data verification. The purpose of a Sponsor audit or inspection is to systematically and independently examine all study-related activities and documents to determine whether these activities were conducted, and whether data were recorded, analysed and accurately reported according to the protocol, Good Clinical Practice (GCP), guidelines of the International Conference on Harmonisation (ICH), and any applicable local regulatory requirements. The Investigator should contact Sponsor immediately if they are contacted by a regulatory agency about an inspection at their centre.

7.4. Training of Staff

The principal Investigator will maintain a record of all individuals involved in the study (medical, nursing and other staff). He or she will ensure that appropriate training relevant to the study is given to all these staff, and that they will receive any new information of relevance to the performance of this study.

7.5 Changes to the Protocol

Study procedures will not be changed without the mutual agreement of the Investigator(s) and Sponsors.

If it is necessary for the study protocol to be amended, the amendment or a new version of the study protocol must be notified to or approved by each IEC or IRB, and in many countries also the local regulatory authority, before implementation. Local requirements must be followed.

If a protocol amendment requires a change to a particular centre's Informed Consent Form, Sponsor and the centre's IEC or IRB must be notified. Approval of the revised Informed Consent Form by Sponsor and by the IEC or IRB is required before the revised form can be used.

Sponsor will distribute amendments and new versions of the protocol to each principal Investigator(s), who in turn is responsible for the distribution of these documents to his or her IEC or IRB, and to the staff at his or her centre. The distribution of these documents to the regulatory authority will be handled according to local practice.

7.6. Data Handling and Record Keeping

CRFs will be provided for each patient. Patients will be identified by the patient identification number and the study identification number on all study documents.

All data on the CRF must be legibly recorded in black ink or typed. A correction should be made by drawing a single line through the incorrect entry and entering the correct information adjacent to it. The correction must be initialed and dated by the Investigator or a designated, qualified individual. Any requested information that is not obtained as specified in the protocol should have an explanation noted on the CRF as to why the required information was not obtained.

The Investigator must maintain adequate and accurate records to enable the conduct of the study to be fully documented and the study data to be subsequently verified. These documents should be classified into two separate categories:

- The Investigator's study file.
- The patient's clinical source documents.

The Investigator's study file will contain the protocol, amendments, CRF and query form, independent EC and regulatory approval together with the relevant correspondence, example of the patient informed consent form and patient information sheet, drug records, staff curricula vitae and authorization forms and other appropriate documents or correspondence.

Patient clinical source documents (usually defined by the project in advance to record key efficacy or safety parameters independent of the CRFs) would include patient hospital or clinic records, physician's and nurse's notes, appointment book, original laboratory reports, ECG, pathology and special assessment reports, signed informed consent forms, consultant letters, and patient screening and enrollment logs.

All study-related documents must be kept on file by the Investigator until at least 15 years after the last approval of a marketing application in an ICH region and until there are no pending or contemplated marketing applications in an ICH region or at least 15 years have elapsed since the formal discontinuation of clinical development of the investigational product. However, these documents should be maintained for a longer period if required by local applicable regulatory requirements. No documents should be destroyed without prior written agreement between the Sponsor and the Investigator. Should the Investigator wish to assign the documents to another party or move them to another location, the Sponsor must be notified in writing before the documents are reassigned or moved.

If the Investigator cannot guarantee the archiving requirement at the investigational center for any or all of the documents, special arrangements must be made between the Investigator and the Sponsor to store these in a sealed container(s) away from the center so that they can be returned sealed to the Investigator in case of a regulatory audit. Where source documents are required for the continued care of the patients, appropriate copies should be made prior to storing away from the center.

The Sponsor reserves the right to terminate the study at any time. Should this be necessary, both the Sponsor and the Investigator will arrange the procedures on an individual study basis after review and consultation. In terminating the study,

the Sponsor and the Investigator will assure that adequate consideration is given to the protection of the patient's interests.

7.7. Patient Confidentiality

The CRF as well as all reports and communications relating to patients in the study will identify each patient only by the patient's initials (first, middle, last) and by the patient identification number. The Investigator will maintain a current confidential Patient Identification Code List of names of all patients allocated to patient identification numbers in this study. This list will allow the Investigator to reveal the identity of the patients in the event that they need to be contacted for safety reasons. This information will be held in the strictest confidence and will only be used for emergency purposes, if needed.

All CRF pages are used to transmit the information collected in the performance of this study to the Sponsor and to governmental agencies, as appropriate. All pages of the CRF must be completed for each patient who receives any amount of study medication.

Portions of the patient's medical records pertinent to the study will be reviewed by the Sponsor personnel or their designee and possibly by regulatory authority personnel to assure adequate source documentation, accuracy, and completeness of the CRFs.

7.8. Direct Access to Source Data/Documents

Upon request, the Investigator shall supply the Sponsor with any required background data from the study documentation or clinic records. This is particularly important when CRFs are illegible or when errors in data transcription are suspected. In case of special problems and/or governmental queries or requests for audit inspections, it is also necessary to have access to the complete study records, provided that patient confidentiality is protected.

FHI will be responsible for the clinical monitoring of the study. FHI clinical research assistants (CRAs) will visit the participating center to review the research records for accuracy, completeness and legibility.

The Investigator will make study documents (for example, consent forms, treatment forms, CRFs) and pertinent hospital/clinic or clinic records readily available for inspection by the study monitor for confirmation of the study data.

7.9. Investigator Information

7.9.1. Protocol approval and signature

After reading the protocol, the Investigator will sign two protocol signature pages and return one of the signed pages to the Sponsor.

7.9.2. Study agreements

The principal Investigator and all study staff must comply with all the terms, conditions and obligations of the study agreement for this study.

7.9.3. Publication policy

All information concerning pyronaridine artesunate and the Sponsor's operations, such as the Sponsor patent applications, formulas, manufacturing processes, basic scientific data, or formulation information, supplied by the Sponsor and not previously published is considered confidential information.

The information developed during the conduct of this clinical study is also considered confidential and will be used by the Sponsor in connection with the development of pyronaridine artesunate. This information may be disclosed as deemed necessary by the Sponsor. To allow for the use of the information derived from this clinical study and to ensure complete and thorough analysis, the Investigator is obligated to provide the Sponsor with complete test results and all data developed in this study.

This confidential information shall remain the sole property of the Sponsor, shall not be disclosed to others without the written consent of the Sponsor, and shall not be used except in the performance of this study.

The Sponsor encourages publication of study results. However no study data shall be disclosed prior to the final analysis of the data. Should an Investigator wish to publish results from this study, a copy of the manuscript must be provided to the Sponsor at least 30 days before the date of submission to the intended publisher.

7.10 Financing and Insurance

This study is to be conducted in accordance with the standards stipulated in this protocol and Clinical Trial Agreement. The Sponsor, Shin Poong Pharm Co Ltd. and Medicines for Malaria Venture, will indemnify the Principal Investigator, co-Investigators, and other relevant parties from all and any claims arising from this study with the exception of claims resulting from their negligence, malpractice or

violation of the protocol.

In the event that it can be demonstrated that a patient suffers any significant deterioration in health or well-being or any harmful susceptibility or toxicity as a direct result of their participation in this study then Shin Poong Pharm Co Ltd and Medicines for Malaria Venture will agree to abide by International Guidelines and local regulations with regards to the compensation payable to the patient. The amount of compensation will be calculated by reference to the cost of best standard care provided by the local health system for similar injuries at the time when such injury occurred.

7.11. Study Timetable and Termination

The planned start date for this study is Q4 2006. It is anticipated that accrual will take up to approximately 5 months per site and the proposed patient completion date is estimated as Q2 2007.

8. DATA MANAGEMENT

The Data Management will be performed by an independent contract research organisation (CRO).

The top, white, no carbon required (NCR) copy of each CRF will be sent to the data management group for the study. The middle copy of the CRF will be retained by the monitoring CRA and the bottom copy will be retained by the Investigator. On receipt at the CRO, the CRFs will be logged into an electronic tracking system. The data will be entered onto a database using blind double data entry. Where the data violate validation checks, queries will be generated via Data Clarification Forms (DCFs). These forms may be generated as three-part NCR paper that is colour-coded to match the CRF, or as electronic files. DCFs will be sent to the monitoring CRA for resolution by the Investigator. On receipt of the original resolution at the CRO (a copy retained by the CRA and the Investigator), the database will be edited appropriately. Information on the status of each data clarification form (DCF) will be maintained in an electronic tracking system within the database. All edits made to the database on resolution of the DCFs will be electronically audit-trailed and 100% quality control (QC) checked.

Medications will be coded using the WHO-drug dictionary. Medical conditions and AEs will be coded the MedDRA dictionary.

When data entry has been completed and all outstanding queries have been resolved for a CRF, it will be eligible for QC checking. A √n +1sample of patients will be selected for QC checks and 100% of these data will be audited. In addition, 100% of demography, AEs and withdrawal data will be checked for all patients. The highest acceptable error rate for QC is 0.5%. A higher error rate will prompt a review of a further sample of √n +1% of patients. If the error rate remains above 0.5%, a 100% QC check of all data will be conducted. All errors discovered will be corrected on the database and the corrections will be audit-trailed and will undergo a QC check.

Before database lock, a further √n +1% sample of patients will be selected for quality assurance (QA) checks. The highest acceptable error rate for QA is 0.5%. A higher error rate will prompt the same actions as for QC. All errors discovered on the database will be corrected and the corrections will be audit-trailed. On satisfactory completion of the QC and QA checks, the database will be locked.

9. STATISTICAL METHODS

The statistical methods for this study, summarised below, will be described in a detailed statistical analysis plan (SAP) which will be finalised and approved before database lock. Any deviations from the planned analysis as described below or in the SAP will be justified and recorded in the final study report.

9.1. General Considerations

Data will be summarised as follows: Continuous variables by descriptive statistics (number of patients [n], mean, standard deviation [SD], minimum, median, maximum); categorical data by absolute and relative frequencies (n and %) or contingency tables.

Unless indicated otherwise, summary statistics will be reported for observed data only. Missing data will not be imputed. If a baseline value is missing, no change from baseline will be calculated. Baseline is defined as the last observation prior to the first administration of study medication.

9.2. Analysis Populations

Safety Population (Safety)

The Safety population will consist of all randomised patients who have received any amount of study medication. Patients will be analysed as treated.

Intent-To-Treat Population (ITT)

The Intent-to-Treat (ITT) population will consist of all randomised patients who have received any amount of study medication. Patients will be analysed as randomized.

Efficacy Evaluable (EE) Population

The efficacy evaluable (EE) population will include those patients in the ITT population meeting pre-defined criteria as follows:

1. Having completed a full course of study medication and having known efficacy endpoints.
2. No missed dose due to vomiting, as recorded on the ‘study medication administration page’ of the CRF, except at Day 0 where a patient may vomit after the first drug administration and have a repeat full dose. In this case, the repeated dose should not be vomited.

3. No concomitant medication (except paracetamol) which may interfere with the treatment outcome, up to Day 14. The list of forbidden medication is given in Attachment 5. A medical review will be performed to confirm the exclusion of a patient from the EE population.
4. No concomitant disease, such as Tuberculosis, HIV, severe URTI, gastrointestinal disease, etc…, which may interfere with the clear classification of the treatment outcome. A medical review of the experienced diseases will be performed to define the list of patients excluded from the EE population.
5. No major protocol violation. The list of all protocol violations will be assessed with regards to their impact (major/minor) prior to database lock.

The list of patients excluded from the EE population will be reviewed and approved prior to study unblinding and database lock.

9.3. Patient Disposition

The Investigator will be responsible for maintaining a screen log and reason for non inclusion will be recorded. This will be maintained and will be provided to the Sponsor at regular intervals throughout the study and the totality at the end of the study.

The number of patients recruited and receiving study drug will be presented by treatment group, as will be the number of patients included in the various analysis populations.

Protocol violations will be listed per patient, describing the nature of the violation and specifying whether it is a major or a minor violation.

9.4. Determination of Sample Size

Assuming a cure rate on Day 14 of 90% in both treatment groups and assuming a non-inferiority limit of -10%, then a sample size of 410 evaluable patients randomized in 1:1 ratio (205 patients in each treatment group) would provide 90% power to demonstrate non-inferiority of PA compared to the chloroquine, using a two-sided 95% confidence interval with Normal approximation. Assuming a drop out rate of 10%, 456 subjects would be randomized to the study (228 subjects in each treatment group).

Assuming a cure rate on Day 14 of 95%, this sample size would provide >99% power to demonstrate non-inferiority of PA compared to the chloroquine.

Sample size estimation was performed using nQuery statistical software version 4.0.

9.5. Statistical Evaluation

9.5.1. Efficacy and safety endpoints

Patient outcome will be classified according to WHO "Monitoring antimalarial drug resistance- Report of a WHO consultation Geneva, Switzerland, 3-5 December 2001".

Primary Efficacy Endpoint

The primary efficacy endpoint for the study is the cure rate on Day 14.

Cure on Day 14 will be defined as no parasitemia on Day 14 irrespective of axillary temperature without previously meeting any of the criteria of treatment failure throughout the follow-up period.

Treatment failure is defined as any of the following:

- Clinical deterioration due to *P. vivax* illness requiring hospitalization in presence of parasitemia.
- Presence of parasitemia and axillary temperature ≥37.5°C any time between Day 3 and Day 28.
- Presence of parasitemia on any day between Day 7 and Day 28 irrespective of clinical condition.

Patients presenting a *P. falciparum* infection after clearance of *P. vivax* parasites should be treated accordingly and withdrawn. They should not be considered as *P. vivax* treatment failures.

Secondary Efficacy Endpoints

The secondary efficacy endpoints are:

- Cure rate on Day 21 and Day 28, defined in the same way as cure rate on Day 14.
- Parasite Clearance Time (PCT).

  Parasite clearance time is defined as the time from first dosing to time of first blood draw with parasite clearance. Parasite clearance

is defined as zero presence of asexual parasites for two consecutive negative readings taken between eight and twenty-four hours apart.

- Fever Clearance Time (FCT)

  Fever clearance time is defined as the time from first dosing to first normal reading of temperature (<37.5 °C taken axillary or tympanic) for two consecutive normal temperature readings taken between eight and twenty-four hours apart.

  The method of temperature measurement must be the same (i.e. axillary, tympanic, oral or rectal) for all measures in the same patient. Those patients who are initially included on history of fever and who do not subsequently have a documented temperature reading of over 37.5 °C (taken axillary or tympanic) during the 24 hours following initial dosing, will not be included in this endpoint analysis.

- Proportion of patients who have cleared parasites on Day 1, 2 and 3
- Proportion of patients who have cleared fever on Day 1, 2 and 3

Safety endpoints:

- Incidence of any adverse events or clinically significant laboratory abnormalities
- Vital signs, new physical examination abnormalities
- ECG abnormalities

Exploratory Endpoints

The exploratory endpoints are:

- Proportion of patients with PCR-corrected cure on Day 14
- Proportion of patients with PCR-corrected cure on Day 28
- Cure rate on Day 42
- Proportion of patients with PCR-corrected cure on Day 42

Genotyping of *P. falciparum*-specific polymorphic genes by PCR is well established to distinguish recrudescence from new infection. However, in the case

of *P. vivax*, polymorphic molecular markers have not been fully validated. Current molecular tools may identify new infection (or relapse from a previous infection) but cannot distinguish relapse (of current infection) from recrudescence. Endpoints determined by PCR analysis will be regarded as exploratory.

New infection as evidenced by PCR-genotyping studies will be considered as treatment success.

Due to the lack of validated molecular tools, the primary endpoint will be based on a crude cure rate on Day 14. However, the Day 28 data will be collected and analysed as secondary endpoint.

9.5.2. Methods of statistical analysis

A detailed statistical analysis plan will be prepared and documented prior to study unblinding and database lock.

Primary Efficacy Analysis:

The primary efficacy analysis will test the non-inferiority of the PA group compared to the chloroquine group with regard to cure rate on Day 14 using a two sided 95% confidence interval and a 10% non-inferiority margin and be performed on the efficacy evaluable (EE) population. Non-inferiority will be demonstrated if the lower limit of the two-sided 95% confidence interval (CI) for the difference in cure rates on Day 14 (normal approximation) is not lower than -10%.

The following primary efficacy hypothesis will be tested:

H_0: The cure rate on Day 14 for the PA group is inferior to the cure rate on Day 14 for the comparator group by more than 10%.

Versus the alternative

H_A: The cure rate on Day 14 for the PA group is NOT inferior to the cure rate on Day 28 for the comparator group by more than 10%.

Secondary Efficacy Analyses:

The primary efficacy analysis performed on Day 14 will be repeated for the proportion of patients with cure on Day 21 and 28, using the efficacy evaluable population.

In addition, the analysis performed using the EE population will be repeated using the ITT population.

The parasite clearance time and fever clearance time will be analysed using Kaplan-Meier estimates. The median and quartiles clearance time will be presented together with the corresponding 95% confidence intervals. The mean will also be calculated. The survival curves will be presented .The log rank test will be used to compare the parasite clearance time and fever clearance time between the two treatment groups. Patients who do not have documented parasite or fever clearance will be censored. Censoring time will be greather than the highest observed clearance time.

The proportion of patients who have parasite clearance and the proportion of patients who have fever clearance will be presented at Day 1, Day 2 and Day 3. The associated exact 95% confidence interval will also be calculated.

The number of parasites will be summarized using standard descriptive statistics, as well as geometric mean and its associated 95% confidence interval using an analysis of variance model on $\log_{10}$ transformed parasites counts with treatment and site as factors. The mean parasite count at each scheduled timepoint will be presented graphically.

All secondary efficacy analyses will be performed both on the EE and ITT populations.

Exploratory Analyses:

The primary efficacy analysis performed on Day 14 will be repeated for the proportion of patients with PCR-corrected cure on Day 14 and 28, using the efficacy evaluable population and the ITT population.

Safety Analysis

Safety analyses will be based on the Safety population.

Adverse Events

Adverse events (AEs) will be summarized by presenting, for each treatment

group, the number and percentage of patients having any AE, having an AE in each system organ class and having each individual AE (preferred term). Additionally, a summary of adverse events by preferred term and severity will be performed using the worst severity grade.

All related AEs, AEs with outcome death, AEs leading to permanent discontinuation of treatment, SAEs and related SAEs will be summarized by percentages and frequencies and listed including the Investigator term, the preferred term, start and end date of AE, duration (days), severity, drug relationship, action taken and outcome.

Treatment related AEs are those rated by the Investigator as “definite”, “probable”, “possible”. In case relationship is unknown or missing the worst case will be assumed and the AE will be considered to be drug related.

Adverse events will be coded using the latest available MedDRA dictionary version.

Laboratory Data

The incidence of patients with a clinically significant abnormal value as reported by the Investigator will be provided by lab parameter, treatment group and scheduled visit.

For each laboratory parameter, a summary of shift in abnormalities from baseline to each post-baseline visit will be presented. Normal ranges will be used to determine if a laboratory value was normal or not.

Summary statistics will be computed for all safety laboratory parameters. All laboratory values will be converted to SI units.

Vital Signs

Supine blood pressure and heart rate (absolute values and change from baseline) will be summarized by treatment groups and scheduled visit.

ECG

The incidence of patients with a clinically significant abnormal ECG result will be provided.

A summary of shifts in 12-lead ECG abnormalities from baseline will be performed by scheduled visits.

Physical Examination

Physical examination assessments will be summarised by visit.

9.5.3. Interim analyses

A blind interim analysis will be performed when 50% of the patients have reached the Day 14 timepoint to confirm the expected cure rate. The sample size may be re-assessed if the observed cure rate is lower than expected.

10. ETHICS

10.1. Ethics Review

The Investigator must submit to his/her local Ethics Committee the following documents: Investigator Brochure, signed protocol, informed consent and any other material relevant to the study such as advertising, in line with local regulations. The final study protocol, including the final version of the Informed Consent Form, must be approved or given a favourable opinion in writing by a local Ethics Committee. The Investigator must provide written documentation to the effect that the Ethics Committee has given favourable opinion to the protocol and informed consent. The Ethics Committee must also acknowledge having reviewed the current Investigator Brochure. Final approval must be provided to the Sponsor before any patient can be enrolled into the study. A membership list of the Ethics Committee must be provided to Sponsor before commencement of the study, using the form provided by MMV (see Attachment 10).

The principal Investigator(s) is responsible for informing the local Ethics Committee (EC) of any amendment to the protocol in accordance with local requirements. The protocol must be re-approved by the EC annually, as local regulations require.

Either the Investigator(s) or Sponsor must submit progress reports to the EC according to local regulations and guidelines. The principal Investigator(s) must also provide the EC with any reports of SAEs from the study site.

10.2. Ethical Conduct of the Study

This study will be conducted in compliance with the study protocol. The patient's informed consent will be obtained according to the ethical principles stated in the Declaration of Helsinki 2000 version (amended in Tokyo 2004) (see Attachment 8), the applicable guidelines for ICH GCP, and the applicable laws and regulations of the participating countries.

10.3. Patient Information and Consent

The informed consent document will be used to explain the risks and benefits of study participation to the parent or guardian of the patient in simple terms before the patient will be entered into the study. The informed consent document

contains a statement that the consent is freely given, that the parent or guardian of the patient is aware of the risks and benefits of entering the study, and that the patient is free to withdraw from the study at any time. Written consent must be given by the legal representative, after the receipt of detailed information on the study. Where appropriate, children will be asked to provide assent.

The Investigator is responsible for ensuring that informed consent is obtained from each legal representative and for obtaining the appropriate signatures and dates on the informed consent document prior to the performance of any protocol procedures and prior to the administration of study medication. A copy of the signed and dated informed consent form will be offered to the patient's parent or guardian. The signed and dated original consent form will be retained with the study records. Patients cannot undergo study-related procedures until the informed consent form has been signed and dated. Pertinent dosing information, scheduled visit dates, and instruction for contacting the Investigator will be on the study medication package or the consent form, as appropriate. The draft informed consent should be provided for review to the Sponsor prior submission to the Ethical Committee. The elements which should appear in the informed consent form are available in Attachment 9. If modifications are made according to local requirements, any new version has to be approved by Sponsor.

Given that some patients for inclusion in this study may not be capable of giving legal consent, written consent must be obtained from next of kin. In the case where the next of kin are unable to read, an impartial witness should be present during the entire informed consent discussion. After the next of kin has orally consented to participate in the study, the witness's signature on the form will attest that the information in the informed consent form and patient information sheet was accurately explained and understood. In case of a parent or guardian of the patient who cannot provide informed consent in writing, a thumbprint to indicate consent in the presence of two witnesses is acceptable. Permission from the parent or guardian must be obtained in the case of assenting minors.

The CRFs for this study will contain a section for documenting the date of patient informed consent and this must be completed appropriately. If new safety information results in significant changes in the risk/benefit assessment, the consent form and patient information sheet should be reviewed and updated if necessary. All parents or guardians of patients (including those already being treated) should be informed of the new information, given a copy of the revised

consent form and give their consent or assent to continue the study.

The principal Investigator(s) at each centre will ensure that the parent or guardian of the patient is given full and adequate oral and written information about the nature, purpose, possible risk and benefit of the study. Patients and their parent or guardian must also be notified that they are free to discontinue from the study at any time. The patient and parent or guardian should be given the opportunity to ask questions and should be allowed time to consider the information provided.

10.4. Patient Data Protection

The Informed Consent Form will explain that study data will be stored in a computer database, maintaining confidentiality in accordance with GCP. Patients in this database will be identified by initials or patient number only. The Informed Consent Form will also explain that for data verification purposes, authorised representatives of Sponsor, a regulatory authority, an EC may require direct access to parts of the hospital or practice records relevant to the study, including patient medical history.

11. REFERENCES

Alecrim MdGC, Carvalho LM, Fernandes MC et al. (2000) Malaria treatment with artesunate (retocapsR) in children of the Brazilian Amazon. Rev. Soc. Bras. Med. Trop. 33:163-8.

Baird JK, Basri H, Purnomo, Bangs MJ, Subianto B, Patchen LC and Hoffman SL (1991) Resistance to chloroquine by Plasmodium vivax in Irian Jaya, Indonesia. Am J Trop Med Hyg 44: 547-552.

Baird JK, Sustriayu Nalim MF, Basri H, Masbar S, Leksana B, Tjitra E, Dewi RM, Khairani M, and Wignall FS (1996) Survey of resistance to chloroquine by Plasmodium vivax in Indonesia. Am J Trop Med Hyg 90: 409-411.

Baird JK (2004) Chloroquine resistance in Plasmodium vivax. Antimicrob Agents Chemother 48: 4075-4083.

Batty K, Thu LTA, Ilett KF, Tien NP, Powell SM, Hung NC, Mai TX, Chon VV, Thien HV, Binh TQ, Van Kim N and Davis TME (1998) A pharmacokinetic and pharmacodynamic study of artesunate for vivax malaria. Am J Trop Med Hyg 59: 823-827.

Chai JY (1999) Re-emerging Plasmodium vivax malaria in the Republic of Korea. Korean J Parasitol 37: 129-143.

Chen C, Tang, LH and Jantanavivat C (1992) Studies on a new antimalarial compound: pyronaridine. Trans R Soc Trop Med Hyg 1992; 86: 7-10.

de Vries PJ and Dien TK (1996) Clinical pharmacology and therapeutic potential of artemisinin and its derivatives in the treatment of malaria. Drugs 52: 818-836.

Doberstyn E B, Teerakiartkamjorn C, Andre RG, Phintuyothin P, and Noeypatimanondh S (1979) Treatment of vivax malaria with sulfadoxine-pyrimethamine and with pyrimethamine alone. Trans Soc Trop Med Hyg 73: 15-17.

Dua VK, Kar PK and Sharma VP (1996) Chloroquine resistant Plasmodium vivax malaria in India. Trop Med Int Health 1: 816-819.

Duffy PE, Mutabingwa TK. Artemisinin combination therapies. Lancet 2006. 367:2037-9

Fu S, and Xiao SH (1991) Pyronaridine: A new antimalarial drug. Parasitology Today 7: 310-313.

Garg M, Gopinathan N, Bodhe P and Kshirsagar NA (1995) Vivax malaria resistant to chloroquine: case reports from Bombay. Trans R Soc Trop Med Hyg 89: 656-657.

Geng WM, Zhang JM, Chen C, Cui YP, Zeng YL and Gu LW (1989) Redical curative efficacy of pyronaridine combined with primquine to vivax malaria. Jiangsu Journal of Parasitic Diseases 4: 27-28.

Hamedi Y, Safa O, Zare S, Tan-ariya P, Kojima S and Looareesuwan S (2004) Therapeutic efficacy of artesunate in Plasmodium vivax malaria in Thailand. Southeast Asian J Trop Med Public Health 35: 570-574.

Hui BL, Shen SL, Wang KS, Gao SF and Cao SZ (1984) The radical efficacy of pyronaridine-primaquine combination in tertian malaria. Journal of Parasitology and Parasitic Diseases [Ji Sheng Chong Xue Yu Ji Sheng Chong Bing Za Zhi] 2: 260-262.

Imwong M, Pukrittakayamee S, Looareesuwan S, Pasvol G, Poirreiz J, White N J and Snounou G (2001) Association of genetic mutations in Plasmodium vivax dhfr with resistance to sulfadoxine-pyrimethamine: geographical and clinical correlates. Antimicrob Agents Chemother 45: 3122-3127.

Karunajeewa HA, Kemiki A, Alpers MP et al. (2003) Safety and therapeutic efficacy of artesunate suppositories for treatment of malaria in children in Papua New Guinea. Ped Inf Dis J 22:251-5.

Karunaweera ND, Wijesekera SK, Wanasekera D, Mendis KN and Carter R (2003) The paroxysm of Plasmodium vivax malaria. Trends in Parasitol 19: 188-193.

Kochar DK, Saxena V, Singh N, Kochar SK, Kumar SV and Das A (2005) Plasmodium vivax malaria. Emerg Infect Dis 11: 132-134.

Myat-Phone-Kyaw, Myint-Oo, Myint-Lwin, Thaw-Zin, Kyin-Hla-Aye and Nwe-Nwe-Yin (1993) Emergence of chloroquine-resistant Plasmodium vivax in Myanmar (Burma). Trans R Soc Trop Med Hyg 87: 687.

Krotoski WA, Collins WE, Bray RS, Garnham PC, Cogswell FB, Gwadz RW, Killick-Kendrick R, Wolf R, Sinden R, Koontz LC, Stanfill PS (1982) Demonstration of hypnozoites in sporozoite-transmitted Plasmodium vivax infection. Am J Trop Med Hyg 31:1291-1293.

Li GQ, Guo XB, Fu LC, Jian HX and Wang XH (1994) Clinical trials of artemisinin and its derivatives in the treatment of malaria in China. Trans R Soc Trop Med Hyg 88: S5-6.

Li X, Li C, Che L, Liu X, Li Z, Huang D, He W, and Dao Q (1999) Observation on efficacy of artemether compound against vivax malaria. Zhongguo Ji Sheng Chong Xue Yu Ji Sheng Chong Bing Za Zhi 17: 175-177.

Liu YL, Wan CC, He JJ, Hu YN, Jia JX, Jiang WK, Lo MZ and Shi WQ (1990) The efficacy of three therapeutic regimens in treating vivax malaria patients. Chinese Journal of Parasitic Disease Control 3: 267-270.

Looareesuwan S, Kyle DE, Viravan C, Vanijanonta S, Wilairatana P, and Werndorfer WH (1996) Clinical study of pyronaridine for the treatment of acute uncomplicated falciparum malaria in Thailand. Am J Trop Med Hyg 54: 205-209.

Marlar-Than, Myat-Phone-Kyaw, Aye-Yu-Soe, Khaing-Khaing-Gyi, Ma-Sabai and Myint-Oo (1995) Development of resistance to chloroquine by Plasmodium vivax in Myanmar. Trans R Soc Trop Med Hyg 89: 307-308.

Mayxay M, Pukrittayakamee S, Newton PN and White NJ (2004) Mixed-species malaria infections in humans. Trends in Parasitol 20: 233-240.

Mendis K, Sina BJ, Marchesini P and Carter R (2001) The neglected burden of Plasmodium vivax malaria. Am J Trop Med Hyg 64: 97-106.

Murphy GS, Basri H, Purnomo MS, Andersen EM, Bangs MJ, Gorden J, Sorensen K, Mount DL, Lal AA, Purwokusumo AR, Harjosuwarno S and Hoffman SL (1993) Vivax malaria resistant to treatment and prophylaxis with chloroquine. Lancet 341: 96-100.

Nacher M, Silachamroon U, Singhasivanon P, Wilairatana P, Phumratanaprapin W, Fontanet A and Looareesuwan S (2004) Comparison of artesunate and chloroquine activities against plasmodium vivax gametocytes. Antimicrob Agents Chemother 48: 2751-2752.

Oh MD, Shin H, Shin D, Kim U, Lee S, Kim N, Choi MH, Chai JY, and Choe K (2001) Clinical features of vivax malaria. Am J Trop Med Hyg 65: 143-146.

Phan GT, de Vries PJ, Tran BQ, Le HQ, Nguyen NV, Nguyen TV, Heisterkamp SH and Kager PA (2002) Artemisinin or chloroquine for blood stage Plasmodium vivax malaria in Vietnam. Tropical Medicine and International Health 7: 858-864.

Philips EJ, Keystone JS, and Kain KC (1996) Failure of combined chloroquine and high-dose primaquine therapy for P. vivax malaria acquired in Guyana, South America. Clinical Infectious Diseases 23:1171-1173.

Price R, Van Vugt M, Phaipun L et al: Adverse effects in patients with acute falciparum malaria treated with artemisinin derivatives. J Am J Trop Med Hyg 1999; 60 (4), 547-555.

Pukrittayakamee S, Chantra A, Simpson JA, Vanijanonta S, Clemens R, Looareesuwan S and White NJ (2000) Therapeutic responses to different antimalarial drugs in vivax malaria. Antimicrob Agents Chemother 44: 1680-1685.

Pukrittayakamee S, Imwong M, Looareesuwan S, and White NJ (2004) Therapeutic responses to antimalarial and antibacterial drugs in vivax malaria. Acta Tropica 89: 351-356.

Rieckmann KH, Davis DR, Hutton DC (1989) Plasmodium vivax resistance to chloroquine? Lancet 2: 1183-1184.

Ringwald P, Bickii J, and Basco L (1996) Randomised trial of pyronaridine versus chloroquine for acute uncomplicated falciparum malaria in Africa. Lancet 347: 24-28.

Ringwald P, Bickii J, Same-Ekobo A and Basco LK (1997) Pyronaridine for Treatment of Plasmodium ovale and Plasmodium malariae Infections. Antimicrob Agents Chemother 41: 2317–2319.

Schwartz IK, Lackritz EM, Patchen LC (1991) Chloroquine-resistant Plasmodium vivax from Indonesia. N Engl J Med 324: 927.

Shao BR, Zhan CQ, Chen KY, Ye XY, Lin BY, Ha SH, and Zhang JX (1990) Experimental studies on combinations of pyronaridine/primaquine versus chloroquine/primaquine. Chin Med J 103:1024-1026.

Shao BR, Zhan CQ, Ye XY, Lin BY, Shang YZ, Ha SH, and Zhang JX (1988) Toxicity and tissue schizontocidal activity of primaquine combined with pyronaridine in laboratory animals. Chinese Journal of Parasitology and Parasitic Diseases 6: 22-25.

Silamchamroon U, Krudsood S, Treeprasertsuk S, Wilairatana P, Chalearmrult K, Mint HY, Maneekan P, White NJ, Gourdeur VR, Brittenham GM and Looareesuwan S (2003) Clinical trial of oral artesunate with or without high-dose primaquine for the treatment of vivax malaria in Thailand. Am J Trop Med Hyg 69: 14-18.

Sleigh AC, Liu XL, Jackson S, Li P, and Shang LY (1998) Resurgence of vivax malaria in Henan Province, China. Bull World Health Organ 76: 265-270.

Smithuis F, Kyaw MK, Phe O et al. (2006) Efficacy and effectiveness of dihydroartemisinin-piperaquine versus artesunate-mefloquine in falciparum malaria: an open-label randomised comparison. Lancet 367:207585).

Soto J, Toledo J, Gutierrez P, Luzz M, Llinas N, Cedeno N, Dunne M, and Berman J (2001) Plasmodium vivax clinically resistant to chloroquine in Colombia. Am J Trop Med Hyg 65: 90-93.

Sumawinata IW, Bernadeta, Leksana B, Sutamihardja A, Purnomo, Subianto B, Sekartuti, Fryauff DJ, Baird JK (2003) Very high risk of therapeutic failure with chloroquine for uncomplicated Plasmodium falciparum and P vivax malaria in Indonesian Papua. Am J Trop Med Hyg 69: 416-420.

Tjitra E, Baker J, Suprianto S, Cheng Q and Anstey NM (2002) Therapeutic efficacies of artesunate-sulfadoxine-pyrimethamine and chloroquine-sulfadoxine-pyrimethamine in vivax malaria pilot studies : relationship to Plasmodium vivax dhfr mutations. Antimicrob Agents Chemother 46 : 3947-3953.

Villalobos-Salcedo JM, Tada MS, Kimura E, Menezes MJ and Pereira-Da-Silva LH (2000) In-vivo sensitivity of Plasmodium vivax isolates from Rondônia (western Amazon region, Brazil) to regimens including chloroquine and primaquine. Ann Trop Med and Parasitol 94: 749-758.

Whitby M (1997) Drug resistant Plasmodium vivax malaria. Journal of Antimicrobial Chemotherapy 40: 749-752.

WHO Antimalarial drug combination therapy: Report of a WHO technical consultation. Geneva: World Health Organization; 2001.

Wilairatana P, Silachamroon U, Krudsood S, Singhasivanon P, Treeprasertsuk S, Bussaratid V, Phumratanaprapin W, Srivilirit S and Looareesuwan S (1999) Efficacy of primaquine regimens for primaquine-resistant Plasmodium vivax malaria in Thailand. Am J Trop Med Hyg 61: 973-977.

Woodrow CJ, Haynes RK and Krishna S (2005) Artemisinins. Postgrad Med J 81: 71-78.

Attachment 1

Protocol Approval and Signature Page

Approvals:

I have read and approve protocol SP-C-006-06 (Dated 28 August 2006). I confirm that this study will be conducted according to all stipulations of the protocol, including all statements regarding confidentiality.

This protocol contains information that is confidential and proprietary to MMV/Shin Poong and is to be used only for the purpose of the conduct of this study. Therefore, I must not disclose the contents of this protocol to any people other than the study personnel under my supervision, the Ethics Committees, or duly authorised representatives of regulatory agencies under the condition that they agree to keep this information confidential.

I may not use the information in this protocol for any other clinical study, or provide the information to any person or entity not listed above or publish it without the prior written permission of MMV/Shin Poong. If required to disclose any of this information to comply with regulatory regulations or requirements, I have to notify MMV/Shin Poong immediately.

I have read this protocol. I will conduct this study exactly as described in this protocol and agree that all details that I require to do so are provided in the protocol. I will provide copies of the protocol and access to all information furnished by MMV/Shin Poong to the personnel under my supervision involved in the conduct of this study. I will present and discuss the protocol and all other necessary information with them and ensure that they are completely informed about the contents of the protocol, all requirements for conducting the study as per protocol and know all relevant information about the drugs to be administered and all procedures to be conducted during this study.

The study may be terminated or enrolment suspended at any time by MMV/Shin Poong, with or without cause. I will terminate the study or suspend enrolment if this is, in my judgement, in the interests of the study patients and immediately notify MMV/Shin Poong.

I will conduct this study in accordance with all applicable regulations and Good Clinical Practice (GCP).

Principal Investigator	Place	Date and time

Site Details

Attachment 2 Study Plan

		Treatment period			Follow-up						
	Day 0	Day 0	Day 1	Day 2	Day 3	Day 7	Day 14	Day 21	Day 28 or day of failure	Day 35	Day 42
	Screening	Randomization									
Informed consent	•										
Study drug administration		•	•	•							
Primaquine administration[a]									•	•	•
Demographic Data/Medical History/History of medications & procedures	•										
Physical examination	•				•				•		•
Body weight	•								•		
Height	•										
Vital signs	•		•	•	•	•	•	•	•	•	•
Body temperature[b]	•	•	•	•	•	•	•	•	•	•	•
Clinical signs and symptoms of malaria	•		•	•	•	•[c]	•[c]	•[c]	•[c]	•[c]	•[c]
ECG (12-lead)	•			•		•[d]	•[d]		•		•[d]
Haematology	•				•	•			•		•
Blood Biochemistry /Urinalysis	•				•	•			•		•[e]
Thick blood films	•	•[f]	•[f]	•[f]	•	•	•	•	•	•	•
Thin blood films	•					•[g]	•[g]	•[g]	•[g]	•[g]	•[g]
Pyronaridine and artesunate concentration			•[h]	•[h]	•[h]	•[h]	•[h]	•[h]	•[h]	•[h]	•[h]
Molecular markers (PCR)		•	•			•[i]	•[i]	•[i]	•[i]	•[i]	•[i]
G-6-PD testing	•										
Urinary test for anti-malarial drugs[j]	•										
Urine hCG pregnancy test[k]	•						•		•		•
AE monitoring	Record throughout										
Concomitant medications/Procedures	Record throughout										

a) For patients with normal G-6-PD activity, oral primaquine treatment (once daily for 14 days) will be started on Day 28, after all assessments have been performed (including confirmation of negative pregnancy test for women of childbearing potential).
b) Every 8 hours over at least 72 hours following the first study drug administration or temperature normalisation for at least two readings 8 and 24 hours apart, then at each visit.
c) If clinically indicated and any day in case of fever.
d) If clinically indicated.
e) Should be performed only if clinically significant abnormalities are observed.
f) Thick blood films will be examined every 8 hours until at least 72 hours or until a negative smear had been recorded for at least between 8 hours and 24 hours apart, then at each visit.
g) Thin blood smear will also be taken from Day 7 whenever parasitological blood samples are taken, and reserved in the event of recrudescence, to confirm *Plasmodium* species.
h) A blood sample to measure pyronaridine levels must be taken in case of treatment failure. Furthermore, for pyronaridine population PK, 1 sample will be taken between D0 (post-dose) and D3 and another between D4 and D42. For artesunate PK, 1 sample will be taken on D0 (post-dose) or on D2 and a second one on D3.
i) PCR samples will be collected and reserved. Centralized PCR analysis will be performed at baseline and in case of re-appearing parasitaemia.
j) If feasible
k) For females of childbearing potential.

Attachment 3

Classification of Treatment Outcomes

From: Monitoring Antimalarial Drug Resistance

Report of a WHO Consultation

Geneva, Switzerland, 3-5 December 2001

The definition of treatment failure includes the following:

- Clinical deterioration due to *P. vivax* illness requiring hospitalization in presence of parasitemia.
- Presence of parasitemia and axillary temperature ≥ 37.5°C any time between Day 3 and Day 28.
- Presence of parasitemia on any day between Day 7 and Day 28, irrespective of clinical conditions.

Note: Subjects presenting a *P. falciparum* infection after clearance of *P. vivax* parasites should be treated accordingly and withdrawn. These should not be considered as *P. vivax* treatment failures.

Cure on Day 14

Cases with no parasitaemia on Day 14 irrespective of axillary temperature without previously meeting any of the criteria of treatment failure mentioned above throughout the follow-up period.

Cure on Day 28

Cases with no parasitaemia on Day 28 irrespective of axillary temperature without previously meeting any of the criteria of treatment failure mentioned above throughout the follow-up period.

Attachment 4

Dosing regimens

Pyronaridine artesunate 180:60 mg or matching placebo, once daily

Weight (kg)	Number of tablets Day 0,1,2
20-25	1
26-44	2
45-64	3
65-90	4

Chloroquine base 155 mg or matching placebo, once daily

Adult Dosing Regimen

	Day 0	Day 1	Day 2
Number of tablets	4	4	2

Children Dosing Regimen

Patient's weight (kg)	Number of tablets Days 0 and 1	Number of tablets Day 2
20-23	1	0.5
24-27	1.5	1
28-38	2	1
39-40	3	1.5

Primaquine base 15 mg, once daily

Adult Dosing Regimen

	Days 28 through 42
Number of tablets	1

Children Dosing Regimen

Patient's weight (kg)	Number of tablets Days 28 through 42
20-35	0.5
>35	1

Attachment 5

Forbidden Medications

The following medication (with antimalarial activity) should not be used within the 14-day period prior to enrolment or during the entire study period:

4-Aminoquinolones : chloroquine (when not being used as study material).

Arylaminoalcohols quinidine

Quinine.

9-phenanthrene methanol halofandrine.

Dibutyl-amino ethanol lumefantrine

Artemisinin derivatives : artemether, DHA, artesunate.

Proguanil.

Chlorproguanil.

Sulfalene.

Pyrimethamine.

Sulfonamides sulfadoxine.

Sulfisoxazole.

Sulfadiazine.

Sulfasalazine.

8-Aminoquinolines: primaquine (when not being used as study material).

Hydroxynaphtolquinone atovaquone.

Antibiotics, various classes doxycycline.

Azythromycin.

Erythromycin.

Pentamidine.

Clindamycin.

Rifampin.

Rifabutin.

Fluoroquinolones : ciprofloxacin, pefloxacin, norfloxacin, ofloxacin.

Dapsone.

Combinations of sulfadoxine pyrimethamine.

Trimethoprim sulfamethoxazole.

Atovaquone proguanil.

Artemether lumefantrine.

Attachment 6

Drug Concentration Specimen Collection, Processing, Storage and Shipping

Specimen Collection, Processing and Storage

Blood Collection for Pyronaridine analysis

Collect 1 ml whole blood in sterile glass tubes containing sodium heparin as the anticoagulant (Vacutainer tubes). The sodium heparin tube should be at room temperature (18°-25° C) prior to use. **The sample may be stored on ice for up to 2 hours before processing.**Transfer the blood into two approximately equal volume aliquots in screw cap cryovials (Nalgene No.: 5000 0012). Freeze the whole blood samples at or below -80°C. Do not thaw sample after freezing.

Pyronaridine levels for drug failures: collect 1 ml whole blood in sterile glass tubes containing sodium heparin as the anticoagulant (Sodium heparin Vacutainer(r) tubes). The sodium heparin tube should be at room temperature prior to use. The sample may be stored on ice for up to 2 hours before processing. Transfer the blood into two approximately equal volume aliquots in screw cap cryovials (Nalgene No.: 5000 0012). Freeze the whole blood samples at or below -80°C in a laboratory freezer. Do not thaw sample after freezing. At site where no -80°C freezer is available, the sample can be frozen and stored up to 4 weeks in a -18°C freezer prior being collected or stored in a -80°C freezer at another location.

Plasma Collection for Artesunate Analysis

Collect 1 ml blood in tubes containing potassium oxalate/sodium fluoride (BD Vacutainer No.: 367925 or equivalent) for the separation of plasma drawn at times specified. The tubes should be pre-chilled on wet ice prior to use. After collection of blood, the tube should be placed on wet ice and processed as soon as possible after collection (the samples can be placed in the ice bath for 5 to 10 minutes prior to centrifugation to allow the blood to chill in the tube). Centrifuge blood within 15 minutes of collection. Centrifuge whole blood at 1000-1200 x g for 10 minutes. Immediately after centrifugation, remove plasma from cells and transfer the plasma into two approximately equal volume aliquots in screw cap cryovials

(Nalgene No.: 5000 0012). Immediately freeze the plasma samples at or below -80°C in a laboratory freezer. Do not thaw sample after freezing.

Specimen Packaging, Labelling and Shipping

Packaging

All samples must be transported frozen on dry ice. Specimens should be properly packaged to ensure safe arrival at the analytical laboratory. Combine sample tubes for each subject in one container. The two aliquots should be shipped in separate shipments, with one complete set of secondary samples held frozen in reserve, until notification that the primary set of samples has been received by the analytical laboratory and final PK data have been obtained.

Labelling

Each cryovial must be labelled with protocol number, subject identification, draw date, and draw time.

Shipping

Samples should be shipped via air express frozen on dry ice to:
Professor Lawrence Fleckenstein
College of Pharmacy, S-427 PHAR
115 South Grand Avenue
University of Iowa
Iowa City, IA 52242
United States of America
Phone: + 1 319-335-8804
Mobile: + 1 319-321-2901
Fax: + 1 319-353-5646

The samples should be sent with a sample inventory included in the shipping container.

The samples should be sent early in the week (Monday or Tuesday) to allow receipt on Monday through Saturday only.

Please email l-fleckenstein@uiowa.edu to advise that a shipment has been sent and to provide an air bill number. Shipments will be tracked by the analytical laboratory.

Please email l-fleckenstein@uiowa.edu around holiday times to check available days for shipping.

Attachment 7

DIVISION OF MICROBIOLOGY AND INFECTIOUS DISEASES (DMID) Toxicity Grading Scales for Determining Severity of Adverse Events

ADULT TOXICITY TABLES

ABBREVIATIONS: Abbreviations utilised in the Table:

ULN = Upper Limit of Normal LLN = Lower Limit of Normal
R_x = Therapy Req = Required
Mod = Moderate IV = Intravenous
ADL = Activities of Daily Living Dec = Decreased

ESTIMATING SEVERITY GRADE

For abnormalities NOT found elsewhere in the Toxicity Tables use the scale below to estimate grade of severity:

GRADE 1 Mild Transient or mild discomfort
(< 48 hours); no medical intervention/therapy required

GRADE 2 Moderate Mild to moderate limitation in activity - some assistance may be needed; no or minimal medical intervention/therapy required

GRADE 3 Severe Marked limitation in activity, some assistance usually required; medical intervention/therapy required, hospitalizations possible

GRADE 4 Life-threatening Extreme limitation in activity, significant assistance required; significant medical intervention/therapy required, hospitalization or hospice care probable

SERIOUS OR LIFE-THREATENING AEs

ANY clinical event deemed by the clinician to be serious or life-threatening should be considered a grade 4 event. Clinical events considered to be serious or life-threatening include, but are not limited to: seizures, coma, tetany, diabetic ketoacidosis, disseminated intravascular coagulation, diffuse petechiae, paralysis, acute psychosis, severe depression.

**HEMATOLOGY**				
	Grade 1	Grade 2	Grade 3	Grade 4
Haemoglobin	9.5 - 10.5 gm/dL	8.0 - 9.4gm/dL	6.5 - 7.9 gm/dL	< 6.5 gm/dL
Absolute Neutrophil Count	1000-1500/mm^3	750-999/mm^3	500-749/mm^3	<500/mm^3
Platelets	75,000-99,999/mm^3	50,000-74,999/mm^3	20,000-49,999/mm^3	<20,000/mm^3
WBCs	> 13,000/ mm^3	13,000-15,000 /mm^3	15,000-30,000/mm^3	>30,000 or <1,000 /mm^3
% Polymorphonuclear Leucocytes + Band Cells	> 80%	90 – 95%	>95%	----------
Abnormal Fibrinogen	Low: 100-200 mg/dL High: 400-600 mg/dL	Low: <100 mg/dL High: >600 mg/dL	Low: < 50 mg/dL ----------	Fibrinogen associated with gross bleeding or with disseminated coagulation
Fibrin Split Product	20-40 mcg/ml	41-50 mcg/ml	51-60 mcg/ml	> 60 mcg/ml
Prothrombin Time (PT)	1.01 - 1.25 x ULN	1.26-1.5 x ULN	1.51 -3.0 x ULN	>3 x ULN
Activated Partial Thromboplastin (APPT)	1.01 -1.66 x ULN	1.67 - 2.33 x ULN	2.34 - 3 x ULN	> 3 x ULN
Methaemoglobin	5.0 - 9.9 %	10.0 - 14.9 %	15.0 - 19.9%	> 20.0 %

**CHEMISTRIES**				
	Grade 1	Grade 2	Grade 3	Grade 4
Hyponatraemia	130-135 mEq/L	123-129 mEq/L	116-122 mEq/L	< 116 mEq/L or abnormal sodium with mental status changes or seizures
Hypernatraemia	146-150 mEq/L	151-157 mEq/L	158-165 mEq/L	> 165 mEq/L or abnormal sodium with mental status changes or seizures
Hypokalaemia	3.0 - 3.4 mEq/L	2.5 - 2.9 mEq/L	2.0 - 2.4 mEq/L or intensive replacement therapy or hospitalization required	< 2.0 mEq/L or abnormal potassium with paresis, ileus or life-threatening arrhythmia
Hyperkalaemia	5.6 - 6.0 mEq/L	6.1 - 6.5 mEq/L	6.6 - 7.0 mEq/l	> 7.0 mEq/L or abnormal potassium with life-threatening arrhythmia

**CHEMISTRIES (Continued)**				
	Grade 1	Grade 2	Grade 3	Grade 4
Hypoglycaemia	55-64 mg/dL	40-54 mg/dL	30-39 mg/dL	<30 mg/dL or abnormal glucose with mental status changes or coma
Hyperglycaemia (nonfasting and no prior diabetes)	116 - 160 mg/dL	161- 250 mg/dL	251 - 500 mg/dL	> 500 mg/dL or abnormal glucose with ketoacidosis or seizures
Hypocalcaemia (corrected for albumin)	8.4 - 7.8 mg/dL	7.7 - 7.0 mg/dL	6.9 - 6.1 mg/dL	< 6.1 mg/dL or abnormal calcium with life threatening arrhythmia or tetany
Hypercalcaemia (correct for albumin)	10.6 - 11.5 mg/dL	11.6 - 12.5 mg/dL	12.6 - 13.5 mg/dL	> 13.5 mg/dL or abnormal calcium with life threatening arrhythmia
Hypomagnesaemia	1.4 - 1.2 mEq/L	1.1 - 0.9 mEq/L	0.8 - 0.6 mEq/L	< 0.6 mEq/L or abnormal magnesium with life-threatening arrhythmia
Hypophosphataemia	2.0 - 2.4 mg/dL	1.5 -1.9 mg/dL or replacement Rx required	1.0 -1.4 mg/dL intensive therapy or hospitalization required	< 1.0 mg/dL or abnormal phosphate with life-threatening arrhythmia
Hyperbilirubinaemia	1.1 - 1.5 x ULN	1.6 - 2.5 x ULN	2.6 - 5 x ULN	> 5 x ULN
BUN	1.25 - 2.5 x ULN	2.6 - 5 x ULN	5.1 - 10 x ULN	> 10 x ULN
Hyperuricaemia (uric acid)	7.5 – 10.0 mg/dL	10.1 – 12.0 mg/dL	12.1 – 15.0 mg/dL	>15.0 mg/dL
Creatinine	1.1 - 1.5 x ULN	1.6 - 3.0 x ULN	3.1 - 6 x ULN	> 6 x ULN or dialysis required

**ENZYMES**				
	Grade 1	Grade 2	Grade 3	Grade 4
AST (SGOT)	1.25 - 2.5 x ULN	2.6 - 5 x ULN	5.1 - 10 x ULN	> 10 x ULN
ALT (SGPT)	1.25 - 2.5 x ULN	2.6 - 5 x ULN	5.1 - 10 x ULN	> 10 x ULN
GGT	1.25 -2.5 x ULN	2.6 - 5 x ULN	5.1 - 10 x ULN	> 10 x ULN
Alkaline Phosphatase	1.25 - 2. 5 x ULN	1.6 - 5 x ULN	5.1 - 10 x ULN	> 10 x ULN
Amylase	1.1 - 1.5 x ULN	1.6 - 2.0 x ULN	2.1 - 5.0 x ULN	> 5.1 x ULN
Lipase	1.1 - 1.5 x ULN	1.6 - 2.0 x ULN	2.1 - 5.0 x ULN	> 5.1 x ULN

**URINALYSIS**				
	Grade 1	Grade 2	Grade 3	Grade 4
Proteinuria	1+<br>or<br>200 mg - 1 gm loss/day	2-3+<br>or<br>1- 2 gm loss/day	4+<br>or<br>2-3.5 gm loss/day	nephrotic syndrome<br>or<br>> 3.5 gm loss/day
Haematuria	microscopic only<br><10 rbc/hpf	gross, no clots<br>>10 rbc/hpf	gross, with or without clots, OR red blood cell casts	obstructive or required transfusion

**CARDIOVASCULAR**				
	Grade 1	Grade 2	Grade 3	Grade 4
Cardiac Rhythm		asymptomatic, transient signs, no Rx required	recurrent/persistent; symptomatic Rx required	unstable dysrhythmia; hospitalization and treatment required
Hypertension	transient increase<br>> 20 mm/Hg; no treatment	recurrent, chronic increase<br>> 20mm/Hg.<br>/treatment required	acute treatment required; outpatient treatment or hospitalization possible	end organ damage or hospitalization required
Hypotension	transient orthostatic hypotension with heart rate increased by <20 beat/min or decreased by <10 mm Hg systolic BP, No treatment required	symptoms due to orthostatic hypotension or BP decreased by <20 mm Hg systolic; correctable with oral fluid treatment	requires IV fluids; no hospitalization required	mean arterial pressure<br><60mm/ Hg or end organ damage or shock; requires hospitalization and vasopressor treatment
Pericarditis	minimal effusion	mild/moderate asymptomatic effusion, no treatment	symptomatic effusion; pain; EKG changes	tamponade; pericardiocentesis or surgery required
Haemorrhage, Blood Loss	microscopic/occult	mild, no transfusion	gross blood loss;<br>1-2 units transfused	massive blood loss; > 3 units transfused

RESPIRATORY				
	Grade 1	Grade 2	Grade 3	Grade 4
Cough	transient- no treatment	persistent cough; treatment responsive	Paroxysmal cough; uncontrolled with treatment	--------------------
Bronchospasm, Acute	transient; no treatment; 70% - 80% FEV_1 of peak flow	requires treatment; normalizes with bronchodilator; FEV_1 50% - 70% (of peak flow)	no normalization with bronchodilator; FEV_1 25% - 50% of peak flow; or retractions present	cyanosis: FEV_1 < 25% of peak flow or intubation necessary
Dyspnoea	dyspnoea on exertion	dyspnoea with normal activity	dyspnoea at rest	dyspnoea requiring Oxygen therapy

GASTROINTESTINAL				
	Grade 1	Grade 2	Grade 3	Grade 4
Nausea	mild or transient; maintains reasonable intake	moderate discomfort; intake decreased significantly; some activity limited	no significant intake; requires IV fluids	hospitalization required;
Vomiting	1 episode in 24 hours	2-5 episodes in 24 hours	>6 episodes in 24 hours or needing IV fluids	physiologic consequences requiring hospitalization or requiring parenteral nutrition
Constipation	requiring stool softener or dietary modification	requiring laxatives	obstipation requiring manual evacuation or enema	obstruction or toxic megacolon
Diarrhoea	mild or transient; 3-4 loose stools/day or mild diarrhoea last < 1 week	moderate or persistent; 5-7 loose stools/day or diarrhoea lasting >1 week	>7 loose stools/day or bloody diarrhoea; or orthostatic hypotension or electrolyte imbalance or >2L IV fluids required	hypotensive shock or physiologic consequences requiring hospitalization
Oral Discomfort/ Dysphagia	mild discomfort; no difficulty swallowing	some limits on eating/drinking	eating/talking very limited; unable to swallow solid foods	unable to drink fluids; requires IV fluids

NEUROLOGICAL				
	Grade 1	Grade 2	Grade 3	Grade 4
Neuro-Cerebellar	slight incoordination dysdiadochokinesis	intention tremor, dysmetria, slurred speech; nystagmus	locomotor ataxia	incapacitated
Psychiatric	mild anxiety or depression	moderate anxiety or depression; therapy required; change in normal routine	severe mood changes requiring therapy; or suicidal ideation; or aggressive ideation	acute psychosis requiring hospitalization; or suicidal gesture/attempt or hallucinations
Muscle Strength	subjective weakness no objective symptoms/ signs	mild objective signs/symptoms no decrease in function	objective weakness function limited	paralysis
Paresthesia (burning, tingling, etc.)	mild discomfort; no treatment required	moderate discomfort; non-narcotic analgesia required	severe discomfort; or narcotic analgesia required with symptomatic improvement	incapacitating; or not responsive to narcotic analgesia
Neuro-sensory	mild impairment in sensation (decreased sensation, e.g., vibratory, pinprick, hot/cold in great toes) in focal area or symmetrical distribution; or change in taste, smell, vision and/or hearing	moderate impairment (mod decreased sensation, e.g., vibratory, pinprick, hot/cold to ankles) and/or joint position or mild impairment that is not symmetrical	severe impairment (decreased or loss of sensation to knees or wrists) or loss of sensation of at least mod degree in multiple different body areas (i.e., upper and lower extremities)	sensory loss involves limbs and trunk; paralysis; or seizures

**MUSCULOSKELETAL**				
	Grade 1	Grade 2	Grade 3	Grade 4
Arthralgia (joint pain)	mild pain not interfering with function	moderate pain, analgesics and/or pain interfering with function but not with activities of daily living	severe pain; pain and/or analgesics interfering with activities of daily living	disabling pain
Arthritis	mild pain with inflammation, erythaema or joint swelling – but not interfering with function	moderate pain with inflammation, erythaema or joint swelling – interfering with function, but not with activities of daily living	severe pain with inflammation, erythaema or joint swelling –and interfering with activities of daily living	permanent and/or disabling joint destruction
Myalgia	myalgia with no limitation of activity	muscle tenderness (at other than injection site) or with moderate impairment of activity	severe muscle tenderness with marked impairment of activity	frank myonecrosis

**SKIN**				
	Grade 1	Grade 2	Grade 3	Grade 4
Mucocutaneous	erythaema; pruritus	diffuse, maculo papular rash, dry desquamation	vesiculation or moist desquamation or ulceration	exfoliative dermatitis, mucous membrane involvement or erythaema, multiforme or suspected Stevens-Johnson or necrosis requiring surgery
Induration	< 15mm	15-30 mm	>30mm	
Erythaema	< 15mm	15-30 mm	>30mm	
Oedema	< 15mm	15-30 mm	>30mm	
Rash at Injection Site	< 15mm	15-30 mm	>30mm	
Pruritus	slight itching at injection site	moderate itching at injection extremity	itching over entire body	

**SYSTEMIC**				
	Grade 1	Grade 2	Grade 3	Grade 4
Allergic Reaction	pruritus without rash	localized urticaria	generalized urticaria; angioedema	anaphylaxis
Headache	mild, no treatment required	transient, moderate; treatment required	severe; responds to initial narcotic therapy	intractable; requires repeated narcotic therapy
Fever: oral	37.7 - 38.5 C or 100.0 - 101.5 F	38.6 - 39.5 C or 101.6 - 102.9 F	39.6 - 40.5 C or 103 - 105 F	> 40 C or > 105 F
Fatigue	normal activity reduced < 48 hours	normal activity decreased 25-50% > 48 hours	normal activity decreased > 50% can't work	unable to care for self

PEDIATRIC TOXICITY TABLES

**LOCAL REACTIONS**				
	**GRADE 1**	**GRADE 2**	**GRADE 3**	**GRADE 4**
Induration	< 10mm	10-25 mm	26-50mm	>50mm
Erythaema	< 10mm	10-25 mm	26-50mm	>50mm
Oedema	< 10mm	10-25 mm	26-50mm	>50mm
Rash at Injection Site	< 10mm	10-25 mm	26-50mm	>50mm
Pruritus	Slight itching at injection site	Moderate itching at injection extremity	Itching at injection extremity and other sites	Itching over entire body

**HEMATOLOGY**				
	**GRADE 1**	**GRADE 2**	**GRADE 3**	**GRADE 4**
Haemoglobin for children greater that 2 years of age	10-10.9 gm/dL	7.0-9.9 gm/dL	<7.0 gm/dL	Cardiac Failure secondary to anaemia
Absolute Neutrophil Count	750-1200/mm^3	400-749/mm^3	250-399/mm^3	<250/mm^3
Platelets	-------	50,000-75,000/mm^3	25,000-49,999/mm^3	<25,000/mm^3
Prothrombin Time (PT)	1.1-1.2 x ULN	1.3 -1.5 x ULN	1.6 -3.0 x ULN	>3.0 x ULN
Partial Thromboplastin Time (PTT)	1.1-1.6 x ULN	1.7-2.3 x ULN	2.4 -3.0 x ULN	>3.0 x ULN

**GASTROINTESTINAL**				
	**GRADE 1**	**GRADE 2**	**GRADE 3**	**GRADE 4**
Bilirubin	1.1-1.9 x ULN	2.0-2.9 x ULN	3.0-7.5 x ULN	>7.5 x ULN
AST (SGOT)	1.1-4.9 x ULN	5.0-9.9 x ULN	10.0-15.0 x ULN	>15.0 x ULN
ALT (SGPT)	1.1-4.9 x ULN	5.0-9.9 x ULN	10.0-15.0 x ULN	>15.0 x ULN
GGT	1.1-4.9 x ULN	5.0-9.9 x ULN	10.0-15.0 x ULN	>15.0 x ULN
Pancreatic Amylase	1.1-1.4 x ULN	1.5-1.9 x ULN	2.0-3.0 x ULN	>3.0 x ULN
Uric Acid	7.5-9.9mg/dL	10-12.4 mg/dL	12.5-15.0 mg/dL	>15.0 mg/dL
CPK	See Neuromuscular Toxicity			
Appetite	-----------------	Decreased appetite	Appetite very decreased, no solid food taken	No solid or liquid taken
Abdominal Pain	Mild	Moderate- No Treatment Needed	Moderate- Treatment Needed	Severe- Hospitalized for treatment
Diarrhoea	Slight change in consistency and/or frequency of stools	Liquid stools	Liquid stools greater that 4x the amount or number normal for this child	Liquid stools greater than 8x the amount or number normal for this child
Constipation	Slight change in the consistency/frequency of stool	Hard, dry stools with a change in frequency	Abdominal pain	Distension and Vomiting
Nausea	Mild	Moderate- Decreased oral intake	Severe-Little oral intake	Unable to ingest food or fluid for more than 24 hours
Vomiting	1 episode/day	2-3 episodes per day	4-6 episodes per day	Greater than 6 episodes per day or Intractable Vomiting

**ELECTROLYTES**				
	**GRADE 1**	**GRADE 2**	**GRADE 3**	**GRADE 4**
CREATININE				
2 Years- 12 Years of age	0.7-1.0 mg/dL	1.1-1.6 mg/dL	1.7-2.0 mg/dL	>2.0 mg/dL
Greater than 12 Years of age	1.0-1.7 mg/dL	1.8-2.4 mg/dL	2.5-3.5 mg/dL	>3.5 mg/dL
Hypernatraemia		<145-149 mEq/L	150-155 mEq/L	>155 mEq/L or abnormal sodium AND mental status changes
Hyponatraemia		130-135 mEq/L	129-124 mEq/L	<124 mEq/L or abnormal sodium AND mental status changes
Hyperkalaemia	5.0-5.9 mEq/L	6.0-6.4 mEq/L	6.5-7.0 mEq/L	>7.0 mEq/L or abnormal potassium AND cardiac arrhythmia
Hypokalaemia	3.0-3-5 mEq/L	2.5-2.9 mEq/L	2.0-2.4 mEq/L	<2.0 mEq/L or abnormal potassium AND cardiac arrhythmia
Hypercalcaemia	10.5-11.2mg/dL	11.3-11.9 mg/dL	12.0-12.9 mg/dL	>13.0 mg/dL
Hypocalcaemia	7.8-8.4 mg/dL	7.0-7.7 mg/dL	6.0-6.9 mg/dL	<6.0 mg/dL
Hypomagnesaemia	1.2-1.4 mEq/L	0.9-1.1 mEq/L	0.6-0.8 mEq/L	<0.6 mEq/L or abnormal magnesium AND cardiac arrhythmia
Hypoglycaemia	55-65 mg/dL	40-54 mg/dL	30-39 mg/dL	<30 mg/dL or abnormal glucose AND mental status changes
Hyperglycaemia	116-159 mg/dL	160-249 mg/dL	250-400 mg/dL	>400 mg/dL or ketoacidosis
Proteinuria	Tr-1+ or <150 mg/day	2+ or 150-499 mg/day	3+ or 500-1000 mg/day	4+ or Nephrotic syndrome >1000 mg/day
Haematuria	Microscopic <25 cells/hpf	Microscopic >25 cells/hpf		Gross haematuria

**CENTRAL NERVOUS SYSTEM (CNS)**				
	**GRADE 1**	**GRADE 2**	**GRADE 3**	**GRADE 4**
Generalized CNS Symptoms			Dizziness	Hypotonic, hyporesponsive episodes;<br>Seizures;<br>Apnoea/Bradycardia;<br>Inconsolable crying > 3 hrs;
Headache	Mild	Moderate, Responds to non-narcotic analgesia	Moderate to Severe, Responds to narcotic analgesia	Intractable
Level of Activity		Slightly irritable OR slightly subdued	Very irritable OR Lethargic	Inconsolable OR Obtunded
Visual		Blurriness, diplopia, or horizontal nystagmus of < 1 hour duration, with spontaneous resolution	More than 1 episode of Grade 2 symptoms per week, or an episode of Grade 2 symptoms lasting more than 1 hour with spontaneous resolution by 4 hours or vertical nystagmus	Decrease in visual acuity, visual field deficit, or oculogyric crisis
Myelopathy		None	None	Myelopathic/spinal cord symptoms, such as: pyramidal tract weakness and disinhibition, sensory level, loss of proprioception, bladder/bowel dysfunction

**PERIPHERAL NERVOUS SYSTEM**				
	**GRADE 1**	**GRADE 2**	**GRADE 3**	**GRADE 4**
Neuropathy/<br>Lower Motor Neuropathy		Mild transient Parasthaesia only	Persistent or progressive parasthaesias, burning sensation in feet, or mild dysesthesia; no weakness; mild to moderate deep tendon reflex changes; no sensory loss	Onset of significant weakness, decrease or loss of DTRs, sensory loss in "stocking glove" distribution, radicular sensory loss, multiple cranial nerve involvement; bladder or bowel dysfunction, fasciculations, respiratory embarrassment from chest wall weakness.
Myopathy or Neuromuscular Junction Impairment	Normal or mild (<2 x ULN) CPK elevation	Mild proximal weakness and/or atrophy not affecting gross motor function. Mild myalgias, +/- mild CPK elevation (<2 x ULN)	Proximal muscle weakness and/or atrophy affecting motor function +/- CPK elevation; or severe myalgias with CPK >2 x ULN;	Onset of myasthenia-like symptoms (fatigable weakness with external, variable ophthalmoplegia and/or ptosis), or neuromuscular junction blockade (acute paralysis) symptoms

OTHER				
	**GRADE 1**	**GRADE 2**	**GRADE 3**	**GRADE 4**
Allergy	Pruritus without Rash	Pruritic Rash	Mild Urticaria	Severe Urticaria Anaphylaxis, Angioedema
Drug Fever (Rectal)	.	38.5-40C 101.3 – 104.0F	Greater than 40.0C Greater than 104.0F	Sustained Fever: Equal or greater than 40C (104.0F) for longer than 5 days
Cutaneous	Localized rash	Diffuse maculopapular Rash	Generalized urticaria	Stevens-Johnson Syndrome or Erythaema multiforme
Stomatitis	Mild discomfort	Painful, difficulty swallowing, but able to eat and drink	Painful: unable to swallow solids	Painful: unable to swallow liquids; requires IV fluids
Clinical symptoms not otherwise specified in this table	No therapy; monitor condition	May require minimal intervention and monitoring	Requires medical care and possible hospitalization	Requires active medical intervention, hospitalization, or hospice care
Laboratory values not otherwise specified in this table	Abnormal, but requiring no immediate intervention; follow	Sufficiently abnormal to require evaluation as to causality and perhaps mild therapeutic intervention, but not of sufficient severity to warrant immediate changes in study drug	Sufficiently severe to require evaluation and treatment, including at least temporary suspension of study drug	Life-threatening severity; Requires immediate evaluation, treatment, and usually hospitalization; Study drug must be stopped immediately and should not be restarted until the abnormality is clearly felt to be caused by some other mechanism that study drug

Attachment 8

DECLARATION OF HELSINKI

Ethical Principles for Medical Research Involving Human Subjects

Adopted by the 18th WMA General Assembly
Helsinki, Finland, June 1964
and amended by the
29th WMA General Assembly, Tokyo, Japan, October 1975
35th WMA General Assembly, Venice, Italy, October 1983
41st WMA General Assembly, Hong Kong, September 1989
48th WMA General Assembly, Somerset West, Republic of South Africa, October 1996
and the
52nd WMA General Assembly, Edinburgh, Scotland, October 2000

Note of Clarification on Paragraph 29 added by the WMA General Assembly, Washington 2002
Note of Clarification on Paragraph 30 added by the WMA General Assembly, Tokyo 2004

A. INTRODUCTION

1. The World Medical Association has developed the Declaration of Helsinki as a statement of ethical principles to provide guidance to physicians and other participants in medical research involving human subjects. Medical research involving human subjects includes research on identifiable human material or identifiable data.

2. It is the duty of the physician to promote and safeguard the health of the people. The physician's knowledge and conscience are dedicated to the fulfilment of this duty.

3. The Declaration of Geneva of the World Medical Association binds the physician with the words, "The health of my patient will be my first consideration," and the International Code of Medical Ethics declares that, "A physician shall act only in the patient's interest when providing medical care which might have the effect of weakening the physical and mental condition of the patient."

4. Medical progress is based on research which ultimately must rest in part on experimentation involving human subjects.

5. In medical research on human subjects, considerations related to the well-being of the human subject should take precedence over the interests of science and society.

6. The primary purpose of medical research involving human subjects is to improve prophylactic, diagnostic and therapeutic procedures and the understanding of the aetiology and pathogenesis of disease. Even the best proven prophylactic, diagnostic, and therapeutic methods must continuously be challenged through research for their effectiveness, efficiency, accessibility and quality.

7. In current medical practice and in medical research, most prophylactic, diagnostic and therapeutic procedures involve risks and burdens.

8. Medical research is subject to ethical standards that promote respect for all human beings and protect their health and rights. Some research populations are vulnerable and need special protection. The particular needs of the economically and medically disadvantaged must be recognised. Special attention is also required for those who cannot give or refuse consent for themselves, for those who may be subject to giving consent under duress, for those who will not benefit personally from the research and for those for whom the research is combined with care.

9. Research Investigators should be aware of the ethical, legal and regulatory requirements for research on human subjects in their own countries as well as applicable international requirements. No national ethical, legal or regulatory requirement should be allowed to reduce or eliminate any of the protections for human subjects set forth in this Declaration.

B. BASIC PRINCIPLES FOR ALL MEDICAL RESEARCH

10. It is the duty of the physician in medical research to protect the life, health, privacy, and dignity of the human subject.

11. Medical research involving human subjects must conform to generally accepted scientific principles, be based on a thorough knowledge of the scientific literature, other relevant sources of information, and on adequate laboratory and, where appropriate, animal experimentation.

12. Appropriate caution must be exercised in the conduct of research which may affect the environment, and the welfare of animals used for research must be respected.

13. The design and performance of each experimental procedure involving human subjects should be clearly formulated in an experimental protocol. This protocol should be submitted for consideration, comment, guidance, and where appropriate, approval to a specially appointed ethical review committee, which must be independent of the investigator, the sponsor or any other kind of undue influence. This independent committee should be in conformity with the laws and regulations of the country in which the research experiment is performed. The committee has the right to monitor ongoing trials. The researcher has the obligation to provide monitoring information to the committee, especially any serious adverse events. The researcher should also submit to the committee, for review, information regarding funding, sponsors, institutional affiliations, other potential conflicts of interest and incentives for subjects.

14. The research protocol should always contain a statement of the ethical considerations involved and should indicate that there is compliance with the principles enunciated in this Declaration.

15. Medical research involving human subjects should be conducted only by scientifically qualified persons and under the supervision of a clinically competent medical person. The responsibility for the human subject must always rest with a medically qualified person and never rest on the subject of the research, even though the subject has given consent.

16. Every medical research project involving human subjects should be preceded by careful assessment of predictable risks and burdens in comparison with foreseeable benefits to the subject or to others. This does not preclude the participation of healthy volunteers in medical research. The design of all studies should be publicly available.

17. Physicians should abstain from engaging in research projects involving human subjects unless they are confident that the risks involved have been adequately assessed and can be satisfactorily managed. Physicians should cease any investigation if the risks are found to outweigh the potential benefits or if there is conclusive proof of positive and beneficial results.

18. Medical research involving human subjects should only be conducted if the importance of the objective outweighs the inherent risks and burdens to the subject. This is especially important when the human subjects are healthy volunteers.

19. Medical research is only justified if there is a reasonable likelihood that the populations in which the research is carried out stand to benefit from the results of the research.

20. The subjects must be volunteers and informed participants in the research project.

21. The right of research subjects to safeguard their integrity must always be respected. Every precaution should be taken to respect the privacy of the subject, the confidentiality of the patient's information and to minimise the impact of the study on the subject's physical and mental integrity and on the personality of the subject.

22. In any research on human beings, each potential subject must be adequately informed of the aims, methods, sources of funding, any possible conflicts of interest, institutional affiliations of the researcher, the anticipated benefits and potential risks of the study and the discomfort it may entail. The subject should be informed of the right to abstain from participation in the study or to withdraw consent to participate at any time without reprisal. After ensuring that the subject has understood the information, the physician should then obtain the subject's freely-given informed consent, preferably in writing. If the consent cannot be obtained in writing, the non-written consent must be formally documented and witnessed.

23. When obtaining informed consent for the research project the physician should be particularly cautious if the subject is in a dependent relationship with the physician or may consent under duress. In that case the informed consent should be obtained by a well-informed physician who is not engaged in the investigation and who is completely independent of this relationship.

24. For a research subject who is legally incompetent, physically or mentally incapable of giving consent or is a legally incompetent minor, the investigator must obtain informed consent from the legally authorised representative in accordance with applicable law. These groups should not be included in research unless the research is necessary to promote the health of the population represented and this research cannot instead be performed on legally competent persons.

25. When a subject deemed legally incompetent, such as a minor child, is able to give assent to decisions about participation in research, the investigator must obtain that assent in addition to the consent of the legally authorised representative.

26. Research on individuals from whom it is not possible to obtain consent, including proxy or advance consent, should be done only if the physical/mental condition that prevents obtaining informed consent is a necessary characteristic of the research population. The specific reasons for involving research subjects with a condition that renders them unable to give informed consent should be stated in the experimental protocol for consideration and approval of the review committee. The protocol should state that consent to remain in the research should be obtained as soon as possible from the individual or a legally authorised surrogate.

27. Both authors and publishers have ethical obligations. In publication of the results of research, the investigators are obliged to preserve the accuracy of the results. Negative as well as positive results should be published or otherwise publicly available. Sources of funding, institutional affiliations and any possible conflicts of interest should be declared in the publication. Reports of experimentation not in accordance with the principles laid down in this Declaration should not be accepted for publication.

ADDITIONAL PRINCIPLES FOR MEDICAL RESEARCH COMBINED WITH MEDICAL CARE

28. The physician may combine medical research with medical care, only to the extent that the research is justified by its potential prophylactic, diagnostic or therapeutic value. When medical research is combined with medical care, additional standards apply to protect the patients who are research subjects.

29. The benefits, risks, burdens and effectiveness of a new method should be tested against those of the best current prophylactic, diagnostic, and therapeutic methods. This does not exclude the use of placebo, or no treatment, in studies where no proven prophylactic, diagnostic or therapeutic method exists[1].

30. At the conclusion of the study, every patient entered into the study should be assured of access to the best proven prophylactic, diagnostic and therapeutic methods identified by the study[2].

31. The physician should fully inform the patient which aspects of the care are related to the research. The refusal of a patient to participate in a study must never interfere with the patient-physician relationship.

32. In the treatment of a patient, where proven prophylactic, diagnostic and therapeutic methods do not exist or have been ineffective, the physician, with informed consent from the patient, must be free to use unproven or new prophylactic, diagnostic and therapeutic measures, if in the physician's judgement it offers hope of saving life, re-establishing health or alleviating suffering. Where possible, these measures should be made the object of research, designed to evaluate their safety and efficacy. In all cases, new information should be recorded and, where appropriate, published. The other relevant guidelines of this Declaration should be followed.

---

[1] **Note of clarification on paragraph 29 of the WMA Declaration of Helsinki**
The WMA hereby reaffirms its position that extreme care must be taken in making use of a placebo-controlled trial and that in general this methodology should only be used in the absence of existing proven therapy. However, a placebo-controlled trial may be ethically acceptable, even if proven therapy is available, under the following circumstances:

- Where for compelling and scientifically sound methodological reasons its use is necessary to determine the efficacy or safety of a prophylactic, diagnostic or therapeutic method; or

- Where a prophylactic, diagnostic or therapeutic method is being investigated for a minor condition and the patients who receive placebo will not be subject to any additional risk of serious or irreversible harm.

All other provisions of the Declaration of Helsinki must be adhered to, especially the need for appropriate ethical and scientific review.

[2] **Note of clarification on paragraph 30 of the WMA Declaration of Helsinki**
The WMA hereby reaffirms its position that it is necessary during the study planning process to identify post-trial access by study participants to prophylactic, diagnostic and therapeutic procedures identified as beneficial in the study or access to other appropriate care. Post-trial access arrangements or other care must be described in the study protocol so the ethical review committee may consider such arrangements during its review.

Attachment 9

Elements of Patient Informed Consent

Shin Poong Pharm/Medicines for Malaria Venture requires that all informed consent statements used in studies which they sponsor comply with the International Conference on Harmonization Good Clinical Practice guideline. To ensure compliance, the informed consent itemization listed below is provided to guide the Investigator in drafting an acceptable informed consent. Shin Poong Pharm/Medicines for Malaria Venture must review the proposed informed consent form prior to its submission to the ethics committee; alternatively, Shin Poong Pharmaceuticals/Medicines for Malaria Venture will supply to the Investigator a draft informed consent statement which may be submitted to the ethics committee.

Informed consent must include the following items:

- A statement that the study involves research.
- An explanation of the purposes of the research.
- The approximate number of patients involved in the study.
- The expected duration of the patient's participation.
- The study treatment(s) and the probability for random assignment to each treatment.
- Explanation of experimental procedures.
- The study procedures to be followed, including all invasive procedures.
- The patient's responsibilities.
- A description of any reasonably foreseeable risks or inconveniences to the patient and, if applicable, to an embryo, foetus, or nursing infant.
- A statement that the treatment may involve risks which are currently unforeseeable.
- The anticipated expenses, if any, to the patient for participating in the study.
- A description of the reasonably expected benefits. If there is no intended clinical benefit to the patient, this should be stated.
- The anticipated prorated payment, if any, to the patient for participating in the study.
- The alternative procedure(s) or course(s) of treatment that may be available to the patient, and their important potential benefits and risks.
- A statement that the patient or the patient's legally acceptable representative will be informed in a timely manner if information becomes available that may be relevant to the patient's willingness to continue participation in the study.
- An explanation as to whether any compensation or medical treatment is available if injury occurs. If so, what the compensation consists of and/or

where further information may be obtained.

- Whom to contact about information regarding the study.
- Whom to contact about the rights of patient's participating in clinical research (ideally not the Investigator).
- Whom to contact in the event of sustaining any study-related injury.
- A statement that the Sponsor and representatives will be granted direct access to the patient's original medical records for verification of clinical study procedures and/or data, without violating the confidentiality of the patient, to the extent permitted by the applicable laws and regulations and that, by signing a written consent form, the patient or the patient's legally acceptable representative is authorizing such access.
- A statement that the records identifying the patient will be kept confidential and, to the extent permitted by the applicable laws and/or regulations, will not be made publicly available. If the results of the study are published, the patient's identity will remain confidential.
- The foreseeable circumstances and/or reasons under which the patient's participation in the study may be terminated.
- Procedures for orderly termination of participation.
- A statement that participation is voluntary.
- A statement that refusal to participate will involve no penalty or loss of benefits.
- A statement that the patient may discontinue participation at any time without penalty or loss of benefits.
- A statement that a signed and dated copy of the consent is given to the patient.
- The statement, 'I agree to participate...'
- A place for the patient or the patient's legally acceptable representative to sign and date.
- A place for the signature and date of the person who conducted the informed consent discussion.
- Assent.

Attachment 10

Ethical Committee Review Form

ETHICAL COMMITTEE REVIEW FORM

Study Title:

Protocol Number:

Principal Investigator:

Institution/Clinical Site:

Please complete and return together with the EC opinion letter

Document	Title/description Version #/date	Date received	Approval status (please fill in)			
			YES	NO	Conditional	NA
Final Protocol			☐	☐	☐	☐
Protocol Amendment			☐	☐	☐	☐
Patient Information / Informed Consent Form			☐	☐	☐	☐
Investigator Brochure			☐	☐	☐	☐
CRF			☐	☐	☐	☐
Patient Diary Card			☐	☐	☐	☐
GP letter			☐	☐	☐	☐
Other:			☐	☐	☐	☐
Other:			☐	☐	☐	☐

Please also complete **the list of the members of the Ethical Committee and who reviewed and approved the Study**
Instructions: ***Please verify the date and version number of the documents you received. Check only one box for approval status, if the documents were received for reference only but not for approval (example: Investigator's Brochure), please check "NA"***

Medicines for Malaria Venture

Ethical Committee Membership list (voting members on this Study)

Name	Gender	Profession/Occupation	Affiliation/Institution
